# Supplementary material for: Patient and practitioner views on a combined face-to-face and digital intervention to support medication adherence in hypertension: a qualitative study within primary care
Source: BMJ Open. 2022 Feb 27;12(2):e053183. doi: 10.1136/bmjopen-2021-053183 (PMC8886486; doi:10.1136/bmjopen-2021-053183)
Supplement: Supplementary data [file bmjopen-2021-053183supp001.pdf]

Supplementary file 1: Consolidated criteria for reporting qualitative studies (COREQ): 32-item checklist

Item	Guide questions	Description	Reported on page no.
**Domain 1: Research team and reflexivity**			
Personal Characteristics			
1. Interviewer/facilitator	Which author/s conducted the interview or focus group?	One author (MVE) conducted the semi-structured interviews. Two authors co-facilitated the focus groups (MVE and JJ).	8, 28
2. Credentials	What were the researcher's credentials? E.g. PhD, MD	BSc (MVE), BSc, PhD (JJ)	N/A
3. Occupation	What was their occupation at the time of the study?	Research Assistant (MVE) and Research Associate (JJ)	N/A
4. Gender	Was the researcher male or female?	Female (MVE) and male (JJ)	N/A
5. Experience and training	What experience or training did the researcher have?	MVE's training in qualitative research has included: BSc undergraduate modules; Oxford University Introduction to Qualitative Research; University of Cambridge Social Sciences Research Methods Centre training course. JJ is experienced in conducting focus groups (JJ)	N/A
Relationship with participants			
6. Relationship established	Was a relationship established prior to study commencement?	Participants had no relationship with the researcher. Patients contacted MVE via telephone or email to arrange participation in the study. HCPs* met with MVE face-to-face during a set up meeting at the GP practice, prior to interview.	7-8

Van Emmenis M, *et al. BMJ Open* 2022; 12:e053183. doi: 10.1136/bmjopen-2021-053183

7. Participant knowledge of the interviewer	What did the participants know about the researcher? e.g. personal goals, reasons for doing the research	Participants knew the reasons for conducting the research, as detailed in the patient information sheet and discussed prior to commencing the interview/focus group.	N/A
8. Interviewer characteristics	What characteristics were reported about the interviewer/facilitator? e.g. Bias, assumptions, reasons and interests in the research topic	Participants were aware that the research aimed to inform the development of an intervention to support medication taking. Researchers were unable to avoid bias regarding prior knowledge of the research literature around medication adherence.	N/A
**Domain 2: study design**			
Theoretical framework			
9. Methodological orientation and Theory	What methodological orientation was stated to underpin the study? e.g. grounded theory, discourse analysis, ethnography, phenomenology, content analysis	Analysis was informed methodologically by the constant comparative approach[1] and theoretically by Perceptions and Practicalities Approach (PAPA)[2].	9
Participant selection			
10. Sampling	How were participants selected? e.g. purposive, convenience, consecutive, snowball	Patients were eligible if they were prescribed medication to treat hypertension and used SMS messages or smartphone apps. A random sample of patients were selected by GP practice administrators to receive the study invitation pack. HCPs were eligible if they were involved with the management or care of patients with hypertension, including medication reviews or hypertension checks. HCPs were a convenience sample from the four GP practices taking part in the study.	7-8

11. Method of approach	How were participants approached? e.g. face-to-face, telephone, mail, email	Patients received an invitation letter from their GP practice, or saw study advertisement posters in the waiting room. HCPs were invited to take part during a face-to-face meeting with the researcher (MVE) at their GP practice.	7-8
12. Sample size	How many participants were in the study?	20 patients and 11 HCPs	11
13. Non-participation	How many people refused to participate or dropped out? Reasons?	All participants who responded to the study invitation and set a date/time for participation, took part in either an interview or focus group.	N/A
Setting			
14. Setting of data collection	Where was the data collected? e.g. home, clinic, workplace	Patient interviews: participant's homes (n=4), workplace (N =1) and a local library (n=1).<br>HCP interviews: their place of work (n=11 HCP)<br>Focus groups: local community centres (n=14, across 4 groups)	9
15. Presence of non-participants	Was anyone else present besides the participants and researchers?	Only the participants and researchers were present during the study.	N/A
16. Description of sample	What are the important characteristics of the sample? e.g. demographic data, date	Participant characteristics are reported in Tables 1 and 2.	Tables 1 and 2
Data collection			
17. Interview guide	Were questions, prompts, guides provided by the authors? Was it pilot tested?	The interview and focus group schedules were guided by the principles of PAPA and informed by the aims of the intervention development team. Interview and focus group schedules were reviewed by the research team and PPI representatives.	9-10, supplementary files 2a, 2b, 2c
18. Repeat interviews	Were repeat interviews carried out? If yes, how	No repeat interviews were carried out.	N/A

Van Emmenis M, *et al. BMJ Open* 2022; 12:e053183. doi: 10.1136/bmjopen-2021-053183

	many?		
19. Audio/visual recording	Did the research use audio or visual recording to collect the data?	Interviews and focus groups were audio-recorded.	10
20. Field notes	Were field notes made during and/or after the interview or focus group?	Field notes were made during and/or after each interview and focus group.	N/A
21. Duration	What was the duration of the interviews or focus group?	Mean interview duration was 47 minutes (range 21 to 75). Mean focus group duration was 88 minutes (range 83 to 91).	10
22. Data saturation	Was data saturation discussed?	Recruitment of participants continued until no new themes were emerging in relation to the specific topics covered.	12
23. Transcripts returned	Were transcripts returned to participants for comment and/or correction?	Transcripts were not returned to participants for comment and/or correction	N/A
**Domain 3: analysis and findings**			
Data analysis			
24. Number of data coders	How many data coders coded the data?	Data was coded by MVE and JJ, supervised by HE. Findings were discussed with the research team.	10-11, 28
25. Description of the coding tree	Did authors provide a description of the coding tree?	A sample of transcripts were read thoroughly to identify broad codes. Transcripts were compared in turn with codes already identified to refine the coding framework. Codes were then grouped into themes.	N/A
26. Derivation of themes	Were themes identified in advance or derived from the data?	We generated general descriptive categories from a subsample of transcripts. These were either derived directly from the data, or were pre-defined from the literature review, interview schedule questions and/or aims of the intervention development. This meant that some	10

		categories and concepts had increased sensitivity.	
27. Software	What software, if applicable, was used to manage the data?	Data was managed using NVivo 12.	10
28. Participant checking	Did participants provide feedback on the findings?	Participants did not provide feedback on the findings.	N/A
Reporting			
29. Quotations presented	Were participant quotations presented to illustrate the themes/findings? Was each quotation identified? e.g. participant number	Participant quotations are presented in the main text to illustrate the findings. Each quotation is identified using a participant ID. A table of extra quotations from participants is presented in the supplementary materials.	See Results section, and Supplementary file 2.
30. Data and findings consistent	Was there consistency between the data presented and the findings?	There was consistency between the data presented and the findings.	See Results section.
31. Clarity of major themes	Were major themes clearly presented in the findings?	Major themes are identified at the beginning of the Results section, and referred to throughout.	See Results section.
32. Clarity of minor themes	Is there a description of diverse cases or discussion of minor themes?	Minor themes are explored alongside the major themes.	See Results section.

*** Note:** HCP = healthcare practitioner

Developed from: Tong A, Sainsbury P, Craig J. Consolidated criteria for reporting qualitative research (COREQ): a 32-item checklist for interviews and focus groups. *International Journal for Quality in Health Care*. 2007. Volume 19, Number 6: pp. 349 – 357

References

1. Charmaz K. *Constructing Grounded Theory: A Practical Guide through Qualitative Analysis*. SAGE; 2006.
2. Horne R, Cooper V, Wileman V, Chan A. Supporting Adherence to Medicines for Long-Term Conditions. *Eur Psychol*. 2019;24(1):82-96. doi:10.1027/1016-9040/a000353
