# Supplementary material for: Patient and practitioner views on a combined face-to-face and digital intervention to support medication adherence in hypertension: a qualitative study within primary care
Source: BMJ Open. 2022 Feb 27;12(2):e053183. doi: 10.1136/bmjopen-2021-053183 (PMC8886486; doi:10.1136/bmjopen-2021-053183)
Supplement: Supplementary data [file bmjopen-2021-053183supp002.pdf]

Supplementary file 2a

Example digital intervention messages

Medication reminder	Hello Jo, it's time to take your Lisinopril 20mg this morning. Thank you.
Feedback	Glad to see you're taking your blood pressure medication. Keep up the good work!
Information	Did you know that high blood pressure increases your risk of heart disease? Please take your tablets as prescribed.
Support	Have you taken all your prescribed tablets today? If you need support, reply 'HELP' to this number.

Note: Example messages were created as part of a wider NIHR-funded programme grant. For more information about the intervention content and functions, see Kassavou, A'Court, Chauhan et al (2020) https://pilotfeasibilitystudies.biomedcentral.com/articles/10.1186/s40814-020-00666-2

Topic guide: patient interviews

In the each interview, the interviewer will remind the participant of the project aims and check that they fully understand the participant information sheet, and will then take them through the informed consent process. Audio-recording of the interview will then begin.

Part 1: Questions to assess participant's experience with taking their medication

First I want to ask you about the medications that you're taking at the moment.

- How many medications have you been prescribed and what medications do you take?
  - How many doses do you need to take and how often?
- What would you say are the benefits of taking your medication?
- What about the downside of /problems with taking your medications?
  - How does this affect whether you take your medication or not?
- You said you're meant to take [XX] medications a day. How easy it is to remember to take them?
  E.g. prompts
  - Do you have a particular routine? If so, what is it?
- What about altering the dose, is this something that you ever do? [can you tell me more about that? Which medication? How do you alter it? Can you tell me why you alter it in that way? Can you think of any other reason why you don't take your tablets regularly? E.g. side effects]
- Is there anything that helps you to take you medication? Anything that makes it easier?

Part 2: Views on an intervention to support medication adherence

- Interviewer will outline the basic structure of the proposed intervention, using visual aid/schematic to help explain.

  - A very brief face-to-face session with a practice nurse or healthcare assistant followed by ongoing support via mobile/internet.

- How would you feel about something like this that could support you to take your medication as prescribed?
  - Via text message?
  - Via an app?
- Which of these would be best for you? [e.g. prefer app or text?]
- What sort of messages would you find most helpful for helping you to take your tablets?

Example materials

- *Show participants examples of digital interventions*
  - Prompt questions
    - Which types of messages would you find most useful for helping you to take your medications? Why/why not?
    - What types of messages would be unhelpful? And why?
  - How could these messages be improved? Or reworded?
  - What messages would be useful for someone who doesn't want to take their pills? (i.e. INA)
  - How many messages per day or per week?

  - What about information / any other features that could be included? *E.g. probes:*
    - Feedback on your adherence level (e.g. graph, table % score, which prefer?)
    - Involve a carer/significant other?
    - Make use of location information

Part 3 – User engagement

- What would encourage you to start using something like this? *(Prompt initiation ideas such as demonstrating SMS/app during consultation, downloading app during consultation)*
  - Is there anything that would prevent or make it difficult for you using it?
  - Would a tutorial help you to understand how to use the SMS/app? How would you prefer to access this tutorial?
  - What would encourage you to keep using the app/text service long term?

Supplementary file 2b

Topic guide: healthcare practitioner interviews

In the each interview, the interviewer will remind the participant of the project aims and check that they fully understand the participant information sheet, and will then take them through the informed consent process. Audio-recording of the interview will then begin.

Part 1: Questions to ascertain participant's experience with addressing non-adherence issues with their patients

- Can you describe your role in the GP Practice?
- How involved are you with prescribing and monitoring patients' medication?
- How do you usually ascertain whether a patient is taking their medication as prescribed?
- What do you tend to do if/when you think a patient is not taking their medication as prescribed?

    E.g. probes:

    - Medication-taking can be perceived as a sensitive topic, how do you tend to address this when starting your conversation?
    - Do you explore patients' reasons for not taking it; how easy/difficult is this?
    - What challenges do you come across?
    - What do you think can help support patients in their medication taking?

Part 2: Views on an intervention to support medication adherence

Interviewer will outline the basic structure of the proposed intervention, using visual aid/schematic to help explain.

- *A very brief face-to-face session with a practice nurse or healthcare assistant or pharmacist followed by (ii) ongoing support via mobile/internet.*
- *Include a draft outline of the 5 minute face-to-face consultation between HCP and patient.*

- How comfortable/confident would you feel in delivering this?

    E.g. probes:

    - *Go through draft outline of VBI line by line.*
        - E.g. tailoring questionnaire
        - What information would you include in the pamphlet?
        - Is there anything you would add/remove?
    - What about doing this all in 5 minutes?
    - Would you find this task demanding? If yes, how could we make this easier for you? (E.g. tools/prompts/scripts- and how best to present these- bullet points?)
    - What kind of training would you find helpful? (E.g. multiple sessions/feedback/group work)

Interviewer will outline the idea of a digital intervention [possibly using visual aid/schematic to help explain how/when this would happen]

- What are your thoughts on how patients would get on with an intervention like this?
    - How useful do you think it would be in supporting patients to take their medications between their primary care consultations?
        - *E.g. probes:* helpful/unhelpful messages
    - What do you think patients need from an intervention like this?
    - If you were designing text messages/phone app, what content or materials would you include?

Part 3: Incorporating into a consultation

- What would encourage you to recommend a text message service/app to your patients?
    - What thoughts do you have on encouraging patients to use it / and keeping them engaged?
    - Would you find it helpful to trial the digital intervention yourself before recommending to patients?
- How might an intervention be incorporated into a consultation at the GP practice?
    - Which routine consultation would be appropriate?
    - What thoughts do you have on how to sign the patients up?
- Are there any specific patients who might benefit from an intervention more, or less, than others?

Van Emmenis M, *et al. BMJ Open* 2022; 12:e053183. doi: 10.1136/bmjopen-2021-053183

Supplementary file 2c

Topic guide: patient focus groups

Introduction

- Consent etc. (audio recording - ensure participants are comfortable with this before starting, and 'ground rules' of FGs.)
- Begin audio recording.
- Describe the PAM intervention. Will keep brief and in lay terms.
- We want to find out what you think about some of the app features

- Before we start, can I ask how many medications you've each been prescribed?
    - How many per day?
    - Morning/evening doses?
- How much you use your smartphones?
- Do you use any Apps that have reminder message pop up (e.g. calendar, med reminder app etc)?
    - Prompt: do these have alerts? How often do you use these apps?

1. Reminder notification messages

I'd like you to look at these examples [medication reminder messages]

- Go through each feature for each scenario (the text, layout, image, size, etc.) what do you like/dislike? What would you change?

Message content

- Is there anything missing from this message?
- What would you remove from this message?
- Which is your favourite and why?

Snooze options

Do you know what a snooze option is (e.g. like your alarm clock) [if not explain it's a feature we can add which allows you to delay the notification for extra time, similar to an alarm clock.]

- Let's say you're able to snooze the medication reminder message. How useful would you find this function? In which situations might you use it?
    - [prompts could include how long snooze should be etc.]

Images of medication

- Would you find it useful to have any of these images of your medications in the App?
- Would you like to be able to take your own photo and upload it?

Types of medication

- If you have more than one medication….

  - How would you feel about receiving reminder messages ONLY for medication related to HBP?
  - Would you want the option to choose which medications you receive reminders for?
- Do your medications ever change? What about if your medications change- how would you like to handle that?
  - Update the app yourself?

Message timings

- What time would you like to receive these messages?
  - Would you want to be able to change the timings yourself?
  - How comfortable would you feel doing this?
  - How about weekday vs weekends?

Message frequency

- When talking about reminder messages, how many of these would you like to receive per day?
- What about for non-reminder messages? (E.g. messages with advice and support to motivate people to take their medication)

Engagement and 'honest' reporting!

- We know that some people can get fed up with these types of apps, and messages on your phone every day.
- What would encourage you to use the app every day, say for a month? Or 3 months?
  - What would discourage you?
- What about stopping/reducing reminders?
  - When/why might you want the messages reducing and/or stopping?

- Some people might get a notification telling them a dose is due, think, "I want to skip this dose".
- How honest do you think people would really be when responding in the app?
  - How can we encourage people to use it 'honestly'
    - Are there any occasions where people might 'lie'?
    - Prompts (e.g. assure people that their GP won't see what they say)

2. Feedback of adherence

Apps can give you feedback on your results using different visual formats (e.g. table, graph, list, monthly calendar – show examples)

Prompts:

- Which would you prefer? And why?
- Total % score – helpful?
- Would you use this feature?
- What information do you want to see in the 'feedback'?
- What other methods could the app use to feedback reports?

- Would you find weekly or monthly feedback more helpful? Why?

3. Sensing data

- Now we would like to hear your thoughts on 'sensing technology'.
- Do you know much about the sensors in our smartphones?

- *[Facilitator explains sensors]*
- *All smartphones have sensors built into the device, which can gather data on how the phone is being used, such as movement and light (e.g. detect if it's face up or down).*
- *A simple example is when you make a call, and hold the phone to your ear, do you notice that the touchscreen automatically switches off, to stop you pressing buttons with your cheek. This happens because a sensor within the phone can detect when an object is close to its screen.*
- *Another example is how some phones have an inbuilt pedometer which can count the number of steps walked each day, by detecting the 'walking' movement of your phone.*

- *[Explain sensors re tailoring the App]*
- *Sensing technology is a novel way to personalise apps to your everyday behaviour.*
- *E.g. with this app, we can use sensors to personalise the messages sent to you, based on information collected from sensors in your phone.*
- *Provide examples of data that could be collected by the app*
- *Location data.*
  - *Detected either by WiFi (e.g. your home WiFi or a café WiFi) or GPS (detecting the location of the phone, like when we use Google Maps and satnavs).*
  - *The app could use this to send you messages at appropriate times, e.g. only when you're at home.*
- *Movement of phone or pedometer*
  - *e.g. App will detect movement of the phone (running/walking) and will delay a reminder until you've finished*

- How would you feel about an app collecting this data?
  - Would it put you off using the app?
- Is there anything that we could do to encourage you to like/change your mind about this feature?
- Is there anything you don't like about this feature?
- What questions would you have about this? (this will help us to inform the leaflet for ppts)
- What information should we provide for users? How detailed? How? (e.g. website/leaflet)

Close
