# Supplementary material for: Patient and practitioner views on a combined face-to-face and digital intervention to support medication adherence in hypertension: a qualitative study within primary care
Source: BMJ Open. 2022 Feb 27;12(2):e053183. doi: 10.1136/bmjopen-2021-053183 (PMC8886486; doi:10.1136/bmjopen-2021-053183)
Supplement: Supplementary data [file bmjopen-2021-053183supp003.pdf]

Supplementary file 3

Draft outline of a 5-minute face-to-face consultation (VBI)

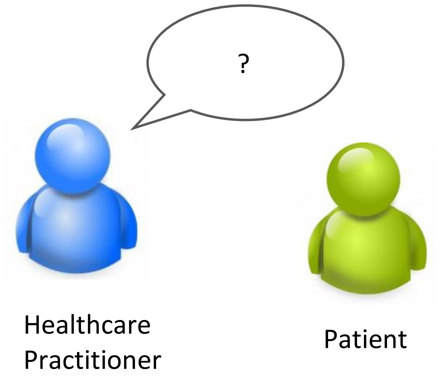

- Start the consultation with de-stigmatising medication non-adherence – forgetting to take tablets and/or having concerns is common.
- Support is available. Introduce the digital support (SMS texts or mobile phone app) as a (fun?) way to help patients take them – perhaps share personal experience with using it, demonstrate a key feature of the app/ text-messaging support.
- Complete short online questionnaire on behalf of patient:
    - Input basic information (patient name, age, mobile phone number)
    - Choose from drop down list of medications that patient is prescribed (tablet name, dose, frequency)
    - Ask patients to give their main reasons for not taking their medication (e.g. choose top 3 reasons from a list, or rank from most important to least important).
- Generate a code for patients to download the app/start the support. E.g. QR code, link to website for patients to download app.
- Hand out pamphlet with further information.
- Endorse the patients' use of digital support.
- Record in the system for follow-up purposes.

PAM HCP interviews VBI example
