# Supplementary material for: Patient and practitioner views on a combined face-to-face and digital intervention to support medication adherence in hypertension: a qualitative study within primary care
Source: BMJ Open. 2022 Feb 27;12(2):e053183. doi: 10.1136/bmjopen-2021-053183 (PMC8886486; doi:10.1136/bmjopen-2021-053183)
Supplement: Supplementary data [file bmjopen-2021-053183supp004.pdf]

Supplementary file 4: Major themes, sub themes and illustrative quotes

Theme	Sub theme	Example participant quote
**Reasons for non-adherence**	*Non intentional non-adherence (NINA)*<br>Forget to take medication and/or reorder prescription	If I don't put the blister pack back and take the tablets, sometimes I can't remember if I've taken it or not [P03, male, 60s]<br><br>I just totally forgot to take my medication this morning. I just had a cup of tea and a piece of toast. I have to admit I was in a hurry today, I'm sorry, I didn't take the BP [medication]. [P06, female, 60s]<br><br>Usually it's that they haven't had a chance to put their prescription in, so then they tend to run low. [HCP 07, Practice Nurse, female]
	Intentional non-adherence (INA)<br>Unpleasant side effects; general reluctance to take medication	Sometimes I'm tempted to, and if I'm doing some sporting activity, I will be less inclined to take them. They seem to reduce my performance […] perhaps take it after I've indulged in the sport. [P04, male, 40s]<br><br>Often patients will say that they felt fine before they took their blood pressure medicine and now they feel rotten. [HCP 06, Practice Nurse, female]<br><br>Especially with metformin, because it can often have some unpleasant side-effects, and very often people will stop taking it because of this, but they don't actually report that fact to us. [HCP 10, Practice Nurse, female]<br><br>You know, I'm sure there's a lot of natural stuff out there that could help me you know. I don't know what effect it's having inside. That's the trouble, you know…what, in fact, it's doing. [P02, male, 60s]<br><br>I just hate myself for having to take them… I just feel that there must be a natural way… I just feel that, you know, lifestyle choices would be a better way than taking tablets, and that's why I hate myself for having to do it. [P03, male, 60s]

Van Emmenis M, *et al. BMJ Open* 2022; 12:e053183. doi: 10.1136/bmjopen-2021-053183

**Recommendations for intervention content**	Simple reminders to take medication and to reorder prescription (NINA)	It is good about the reminder because I sometimes say to my husband, er, would you remind me and he doesn't because he's worse than I am. [P05, female, 70s]<br><br>I think that would be quite good if it could tell you when you last got your prescription and when your next prescription's due and send a reminder maybe a few days before to say. [FG3, male]<br><br>Do you know what would be handy, is the reminder to tell you when you're gonna run out. That would be good for me. [FG1, male]
	Messages containing information about medication: consequences of non-adherence (targeting INA) acceptable to HCPs but not to patients; preference for advice on how to follow up a missed dose	I guess they need a more educational approach...that's the kind of need to explore why they are choosing not to take it. Do they understand the consequences of not taking it? So the nature of the messaging might be quite sort of shock, 'if you do not take your aspirin you are at risk of having a heart attack', or something like that. (HCP 02, GP, male)<br><br>It's like preaching, and I don't need my phone to be beeping at me in order to preach a message. [FG4, female]<br><br>I don't know whether I could...whether it's okay to do a catch-up, like if I missed my Ramipril in the morning, can I take that and the Indapamide at night? Or would that be too high a dose in one go? So, you know, maybe that needs to be addressed in the texts [P03, male, 60s]<br><br>Other days I've forgotten completely and then I get home and I think, "Shall I take 'em now or shall I wait 'til tomorrow now?" I've done that once or twice. (M)<br>That is a problem, whether you can take it if you've forgotten. (F)<br>I want to know precisely, "Well, will that affect it big or a lot or not a lot or...? And is taking them all together more important than the hour?" […] It's the occasional expert knowledge that motivates people to stick with an app isn't it? […] It's the fact that if you could press a button and say, "What should I do now 'cos I've forgotten 'em?" might be just a great help. (M) [FG3]

	Feedback on adherence levels – simple statistics preferred	That could be a graph, couldn't it? Or a percentage. You know, 'well done', you know, 'you're ninety-five per cent this month'. [P03, male, 60s]<br><br>Even a weekly graph might be quite nice. Because, then you can see that, actually, when I started a week ago, I was taking fifty per cent…and now I'm going up, and you know, just a visual kind of thing for them. And, actually, now I'm on a hundred per cent, and next week I'm hundred per cent. [HCP 01, Practice Pharmacist, female]<br><br>For me, I think the answer probably is yes actually, because actually that really would be a... you know, a wake-up call, thinking, "God, [participant name], do you realise that's two days this week?" I mean, okay, I should be able to tell what I had but, you know –. [FG4, male]
	Signpost patients to contact their HCP	If you are having side-effects, please contact somebody, rather than just ignore it…. It could be it's the pharmacist, or the Practice Pharmacist rather than the community one. It's just about improving interactions with us, the pharmacist, reporting side-effects. [HCP 06, Practice Nurse, female]
**Tailoring the digital intervention**	Highly tailored intervention facilitates engagement with SMS messages and smartphone app	It's about tailoring the process to the patients' needs….What suits one is not going to suit the next twenty people. [HCP 06, Practice Nurse, female]<br><br>It would be nice to be a bit personalised, it makes you feel a bit special [FG1, male]<br><br>You can default [medication reminders] to all the days of the week being the same but it might be nice to be able to specify different times of the day if you really wanted to. [FG3, male]<br><br>I mean, could users perhaps make their own decision which, which [reminder message] version they want to have appear? [FG4, female]<br><br>How much margin is there to vary from person to person? So if I said I wanted the full works I could have them but perhaps somebody that didn't? [FG1, male]

	Barrier to engagement – if intervention is overcomplicated or difficult to use	A hundred ideas that you get but if it's not simple then, the app in the end—From personal experience it won't work, it needs to be simple for people to buy into it. [FG2, male]<br><br>But I think it has to be really simple 'cos, you know, the more you're delving into this, it's... it can get complicated, can't it? So people are just gonna want something easy. [FG3, female]
**Acceptability of sensing technology**	Facilitator – Potential for sensing technology to enhance user experience; flexible opt-in/out options	Another time that I might forget to take medication is if I've gone somewhere just overnight ... I might just forget to put it in and I think, oh, well, nothing I can do, I'll have to wait 24 hours or whatever, but if you put in your diary on your phone that you were going to then [the app] might say, "don't forget to pack your pills". [FG2, male]<br><br>I would be happy with the sensing and respond accordingly because I think we could fine tune some of it ourselves couldn't we? If it was personalised. [FG2, female]<br><br>But could people have an off button for that if they wanted, for that bit of the menu? [FG2, female]
	Barrier to acceptability – concerns about confidentiality of data; requests for information about data use	It's all about how that information is dissembled, isn't it, and so long as that information is within reason locked away somewhere [FG4, male]<br><br>I would definitely want to know you weren't passing my data onto anyone else, either for medical or marketing purposes [FG2, female]<br><br>I'd feel much happier using one that wasn't purely commercial as well. [FG2, female]

**Attitudes towards a VBI (HCP only)**	Barriers and facilitators to implementation: must be straightforward to incorporate and deliver within primary care; five minute time limit potentially unfeasible; sufficient training in delivery of VBI	It would have to be something that's very accessible on your desktop or as part of integrated into the clinical system. [HCP 03, Practice Nurse, female]<br><br>I think this is quite a straightforward process, as I say, because a lot of this we should be doing already and then it's the add on bit at the end. [HCP 06, Practice Nurse, female]<br><br>I don't think it's going to end at five minutes. I think it's probably going to go a bit more than that in some cases [HCP 05, Healthcare Assistant, female]<br><br>I'm quite chatty so they will probably just chat with me, 'cause I make them chat, but it could be easily done in five minutes if you were concise and just got on with it. HCP 08, Practice Nurse, female]<br><br>It would be really good that we've had the training on how to use the app, how to use the questionnaire… It might be useful even just to show us how you might do a consultation. You know, sometimes visually seeing something can be… Even just a video. [HCP 01, Practice Pharmacist, female]<br><br>Quite often what works well is just an almost practical session of: "This is what we're proposing. This is how you do the face to face consultation. This is what the apps look like" and then, you know, it then develops discussion. [HCP 06, Practice Nurse, female]<br><br>The more you do and the easier would become and you get more slick at it, wouldn't you… What we would need is a little bit more information on the type of app and text that you are going to try and set up. And maybe have a dummy run and see how comfortable we felt with it and if there was anything we could do to make it slightly more slicker and more professional… A video clip or something of somebody doing it so we would know how to approach it and address it and stuff like that, will probably be the best thing. [HCP 09, Practice Nurse, female]

Van Emmenis M, *et al. BMJ Open* 2022; 12:e053183. doi: 10.1136/bmjopen-2021-053183

	Recommendations for VBI: importance of HCP/patient relationship; understand the root cause of non-adherence and address patient concerns	If we have a very judgmental approach to care then we actually lose the patient's confidence and their respect and actually they won't come back at all. So that is just so important.[HCP 06, Practice Nurse, female]<br><br>You can get through if you put it in the right wording. I do find, I've been doing it for quite a few years now and I find I have got a good, you know, I'm not blowing my own trumpet, but I have got quite a good rapport with my patients [HCP 04, Healthcare Assistant, female]<br><br>I just try to keep it open and honest, and sort of gain their trust so they actually tell me why [they aren't taking their medications]. Most patients, if they know you or if they've dealt with you before, are quite happy to share… If they, um, don't think that they're going to get into trouble [laughs] for not taking it. [HCP 11, Practice Pharmacist, female]<br><br>It's about sort of exploring why, what their understanding is of the medications that they are taking and trying to get an understanding of whether they recognise the importance of compliance. So it's kind of just exploring in a deeper way as to why they've not been taking. [HCP 02, GP, male]<br><br>I tend to just try and get to the bottom of [non-adherence]… It's unpicking what the problems are and what their perception is and sometimes it's quite misguided and it's different from one person to another. [HCP 03, Practice Nurse, female]<br><br>The reasons why patients don't take their medication we should really be exploring in any case… our role in the face to face is actually we can explore some of these things a bit easier. [HCP 06, Practice Nurse, female]

Note: HCP = healthcare practitioner
