# Supplementary material for: Patient and practitioner views on a combined face-to-face and digital intervention to support medication adherence in hypertension: a qualitative study within primary care
Source: BMJ Open. 2022 Feb 27;12(2):e053183. doi: 10.1136/bmjopen-2021-053183 (PMC8886486; doi:10.1136/bmjopen-2021-053183)
Supplement: Author's manuscript [file bmjopen-2021-053183.draft_revisions.pdf]

BMJ Open is committed to open peer review. As part of this commitment we make the peer review history of every article we publish publicly available.

When an article is published we post the peer reviewers' comments and the authors' responses online. We also post the versions of the paper that were used during peer review. These are the versions that the peer review comments apply to.

The versions of the paper that follow are the versions that were submitted during the peer review process. They are not the versions of record or the final published versions. They should not be cited or distributed as the published version of this manuscript.

BMJ Open is an open access journal and the full, final, typeset and author-corrected version of record of the manuscript is available on our site with no access controls, subscription charges or pay-per-view fees (http://bmjopen.bmj.com).

If you have any questions on BMJ Open's open peer review process please email info.bmjopen@bmj.com

BMJ Open

A combined very brief face-to-face and digital intervention to support medication adherence in hypertension: patient and practitioner views

Journal:	*BMJ Open*
Manuscript ID	bmjopen-2021-053183
Article Type:	Original research
Date Submitted by the Author:	06-May-2021

Complete List of Authors:	Van Emmenis, Miranda; University of Cambridge, Public Health and Primary Care<br>JAMISON, JAMES; University of Cambridge, Public Health and Primary Care<br>Kassavou, Aikaterini; University of Cambridge, Public Health and Primary Care<br>Hardeman, Wendy; University of East Anglia, School of Health Sciences<br>Naughton, Felix; University of East Anglia Faculty of Medicine and Health Sciences, School of Health Sciences<br>A'Court, Charlotte; University of Cambridge, Public Health and Primary Care<br>Sutton, Stephen; University of Cambridge, Public Health and Primary Care<br>Eborall, Helen; The University of Edinburgh, Usher Institute

Keywords:	QUALITATIVE RESEARCH, Hypertension < CARDIOLOGY, PRIMARY CARE, PUBLIC HEALTH

SCHOLARONE™
Manuscripts

I, the Submitting Author has the right to grant and does grant on behalf of all authors of the Work (as defined in the below author licence), an exclusive licence and/or a non-exclusive licence for contributions from authors who are: i) UK Crown employees; ii) where BMJ has agreed a CC-BY licence shall apply, and/or iii) in accordance with the terms applicable for US Federal Government officers or employees acting as part of their official duties; on a worldwide, perpetual, irrevocable, royalty-free basis to BMJ Publishing Group Ltd ("BMJ") its licensees and where the relevant Journal is co-owned by BMJ to the co-owners of the Journal, to publish the Work in this journal and any other BMJ products and to exploit all rights, as set out in our licence.

The Submitting Author accepts and understands that any supply made under these terms is made by BMJ to the Submitting Author unless you are acting as an employee on behalf of your employer or a postgraduate student of an affiliated institution which is paying any applicable article publishing charge ("APC") for Open Access articles. Where the Submitting Author wishes to make the Work available on an Open Access basis (and intends to pay the relevant APC), the terms of reuse of such Open Access shall be governed by a Creative Commons licence – details of these licences and which Creative Commons licence will apply to this Work are set out in our licence referred to above.

Other than as permitted in any relevant BMJ Author's Self Archiving Policies, I confirm this Work has not been accepted for publication elsewhere, is not being considered for publication elsewhere and does not duplicate material already published. I confirm all authors consent to publication of this Work and authorise the granting of this licence.

For peer review only

ORIGINAL RESEARCH

A combined very brief face-to-face and digital intervention to support medication adherence in hypertension: patient and practitioner views

Miranda Van Emmenis[1], James Jamison[1], Aikaterini Kassavou[1], Wendy Hardeman[2], Felix Naughton[2], Charlotte A' Court[1], Stephen Sutton[1], Helen Eborall[3]

[1] Behavioural Science Group, University of Cambridge, United Kingdom

[2] School of Health Sciences, University of East Anglia, United Kingdom

[3] Usher Institute, University of Edinburgh, United Kingdom

Correspondence:

Miranda Van Emmenis

Behavioural Science Group, Primary Care Unit, East Forvie Building, Forvie Site, Robinson Way, Cambridge, CB2 0SR

Tel 01223 330524

Email mv404@medschl.cam.ac.uk

Word count: 5341

For peer review only

Abstract

Objectives: To explore patients' and healthcare practitioners' views about non-adherence to hypertension medication and potential content of a combined very brief face-to-face discussion (VBI) and digital intervention (DI).

Methods: A qualitative study (N=31): interviews with patients with hypertension (n=6) and healthcare practitioners (HCPs; n=11) and focus groups with patients with hypertension (n=14). Participants were recruited through general practices in Eastern England and London. Topic guides explored reasons for medication non-adherence and attitudes towards a potential intervention to support adherence. Stimuli to facilitate discussion included example SMS messages and smartphone app features, including mobile sensing. Analysis was informed methodologically by the constant comparative approach and theoretically by Perceptions and Practicalities Approach (PAPA).

Results: Participants' overarching explanations for non-adherence were non-intentional (forgetting) and intentional (concerns about side-effects, reluctance to medicate). These underpinned their views on intervention components: messages that targeted forgetting medication or obtaining prescriptions were considered more useful than messages providing information on consequences of non-adherence. Tailoring the DI to the individuals' needs, regarding timing and number of messages, was considered important for user engagement. Patients wanted control over the DI and information about data use associated with any location sensing. While the DI was considered limited in its potential to address intentional non-adherence, HCPs saw the potential for a VBI in addressing this gap, if conducted in a non-judgemental manner. Incorporating a VBI into routine primary care was considered feasible,

For peer review only

provided it complemented existing GP practice software and HCPs received sufficient training.

Conclusions:

A combined VBI-DI can potentially address intentional and non-intentional reasons for non-adherence to hypertension medication. For optimal engagement, recommendations from this work include a VBI conducted in a non-judgmental manner and focusing on non-intentional factors, followed by a DI that is easy-to-use, highly tailored, and with provision of data privacy details about any sensing technology used.

Keywords: medication adherence, qualitative research, high blood pressure, digital intervention, healthcare professional, very brief face-to-face intervention

For peer review only

Strengths and limitations of the study

- To our knowledge, this is among the first qualitative studies to gather patient views on the use of sensing technology such as Wi-Fi or GPS within a smartphone app to support medication adherence.
- The study sought the views of a range of healthcare practitioners on incorporating a very brief intervention for medication adherence into a primary care consultation, a topic not previously explored in-depth.
- The use of stimulus materials provided detailed and focused responses for specific intervention components such as feedback on adherence and content of messages.
- While the sample size was relatively small, the depth and focus of insights gained are sufficiently useful in informing the development and refinement of intervention components.

Introduction

Medication adherence, defined as the level to which an individual takes medication as intended by their healthcare prescriber, is a worldwide public health concern.[1] Non-adherence to blood pressure lowering medication is estimated at 41%, which is relatively high compared with many other medications.[2, 3] This is associated with increased risk of cardiovascular disease related morbidity and mortality.[4, 5] Given that high blood pressure is responsible for nearly 20% of deaths worldwide, non-adherence to antihypertensive treatment is a global health concern.[6]

For peer review only

Previous research into medication non-adherence has documented its complexity and multifaceted nature.[7, 8] Two broad categories within this are (i) non-intentional non-adherence, a passive process due to factors not directly within an individual's control, such as memory or access difficulties,[8, 9] and (ii) intentional non-adherence, a more deliberate action whereby an individual makes a conscious decision not to take their medication due to their perceptions about or experiences with their medication or condition.[9]

The multifaceted nature of non-adherence presents a challenge to those developing interventions to support adherence; for example, determining which factors to target, while balancing feasibility of delivery with likely effectiveness. Digital interventions (DIs) such as SMS text messaging or smartphone applications (apps) offer interactive, low cost and scalable methods of providing support to individuals for whom medication adherence is a challenge.

Evidence for the effectiveness of DIs in improving medication adherence is promising. In a recent systematic review of app-based interventions, patients using a smartphone app to support medication adherence for various health conditions were twice as likely to report taking their medications than those receiving usual care.[10] Specific to hypertension, DIs such as SMS messages, smartphone apps, email and Bluetooth blood pressure monitors have been shown to improve medication adherence and lower both diastolic and systolic blood pressure.[11, 12]

Incorporating sensing technology into smartphone apps potentially expands the scope of DIs further. Passive smartphone sensors can collect user location data via GPS or Wi-Fi to enable the delivery of real-time support [13], which is of particular relevance given non-intentional non-adherence is strongly influenced by a

For peer review only - http://bmjopen.bmj.com/site/about/guidelines.xhtml

person's physical environment.[14] Smartphone sensing technology has shown success in DIs across the domain of health and well-being,[15] however user acceptability of such technology in a smartphone app to support medication adherence is largely unknown.

While user acceptability is key to use of a DI, potential users first need to install and engage with the DI for it to provide benefit. Primary care professionals, such as practice nurses or community pharmacists are ideally placed for encouraging uptake of DIs for medication adherence, for example during a medication review or at the point of prescription collection. However, evidence is limited on how healthcare professionals can best promote the uptake of DIs for medication adherence. The acceptability of combining a DI with a very brief face-to-face intervention delivered by a healthcare professional to support medication adherence has also not been widely explored.

This study aimed to explore patients' and healthcare practitioners' views on i) non-adherence to hypertension medication and ii) a complex intervention designed to support medication adherence. Initial ideas for the intervention consisted of a very brief face-to-face discussion with a primary care provider, followed by ongoing support via a DI (SMS messages or smartphone app). Feedback from participants included preferred content of the intervention and factors likely to influence engagement.

Methods

This study is reported in line with the consolidated criteria for reporting qualitative research studies checklist (COREQ)[16], see supplementary file 1.

For peer review only - http://bmjopen.bmj.com/site/about/guidelines.xhtml

Design

We undertook a qualitative study using semi-structured interviews followed by focus groups.

Recruitment and sampling

Patients were recruited for interviews with the help of the Clinical Research Network from primary care practices based in the East of England (n=3) and East London (n=1). Patients were eligible to participate if they were: 1) prescribed at least one antihypertensive medication for at least the previous three months 2) deemed non-adherent according to GP practice records, with a blood pressure reading of over 140/90 mmHg and/or gaps in filling repeat prescriptions in the previous three months and 3) used either SMS or smartphone apps. The practice administrator at each site generated a list of prospective participants that met criteria 1 and 2, which was screened by a GP or practice nurse. Eligible patients at each practice received a study pack in the post consisting of an invitation letter and participant information sheet. Posters highlighting the study were also displayed in the GP practices. Patients interested in taking part were invited to contact the researcher (MVE) via telephone or email; at which point the researcher checked that all three eligibility criteria were met before scheduling an interview.

A convenience sample of healthcare practitioners were recruited from the four GP practices taking part in this study. Healthcare practitioners were eligible to be interviewed if they were involved in the care of patients with hypertension e.g. through medication reviews or blood pressure checks. The researcher provided information and invited healthcare practitioners to participate during the study set-up

For peer review only - http://bmjopen.bmj.com/site/about/guidelines.xhtml

meeting. The researcher contacted them a few days later to check willingness to participate and to schedule interviews for those who were interested.

Recruitment for focus groups followed that of the patient interviews. The eligibility criteria was widened to include patients prescribed medication for type 2 diabetes and who were familiar with using smartphone apps (i.e. SMS alone was not sufficient). The decision to cease individual interviews and switch to focus groups with patients was three-fold: responses from eligible patients were low, preliminary analysis from the interviews was adding little new information to findings from previous research, and our previous work has shown the usefulness of focus groups to gain views and feedback on format, content and structure of DIs.[17–19]

Data collection

Patient interviews were conducted by one researcher (MVE) at patients' home, workplace or local library. Interviews with healthcare practitioners were conducted at their place of work. Focus groups were conducted at community centres local to the patients' general practice and moderated by two researchers (MVE and JJ).

Interviews and focus groups were guided by flexible topic guides [20] developed by the research team (MVE, HE, JJ, KK, CA), drawing on the PAPA framework [14] and previous research experience in both the topic area and intervention development. Topic guides were reviewed by Patient and Public Involvement (PPI) representatives to ensure the questions were easy to understand and appropriate for the study objectives. Broadly, interview topics included: reasons for medication non-adherence, current practice of HCPs during medication-related consultations; and views on a potential SMS text message or smartphone app

For peer review only - http://bmjopen.bmj.com/site/about/guidelines.xhtml

intervention that could support adherence. Example intervention content included medication reminders, advice and support messages, and feedback on adherence. See supplementary file 2 for the topic guides and example DI content. HCPs were shown an example protocol for a very brief face-to-face discussion or 'VBI' to generate discussion (see supplementary file 3). Components of the VBI included: introducing the digital support to the patient and inputting basic patient information via an online questionnaire to generate the tailored digital support.

Focus groups followed similar topic guides to the interviews, focusing on attitudes towards smartphone apps in particular, including the acceptability of sensing technology such as location sensing. To prompt discussion and gain feedback, both interview and focus group participants viewed stimulus materials of example intervention messages, including medication reminders, and smartphone app features, including graphs and images (see Figure 1 for examples).

Written informed consent was taken in person by the researcher immediately prior to the interviews and focus groups commencing. All patients received a £20 voucher for taking part. Interviews and focus groups were audio-recorded and professionally transcribed verbatim. Interviews lasted on average 47 minutes and focus groups 1 hour and 28 minutes.

Data Analysis

Analysis was informed methodologically by the constant comparative approach[21] and theoretically by the Perceptions and Practicalities Approach (PAPA).[14] Interview transcripts were read and re-read to aid familiarisation and identify preliminary themes; these broad descriptive themes were formed into an initial coding framework related to barriers and facilitators to medication adherence

For peer review only - http://bmjopen.bmj.com/site/about/guidelines.xhtml

and a potential intervention. Each transcript was then coded systematically (MVE) using NVivo qualitative data-indexing software (version 12; QSR International) and the coding framework was refined throughout the process. The process was repeated for focus group transcripts; the coding framework was further expanded and refined, given the additional topics explored in the focus groups. A sample of interview and focus group transcripts were independently coded by a second researcher (JJ) to confirm and strengthen the validity of findings. Meetings between the research team (MVE, JJ, HE) facilitated data analysis including discussion of themes, sub-themes and the interrelationships.

Patient and public involvement

All study materials (participant information sheet, invitation letter, study poster, consent form, topic guides and stimulus materials) were reviewed by representatives from the Cambridge University Hospitals Patient and Public Involvement (PPI) panel. We made a number of changes to the study materials as a result of PPI input; adjusted the language to make the documents more accessible, ensured interview questions were sensitively worded and easy to understand from a patient perspective. PPI representative Jennifer Bostock provided input throughout the study and reviewed and commented on this manuscript.

Ethical approval

Ethical approval for this study was obtained from the West Midlands – Solihull Research Ethics Committee, as part of NHS Health Research Authority approvals (Reference: 18/WM/0050).

For peer review only - http://bmjopen.bmj.com/site/about/guidelines.xhtml

Results

Six interviews were conducted with patients prescribed medication for hypertension and 11 with healthcare practitioners. Four focus groups were then conducted with patients prescribed medication for hypertension and/or type 2 diabetes (3-5 per group, total N=14). Patient participant characteristics are reported in Table 1. Their mean age was 62.7 years (range 47 – 79 years), 60% identified as male and 85% as White British. Eighty percent of patients reported using both SMS and smartphone apps, with the remaining 20% using SMS text messages only. All patients self-reported having occasionally missed or skipped their medication in the previous 3 months. HCP participant characteristics are reported in Table 2; six practice nurses, two healthcare assistants, two practice pharmacists, one GP.

Table 1 Participant characteristics (patients)

Characteristics	(n)	%
**Gender**		
Female	8	40
Male	12	60
**Age (years)**		
41-50	2	10
51-60	4	20
61-70	11	55
71-80	3	15
**Ethnicity**		
Asian or Asian British – Indian	1	5
Asian or Asian British – Pakistani	1	5
Black or Black British – Caribbean	1	5
White British	17	85
**Phone use**		
SMS only	4	20
SMS and smartphone app	16	80
**Data collection method**		

For peer review only

Semi-structured interview	6	30
Focus group	14	70

Note: n=20

Table 2 Participant characteristics (healthcare practitioners) and GP practice demographics

**Characteristics**	**(n)**	**%**
**Healthcare practitioners**		
**Job role**		
General Practitioner	1	9
Healthcare Assistant	2	18
Practice Nurse	6	55
Practice Pharmacist	2	18
**Gender**		
Female	10	91
Male	1	9
**Years practising**		
≤10	5	46
11-20	2	18
21-30	2	18
≥31	2	18
**GP practices**		
**GP practice location**		
Urban	3	75
Rural	1	25
**GP practice IMD quintile**		
First quintile (least deprived)	2	50
Second quintile (less deprived)	1	25
Fourth quintile (more deprived)	1	25

Note: Healthcare practitioner n=11, GP practice n=4, IMD = Index of Multiple Deprivation, which ranks every small area in England from 1 (most deprived area) to 32,844 (least deprived). IMD scores of the 4 GP practices in this study: 9924, 25270, 26871, 30807.

To present the findings, we broadly categorise the key themes identified into the following categories: reasons for non-adherence, recommendations for message

For peer review only

For peer review only - http://bmjopen.bmj.com/site/about/guidelines.xhtml

content, tailoring the digital intervention, acceptability of sensing technology, and attitudes towards a VBI. We provide illustrative quotes below. See supplementary file 4 for additional quotes from participants. For reference, 'digital intervention' (DI) refers to both SMS text messages and smartphone app, as the same intervention messages can be delivered using both formats.

Reasons for non-adherence

Participants' provided two key explanations of non-adherence to antihypertensive medication. First, for *non-intentional* non-adherence, forgetting was the most common reported reason and was mentioned in three ways: forgetting to take medication, forgetting whether or not medication had been taken and forgetting to re-order prescription in time.

> *Sometimes you can't remember whether you have taken them already. And that can be problematic, so if someone asks you, you think, 'well, I don't know, maybe I have, maybe that was yesterday.' [P04, male, 40s]*

Second, in terms of *intentional* non-adherence, the experience or anticipation of side effects was a reason given for skipping, altering or delaying medication, as was the general reluctance to be reliant on medication.

> *I wish I could live without medication, I hate pumping my body with drugs. Sometimes I wonder, 'what side effects am I gonna have with this? Is it really benefitting me?' [P06, female, 60s]*

> *A lot of patients […have said], 'yes, the doctor has changed my medication, but they make me go funny, so I'm just going to take half or I've just, I'll just crush that and just take half instead of the two.' [HCP 05, Healthcare Assistant, female]*

These overarching explanations were apparent when participants discussed the merits of a digital intervention to improve adherence, as presented in the following sections. We begin with participants' views about a DI's messaging content, followed

For peer review only - http://bmjopen.bmj.com/site/about/guidelines.xhtml

by tailoring and then sensing technology; the final section considers the role of the VBI component.

Recommendations for message content

Simple reminder messages were perceived as useful for both taking medication and re-ordering prescriptions.

> *Even if I'm in a hurry, [when] I receive this reminder I [would] realise the importance. I think if I keep getting messages that would be very effective and definitely help me. Even if I'm tired and it would make me […] I'd force myself to get up and go and take the medication. [P06, female, 60s]*

> *It would be useful, if you're running out of tablets, to have some way of automatically reordering or a reminder to do that. So it's reminding you to take your tablets, and also when you're running low. [P04, male, 40s]*

Information-giving messages were only perceived as helpful by participants if providing advice when medication had been missed, for example the safest way to 'catch up' on missed dose.

> *There ought to be a button of 'I've forgotten them 'til now, which bits should I take?' That could be useful. [FG3, male]*

While HCP-participants recommended messages about the benefits of medication or the consequences of non-adherence, patient-participants considered these unhelpful and unnecessary, particularly if lack of knowledge was not a barrier to adherence.

> *"I know what the risk is [from not taking my medication]. I don't feel that I want it repeated, no". [P03, male, 60s]*

There was, however, some recognition that newly-diagnosed patients may find such information motivating:

> *If you're new to taking blood pressure tablets [information on consequences of non-adherence] would be good. I mean, us experienced people who've taken them for years most probably don't need reminding that if you don't take it, something serious is gonna happen to you. [FG3, male]*

For peer review only

The idea of receiving feedback on one's adherence, generated from self-report via SMS message or App, in a message of encouragement (e.g. *'Well done!')* was viewed as unnecessary. Participants were more receptive to schematic feedback in the form of a graph, score or percentage.

> *Some people might need that encouragement, but then again, it sounds a bit patronising to some people, doesn't it [laughs]? […] I think the percentage thing would give people pride, you know, 'oh, I've reached 100% [of taking my medication] this month, I feel really good about that'. Once a month I'd like to know what my score was for the month. I think that would probably be enough incentive for me personally. [P03, male, 60s]*

Regular smartphone users suggested that feedback in the form of a monthly calendar highlighting 'missed medication' days, could be useful for spotting a pattern and identifying the circumstances of those days that contributed to a missed dose. Moreover, participants suggested the potential for this to facilitate discussion with a healthcare professional too:

> *A: I think [the app] would also be good to take, when you have a medication review, to take to your GP so he or she can see what's going on as well.*
> *B: Yeah, help fix it by doing something, yeah*
> *[FG3, male (A, B)]*

Tailoring the digital intervention

Participants commented how they would be more likely to use, and continue to use, the DI if the messages were tailored to their preferences and their individual medication regime, in terms of frequency and timing of doses:

> *Some people are on medication once a day, twice a day, three, four. Could the app be tailor-made for the individual? And remind us accordingly? [FG2, female]*

> *A: That's why [the intervention] should be tailor-made for the individual patient. I think it's going to be critical really. Rather than a generic –*
> *B: And have options, yeah.*
> *A: Because if it's a generic app and it doesn't suit some people they won't use it or they won't respond to it. [FG1, male (A,B)]*

For peer review only - http://bmjopen.bmj.com/site/about/guidelines.xhtml

Participants noted the importance of the DI including *all* their prescribed medications, i.e. not just the hypertension ones.

> *I think it would need to be somewhat of a select or deselect, you know, 'take all' but you can un-tick the ones that you're not taking now. (FG4, female)*

> *A: I would do it as all one. Even if you're doing it principally motivated by blood pressure, in the sense it's, you're trying to remind us to take pills in general, aren't you, so you have to somehow-*
> *B: Yes, I think you want all of them there. [FG3, male (A), female (B)]*

To ensure that tailoring meets patients' preferences and medication regime, and the changes over time, participants highlighted the importance of patients having control over the DI's settings. For example, being able to change timings of reminders and adding in short term medication.

> *A: I think I'd like to put my own [medications] in. And then when you have a "short course" [of medication] as we say, I'll add that in as well. I'd rather be in charge of putting it in.*
> *B: Especially as some you have to have on an empty stomach, don't you?*
> *A: Yeah, so you could fiddle with your timings for that one. [FG2, female (A), male (B)]*

> *It's gotta be a dynamic thing. Medications change, dosages change, things get stopped, times may change, so I probably would see as an app which patients would be free to add and subtract. [HCP 02, GP, male]*

A 'snooze' function (similar to an alarm snooze) was well-received by participants, provided users could set their own parameters, e.g., length of snooze duration and maximum number of snoozes.

> *It would be good for me 'cos I'm often not home when I'm supposed to take them, so if you hit the 'snooze' for an hour or whatever you choose it to be, […]and it'll keep reminding me again and I'll take the tablets [when I'm home]. [FG1, male]*

For peer review only - http://bmjopen.bmj.com/site/about/guidelines.xhtml

A suggestion for tailoring by adding images of medications into the app raised more problems than benefits; participants pointed out that *“every time you get the medication, the box changes” [FG1]* and it was felt this would create confusion, rather than help.

Acceptability of sensing technology

Participants were initially wary about the incorporation of sensing technology, such as GPS or Wi-Fi to determine location, into an app, raising concerns about surveillance, typically referred to as “*Big Brother*” *[FG1, FG3]* watching them. Participants were more likely to accept sensing technology if the perceived benefits (such as tailoring medication reminders to their specific schedule and locations) outweighed concerns about data privacy.

> *It would make it impossible to forget ‘cos I’d just walk through the door and take ‘em. That would be brilliant. [FG1, male]*

Participants requested information to address these concerns, including: who has access to their data beyond the university (in particular, less trusted organisations such as insurance or marketing companies); where data are stored; and what happens in the event of hacking.

> *A: Who are you gonna share this with? That’s all I’m worried about […]*
> *B: It could be pretty valuable information for insurance companies to put their premiums up. [FG4, male (A), female (B)]*

> *I think it’d be more reassuring to know it was a medical body behind it or a university body behind it; it gives it some substance and credibility. [FG1, male]*

Participants wanted to retain personal control over the sensing function, with the ability to choose when the app tracks and records their location data as well as the

For peer review only - http://bmjopen.bmj.com/site/about/guidelines.xhtml

ability to opt in/out at any point.

> *I think it would be a case of opt-in because I think some people would think it an invasion of privacy. I mean, personally I think it's a good idea but, you see, some people wouldn't like it. [FG4, female]*

Discussions about sensing technology prompted participants to suggest further ideas for functions of an app. Participants in two focus groups suggested linking the sensing technology with the smartphone calendar, to proactively detect periods when away from home, triggering reminder messages to pack medication or re-order prescriptions.

> *The app ought to be able to detect [that] my calendar says, 'Away for the weekend.' So the app could say, "Ooh look, you're going away for the weekend" it could send me a message or something on the Friday to make sure I pack them. That's almost what I want to be reminded of. [FG3, male]*

Participants emphasised the need for additional features to be optional, recognising that over-complicating the DI risked disengagement from potential users.

> *I suppose it's a case though of getting sufficient ability to customise it against making it just too longwinded and complicated for people to be bothered. [FG4, female]*

> *I'm just trying to think of just the least steps possible for the patient, because just adding more things is going to make them less likely to use these sorts of things… It needs to just be easy for them. [HCP 01, Practice Pharmacist, female]*

Above all, participants emphasised the importance of the DI being user-friendly for the target group, many of whom may be less familiar with smartphones.

> *The caveat I suppose might be that those that tend to have the chronic diseases tend to be the older age group so they may not be so tech savvy. We've got some patients who don't use mobile phones even now. [HCP 02, GP, male]*

Attitudes towards a very brief face-to-face intervention (VBI)

For peer review only - http://bmjopen.bmj.com/site/about/guidelines.xhtml

Patient-participants' discussions about the DI functions largely focused on addressing non-intentional non-adherence - mainly forgetting. On the whole, they were sceptical about a DI's success in addressing intentional non-adherence:

> *"If they're not taking the tablets and they don't wanna take the tablets, why would they sign up for the app?" [FG1, male].*

HCP-participants suggested including messages encouraging users to contact their healthcare provider if experiencing problems with their medication.

> *That would be really useful in that if they're stopping it for any reason it needs to come up with a message to say, "Please make an appointment with your GP. There may be alternative medications available which would suit you and you need to make an appointment to discuss that". [HCP 03, Practice Nurse, female]*

However, a more promising way of addressing intentional non-adherence was highlighted in relation to the 5-minute VBI component prior to use of the DI. The VBI was presented as a way for HCPs to signpost patients to the DI and discuss medication taking behaviour. HCPs talked positively about how, if done in a non-judgemental way and by an HCP with an established rapport with the patient, this could foster open communication and a more constructive consultation.

> *"That's the important thing, when patients can relate to you and they can see that you're actually not judging them, they do tend to then engage better" [HCP 07, Practice Nurse, female]*

A key aspect of encouraging honest communication in the VBI would be acknowledging that it is acceptable to have concerns about being prescribed medication. HCPs recommended asking the patient to talk through these concerns and, if needed, book a follow-up consultation with a prescribing practitioner about changing medication.

> *Have a discussion with them as to what's been happening, what the issues are, how we can make it easier for them […] 'Is there a problem with it? Are you getting side-effects? Do you find it difficult to take?' And then we can explore some of the issues. What is really important is to sift through what the issues*

For peer review only

are. Our role in the face-to-face is actually we can explore some of these things a bit easier. [HCP 06, Practice Nurse, female]

All HCPs perceived the VBI element as feasible to deliver within primary care, recommending incorporating it alongside a medication review or blood pressure check. HCP's had two key provisos; training to help them deliver the VBI within the tight timing of five minutes, and a "*user-friendly*" template that could be incorporated in existing computer systems for inputting patient data to inform the subsequent DI. HCPs also noted the need for sufficient training in using the DI itself, given their role in encouraging its use in their patients following the VBI.

I think that will be important, that whoever is talking about the app needs to know how it works and how you use it… Because if somebody who is recommending it doesn't know how to use it then you're not gonna buy into it. [HCP 09, Practice Nurse, female]

For peer review only

Discussion

Summary of main findings

Patients prescribed antihypertensive medication and the healthcare practitioners that care for them highlighted nonintentional (forgetting) and intentional (side-effects, reluctance to medicate) reasons for their non-adherence. Participants felt a mobile digital intervention (DI) that provided simple medication reminders and feedback messages would help improve adherence. To facilitate engagement with the DI, participants recommended it was tailored to the needs of the individual and their medication regime as well as providing user control over the tailoring and other optional functions. The use of sensing technology within a smartphone app was acceptable to participants provided they received comprehensive information about the associated use and confidentiality of their data.

While the DI was considered limited in its potential to address intentional non-adherence, HCPs saw the potential for a brief face-to-face discussion (or 'VBI') with patients in addressing this gap. Incorporating a VBI into a routine primary care was considered feasible, if it could be integrated into existing practice software systems and if training were provided.

Strengths and limitations of the study

This study was conducted as development work with a target patient group to inform aspects of an intervention as part of a larger research programme.[22] This study draws on relevant theory [8, 9, 14] and is among the first qualitative studies to gather patient views on the use of sensing technology (e.g. Wi-Fi or GPS) in a smartphone app to support medication adherence (see also Kassavou and

colleagues[23]). Whilst advances in technology can provide additional features to smartphone apps, it is important to assess the intended user group's views of such technology before its implementation.[24]

We gained novel insights from a range of healthcare practitioners on the acceptability and feasibility of incorporating a VBI for medication adherence into a primary care consultation, a topic that has not been previously explored in-depth. Our findings can inform the development and implementation of a medication adherence VBI in primary care. The use of stimulus materials generated discussion in the interviews and focus groups, and provided focused responses for specific hypothetical intervention components.

We acknowledge that, with the relatively small sample size, the findings may be limited in their application to a patient and healthcare professional population. However, the depth and focus of insights gained provided rich data that were sufficiently useful in informing the development and refinement of intervention components for the wider programme, and to similar interventions.

We experienced challenges with recruiting patients through GP practices, particularly those who were non-adherent to their medication, a group who may be less likely to participate in a study of this nature. For future studies we would recommend widening recruitment methods to include patients not tied to a specific sample of GP practices, e.g., via social media channels or community groups.

Comparisons with existing literature

For peer review only - http://bmjopen.bmj.com/site/about/guidelines.xhtml

The findings echo previous research that has identified the main reasons for non-adherence to cardiovascular-related medication as forgetting and side-effects [25] [26] as well as the broad categorisation of reasons into intentional and non-intentional.[27, 28] In our study, this distinction was particularly helpful when considering which elements of an intervention were appropriate for targeting these two broad categories.

Participants with lived experience of hypertension saw little value in information-style messages (e.g. about the consequences of non-adherence) in addressing intentional non-adherence. Rather, they suggested that such messages may be most helpful for newly diagnosed patients, a finding which replicates previous research on digital interventions for cardiovascular medication. [29, 30] Similarly to previous studies [18], participants expressed concerns about receiving too many messages, suggesting this would influence (dis)engagement with the DI. Participants also emphasised the need for a DI to be as simple and easy-to-use as possible, another common theme in usability studies for medication adherence DIs, whereby difficulties with navigating a website, SMS or smartphone app have presented barriers to usage.[31, 32] Our findings support the need for usability testing with the DI target users.

Recommendations for an intervention to support medication adherence

The findings from this study have several implications for the development of a DI to support medication adherence. To encourage engagement with an intervention, it needs to be highly tailored to each individual. This includes: the timing and content of reminder messages (to address non-intentional non-adherence) and the content of support messages (for intentional non-adherence), where knowledge

For peer review only - http://bmjopen.bmj.com/site/about/guidelines.xhtml

and duration of health condition varies between individuals. Furthermore, a key tailoring variable as recommended by HCP-participants was the individual patient's specific barrier(s) to adherence.[33] Tailoring data can be collected using various methods, ideally before the start of the intervention for optimal impact. This could include a short questionnaire, in person or by phone with a practitioner, within a smartphone app, or via a set of SMS messages requiring responses.[18]

It was common for participants in this study to be taking multiple medications per day, and most wanted this to be reflected in the medication reminders. This requires a balance between providing appropriate adherence support without over-complicating the DI or overburdening the user, resulting in reduced intervention engagement.[34]

This study obtained novel insight from patients on the use of passive sensing technology within a medication adherence smartphone app. To increase the acceptability of sensing technology, future apps should explain the benefits that it can provide to the user, such as tailored medication reminders based on real-time location, or prompts to pack medication for upcoming holidays detected via calendar syncing. The app must provide a flexible opt in/out option for the collection of sensing data as well as information on how personal data will be used and stored within the app. Lastly, users may be more accepting of a location-sensing app created by a university or charity rather than a commercial company, which follows previous qualitative user testing into GPS-based smartphone apps for behaviour change. [23, 35, 36]

Primary care was viewed as an appropriate setting for HCPs to introduce patients to a DI and pair it with a brief behavioural face-to-face discussion, or 'VBI'.

For peer review only

More specifically, this could address intentional non-adherence by exploring the specific barriers to medication adherence with patients. Using a non-judgemental approach for this, would encourage patients' openness, which in turn would provide more useful information for tailoring the DI and possibly making adjustments to the patient's regime as part of the usual care. This supports a body of literature on shared decision-making [37] which has demonstrated an association between an improved patient-professional partnership and medication adherence - for a variety of conditions [38] and for hypertension specifically.[37, 39] Delivering a VBI requires skill, in order to incorporate all elements and within the short timeframe.[40, 41] Our study indicates the importance of incorporating the principles of shared decision-making into VBI training for healthcare professionals.

Conclusion

Overall, patients and healthcare practitioners saw the benefit of receiving medication reminders via SMS message or smartphone app. Intervention developers should consider an intervention that is highly tailored to the user, straightforward to use, and addresses data privacy concerns. The use of sensing technology in an adherence support smartphone app was acceptable to patients, therefore future research could investigate the feasibility of incorporating such technology into a smartphone app for adherence. A routine primary care consultation was viewed as an appropriate setting to introduce the DI to patients and discuss medication-taking behaviour with patients, but the feasibility of delivering it as 'very brief' i.e. under five minutes, should be explored further.

For peer review only - http://bmjopen.bmj.com/site/about/guidelines.xhtml

Supplementary material

S1 – COREQ checklist

S2 – Topic guides and sample of proposed intervention content

S3 – Example VBI protocol

S4 – Extra participant quotations

Figure 1 (legend)

Example digital intervention content to generate discussion in interviews and focus groups; medication reminder notification, feedback on medication adherence levels (weekly and monthly), SMS support message

Acknowledgements

This study was conducted on behalf of the Programme on Adherence to Medication team (see https://www.phpc.cam.ac.uk/pcu/research/research-projects-list/other-projects/pam/ for team members). We acknowledge and thank the following individuals for contributing to the development of the topic guides and visual prompts for this study: Jagmohan Chauhan and Sandra Servia, Department of Computer Science and Technology, University of Cambridge; Debi Bhattacharya, School of Pharmacy, University of East Anglia.

We thank the patients, healthcare practitioners, practice managers and administrative staff at the 4 GP practices who took part in this study. We are grateful to Patient and Public Involvement (PPI) representatives for reviewing the design and

For peer review only

For peer review only - http://bmjopen.bmj.com/site/about/guidelines.xhtml

ethics of this study. We thank PPI representative Jennifer Bostock for their input across the whole study and for reviewing the manuscript. We acknowledge the support of the National Institute for Health Research Clinical Research Network (NIHR CRN).

Author contributions

All authors made substantial contributions to the conception and design of the study. MVE conducted the interviews, co-facilitated the focus groups, conducted data analysis and drafted the manuscript. JJ co-facilitated the focus groups, contributed to data analysis and drafted the manuscript. HE provided supervision and guidance for data collection, contributed to data analysis and drafted the manuscript. SS, FN, WH, AK and CA critically revised the manuscript and provided intellectual input and expert advice. All authors have read and approved this manuscript.

Funding

This article presents independent research funded by the National Institute for Health Research (NIHR) under the Programme Grants for Applied Research programme [RP-PG-0615-20013]. The views expressed in this publication are those of the author(s) and not necessarily those of the NIHR or the Department of Health and Social Care.

Competing interests

None declared.

Ethics approval

This study was approved by the West Midlands – Solihull Research Ethics Committee, as part of NHS Health Research Authority approvals (Reference: 18/WM/0050).

For peer review only

Data availability statement

Data are available upon reasonable request from the corresponding author.

Abbreviations: COREQ, consolidated criteria for reporting qualitative research; DI, digital intervention; GP, general practitioner; HCP, healthcare practitioner; VBI, very brief intervention.

References

[1] Sabaté E, World Health Organization (eds). *Adherence to long-term therapies: evidence for action*. Geneva: World Health Organization, 2003.

[2] Chowdhury R, Khan H, Heydon E, et al. Adherence to cardiovascular therapy: a meta-analysis of prevalence and clinical consequences. *Eur Heart J* 2013; 34: 2940–2948.

[3] Gupta Pankaj, Patel Prashanth, Štrauch Branislav, et al. Risk Factors for Nonadherence to Antihypertensive Treatment. *Hypertension* 2017; 69: 1113–1120.

[4] Forouzanfar MH, Liu P, Roth GA, et al. Global Burden of Hypertension and Systolic Blood Pressure of at Least 110 to 115 mm Hg, 1990-2015. *JAMA* 2017; 317: 165–182.

[5] Mills KT, Stefanescu A, He J. The global epidemiology of hypertension. *Nat Rev Nephrol* 2020; 16: 223–237.

[6] Gakidou E, Afshin A, Abajobir AA, et al. Global, regional, and national comparative risk assessment of 84 behavioural, environmental and occupational, and metabolic risks or clusters of risks, 1990–2016: a systematic analysis for the Global Burden of Disease Study 2016. *The Lancet* 2017; 390: 1345–1422.

[7] Brown MT, Bussell JK. Medication Adherence: WHO Cares? *Mayo Clin Proc* 2011; 86: 304–314.

For peer review only

[8] Horne R, Weinman J, Barber N, et al. Concordance, adherence and compliance in medicine taking.

[9] Clifford S, Barber N, Horne R. Understanding different beliefs held by adherers, unintentional nonadherers, and intentional nonadherers: application of the Necessity-Concerns Framework. *J Psychosom Res* 2008; 64: 41–46.

[10] Armitage LC, Kassavou A, Sutton S. Do mobile device apps designed to support medication adherence demonstrate efficacy? A systematic review of randomised controlled trials, with meta-analysis. *BMJ Open* 2020; 10: e032045.

[11] McLean G, Band R, Saunderson K, et al. Digital interventions to promote self-management in adults with hypertension systematic review and meta-analysis. *J Hypertens* 2016; 34: 600–612.

[12] Xiong S, Berkhouse H, Schooler M, et al. Effectiveness of mHealth Interventions in Improving Medication Adherence Among People with Hypertension: a Systematic Review. *Curr Hypertens Rep* 2018; 20: 86.

[13] Hardeman W, Houghton J, Lane K, et al. A systematic review of just-in-time adaptive interventions (JITAIs) to promote physical activity. *Int J Behav Nutr Phys Act* 2019; 16: 31.

[14] Horne R, Cooper V, Wileman V, et al. Supporting Adherence to Medicines for Long-Term Conditions. *Eur Psychol* 2019; 24: 82–96.

[15] Cornet VP, Holden RJ. Systematic review of smartphone-based passive sensing for health and wellbeing. *J Biomed Inform* 2018; 77: 120–132.

[16] Tong A, Sainsbury P, Craig J. Consolidated criteria for reporting qualitative research (COREQ): a 32-item checklist for interviews and focus groups. *Int J Qual Health Care* 2007; 19: 349–357.

[17] Jamison J, Naughton F, Gilbert H, et al. Delivering Smoking Cessation Support by Mobile Phone Text Message: What Information do Smokers Want? A Focus Group Study. *J Appl Biobehav Res* 2013; 18: 1–23.

[18] Morton K, Sutton S, Hardeman W, et al. A Text-Messaging and Pedometer Program to Promote Physical Activity in People at High Risk of Type 2 Diabetes: The Development of the PROPELS Follow-On Support Program. *JMIR MHealth UHealth* 2015; 3: e105.

[19] Eborall H, Morton K. Use of Focus Groups in Developing Behavioural mHealth Interventions: A Critical Review. In: Barbour RS, Morgan DL (eds) *A New Era in Focus Group Research: Challenges, Innovation and Practice*. London: Palgrave Macmillan UK, pp. 109–127.

[20] Britten N. Qualitative Research: Qualitative interviews in medical research. *BMJ* 1995; 311: 251–253.

For peer review only

[21] Charmaz K. *Constructing Grounded Theory: A Practical Guide through Qualitative Analysis*. SAGE, 2006.

[22] PAM - Programme on Adherence to Medication. *Primary Care Unit*, https://www.phpc.cam.ac.uk/pcu/research/research-groups/bsg/research-themes/medication-adherence/pam-programme-on-adherence-to-medication/ (accessed 1 October 2020).

[23] Kassavou A, A'Court CE, Chauhan J, et al. Assessing the acceptability of a text messaging service and smartphone app to support patient adherence to medications prescribed for high blood pressure: a pilot study. *Pilot Feasibility Stud* 2020; 6: 134.

[24] National Institute for Health and Care Excellence. Evidence Standards Framework for Digital Health Technologies., https://www.nice.org.uk/Media/Default/About/what-we-do/our-programmes/evidence-standards-framework/digital-evidence-standards-framework.pdf (2019, accessed 16 July 2020).

[25] Kassavou A, Sutton S. Reasons for non-adherence to cardiometabolic medications, and acceptability of an interactive voice response intervention in patients with hypertension and type 2 diabetes in primary care: a qualitative study. *BMJ Open* 2017; 7: e015597.

[26] Leon N, Surender R, Bobrow K, et al. Improving treatment adherence for blood pressure lowering via mobile phone SMS-messages in South Africa: a qualitative evaluation of the SMS-text Adherence SuppoRt (StAR) trial. *BMC Fam Pract* 2015; 16: 80.

[27] Barber N. Should we consider non-compliance a medical error? *Qual Saf Health Care* 2002; 11: 81–84.

[28] Horne R, Chapman SCE, Parham R, et al. Understanding Patients' Adherence-Related Beliefs about Medicines Prescribed for Long-Term Conditions: A Meta-Analytic Review of the Necessity-Concerns Framework. *PLOS ONE* 2013; 8: e80633.

[29] Nelson LA, Mulvaney SA, Johnson KB, et al. mHealth Intervention Elements and User Characteristics Determine Utility: A Mixed-Methods Analysis. *Diabetes Technol Ther* 2017; 19: 9–17.

[30] Hirschey J, Bane S, Mansour M, et al. Evaluating the Usability and Usefulness of a Mobile App for Atrial Fibrillation Using Qualitative Methods: Exploratory Pilot Study. *JMIR Hum Factors* 2018; 5: e13.

[31] Grindrod KA, Li M, Gates A. Evaluating User Perceptions of Mobile Medication Management Applications With Older Adults: A Usability Study. *JMIR MHealth UHealth*; 2. Epub ahead of print 14 March 2014. DOI: 10.2196/mhealth.3048.

[32] Nelson LA, Bethune MC, Lagotte AE, et al. The Usability of Diabetes MAP: A Web-delivered Intervention for Improving Medication Adherence. *JMIR Hum Factors* 2016; 3: e13.

For peer review only

[33] Krousel-Wood M, Hyre A, Muntner P, et al. Methods to improve medication adherence in patients with hypertension: current status and future directions. *Curr Opin Cardiol* 2005; 20: 296–300.

[34] Szinay D, Jones A, Chadborn T, et al. Influences on the Uptake of and Engagement With Health and Well-Being Smartphone Apps: Systematic Review. *J Med Internet Res* 2020; 22: e17572.

[35] Naughton F, Hopewell S, Lathia N, et al. A Context-Sensing Mobile Phone App (Q Sense) for Smoking Cessation: A Mixed-Methods Study. *JMIR MHealth UHealth* 2016; 4: e106.

[36] Boonstra TW, Nicholas J, Wong QJ, et al. Using Mobile Phone Sensor Technology for Mental Health Research: Integrated Analysis to Identify Hidden Challenges and Potential Solutions. *J Med Internet Res* 2018; 20: e10131.

[37] Fortuna RJ, Nagel AK, Rocco TA, et al. Patient Experience With Care and Its Association With Adherence to Hypertension Medications. *Am J Hypertens* 2018; 31: 340–345.

[38] Haskard Zolnierek KB, DiMatteo MR. Physician Communication and Patient Adherence to Treatment: A Meta-Analysis. *Med Care* 2009; 47: 826–834.

[39] Harmon G, Lefante J, Krousel-Wood M. Overcoming barriers: the role of providers in improving patient adherence to antihypertensive medications. *Curr Opin Cardiol* 2006; 21: 310–315.

[40] 1 Recommendations | Behaviour change: individual approaches | Guidance | NICE, https://www.nice.org.uk/guidance/ph49/chapter/1-Recommendations (accessed 4 May 2021).

[41] Carson KV, Verbiest ME, Crone MR, et al. Training health professionals in smoking cessation. *Cochrane Database Syst Rev*. Epub ahead of print 2012. DOI: 10.1002/14651858.CD000214.pub2.

For peer review only

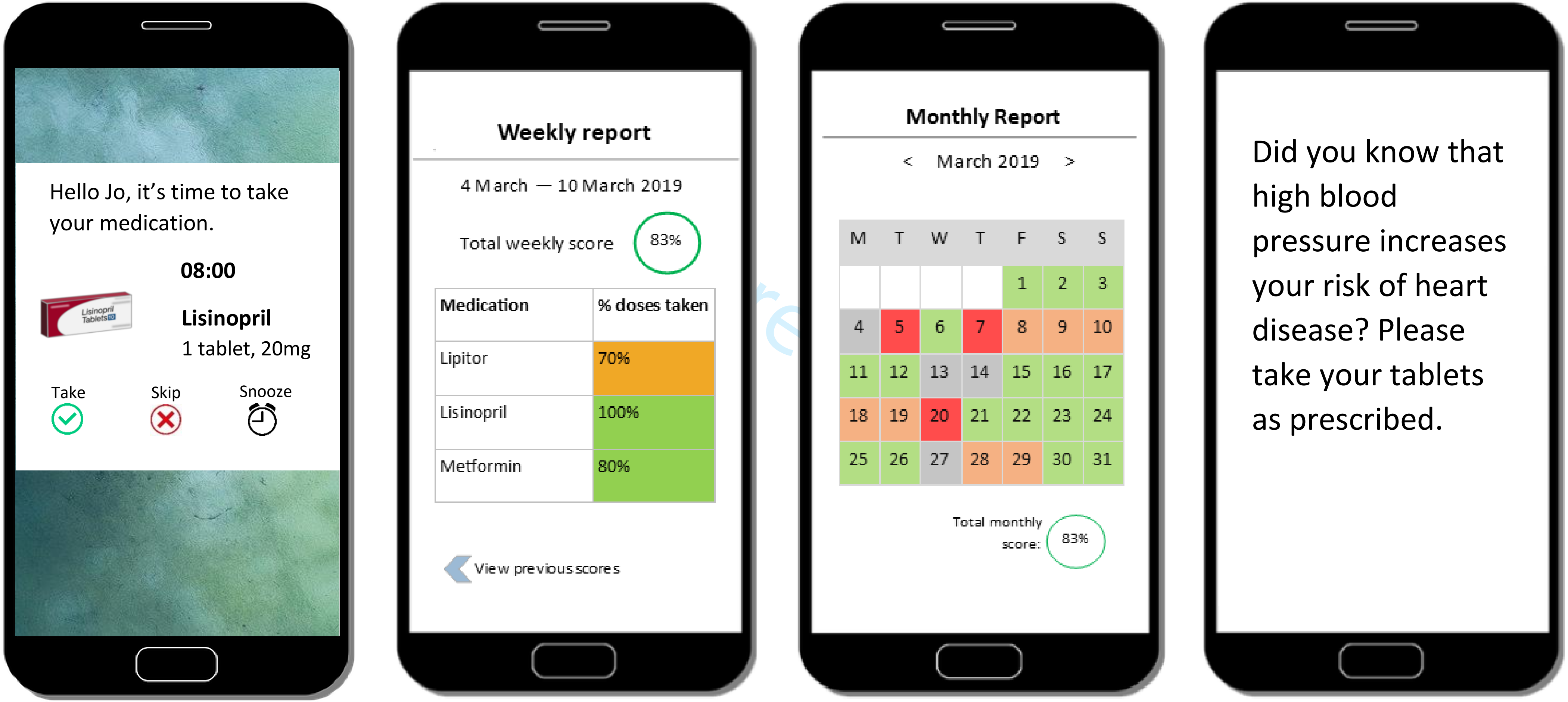
Hello Jo, it's time to take your medication.
08:00
Lisinopril
1 tablet, 20mg
Take
Skip
Snooze
Weekly report
4 March — 10 March 2019
Total weekly score
83%
Medication
% doses taken
Lipitor
70%
Lisinopril
100%
Metformin
80%
View previous scores
Monthly Report
< March 2019 >
M T W T F S S
1 2 3 4 5 6 7 8 9 10 11 12 13 14 15 16 17 18 19 20 21 22 23 24 25 26 27 28 29 30 31
Total monthly score:
83%
Did you know that high blood pressure increases your risk of heart disease? Please take your tablets as prescribed.

Supplementary file 1: Consolidated criteria for reporting qualitative studies (COREQ): 32-item checklist

**Item**	**Guide questions**	**Description**	**Reported on page no.**
**Domain 1: Research team and reflexivity**			
Personal Characteristics			
1. Interviewer/facilitator	Which author/s conducted the interview or focus group?	One author (MVE) conducted the semi-structured interviews. Two authors co-facilitated the focus groups (MVE and JJ).	8, 27
2. Credentials	What were the researcher's credentials? E.g. PhD, MD	BSc (MVE), BSc, PhD (JJ)	N/A
3. Occupation	What was their occupation at the time of the study?	Research Assistant (MVE) and Research Associate (JJ)	N/A
4. Gender	Was the researcher male or female?	Female (MVE) and male (JJ)	N/A
5. Experience and training	What experience or training did the researcher have?	Trained in qualitative methods and design (MVE), experienced in conducting focus groups (JJ)	N/A
Relationship with participants			
6. Relationship established	Was a relationship established prior to study commencement?	Participants had no relationship with the researcher. Patients contacted MVE via telephone or email to arrange participation in the study. HCPs* met with MVE face-to-face during a set up meeting at the GP practice, prior to interview.	7-8
7. Participant knowledge of the interviewer	What did the participants know about the researcher? e.g. personal goals, reasons for doing the research	Participants knew the reasons for conducting the research, as detailed in the patient information sheet and discussed prior to commencing the interview/focus group.	N/A

For peer review only - http://bmjopen.bmj.com/site/about/guidelines.xhtml

8. Interviewer characteristics	What characteristics were reported about the interviewer/facilitator? e.g. Bias, assumptions, reasons and interests in the research topic	Participants were aware that the research aimed to inform the development of an intervention to support medication taking. Researchers were unable to avoid bias regarding prior knowledge of the research literature around medication adherence.	N/A
**Domain 2: study design**			
Theoretical framework			
9. Methodological orientation and Theory	What methodological orientation was stated to underpin the study? e.g. grounded theory, discourse analysis, ethnography, phenomenology, content analysis	Analysis was informed methodologically by the constant comparative approach[1] and theoretically by Perceptions and Practicalities Approach (PAPA)[2].	9
Participant selection			
10. Sampling	How were participants selected? e.g. purposive, convenience, consecutive, snowball	Patients were eligible if they were prescribed medication to treat hypertension and used SMS messages or smartphone apps. A random sample of patients were selected by GP practice administrators to receive the study invitation pack. HCPs were eligible if they were involved with the management or care of patients with hypertension, including medication reviews or hypertension checks.	7
11. Method of approach	How were participants approached? e.g. face-to-face, telephone, mail, email	Patients received an invitation letter from their GP practice, or saw study advertisement posters in the waiting room. HCPs were invited to take part during a face-to-face meeting with the researcher (MVE) at their GP practice.	7
12. Sample size	How many participants were in the study?	20 patients and 11 HCPs	11

For peer review only - http://bmjopen.bmj.com/site/about/guidelines.xhtml

13. Non-participation	How many people refused to participate or dropped out? Reasons?	All participants who responded to the study invitation and set a date/time for participation, took part in either an interview or focus group.	N/A
Setting			
14. Setting of data collection	Where was the data collected? e.g. home, clinic, workplace	Patient interviews: participant's homes (n=4), workplace (N =1) and a local library (n=1).<br>HCP interviews: their place of work (n=11 HCP)<br>Focus groups: local community centres (n=14, across 4 groups)	8
15. Presence of non-participants	Was anyone else present besides the participants and researchers?	Only the participants and researchers were present during the study.	N/A
16. Description of sample	What are the important characteristics of the sample? e.g. demographic data, date	Participant characteristics are reported in Tables 1 and 2.	Tables 1 and 2
Data collection			
17. Interview guide	Were questions, prompts, guides provided by the authors? Was it pilot tested?	The interview and focus group schedules were guided by the principles of PAPA and informed by the aims of the intervention development team. Interview and focus group schedules were reviewed by the research team and PPI representatives.	9-10, supplementary files 2a, 2b, 2c
18. Repeat interviews	Were repeat interviews carried out? If yes, how many?	No repeat interviews were carried out.	N/A
19. Audio/visual recording	Did the research use audio or visual recording to collect the data?	Interviews and focus groups were audio-recorded.	9
20. Field notes	Were field notes made during and/or after the interview or focus group?	Field notes were made during and/or after each interview and focus group.	N/A

For peer review only

21. Duration	What was the duration of the interviews or focus group?	Mean interview duration was 47 minutes (range 21 to 75). Mean focus group duration was 88 minutes (range 83 to 91).	10
22. Data saturation	Was data saturation discussed?	Recruitment of participants to interviews and focus groups continued until we gained a full understanding of the participants' perspective. Data saturation was reached around the fourth focus group, whereby no new themes were emerging.	8
23. Transcripts returned	Were transcripts returned to participants for comment and/or correction?	Transcripts were not returned to participants for comment and/or correction	N/A
**Domain 3: analysis and findings**			
Data analysis			
24. Number of data coders	How many data coders coded the data?	Data was coded by MVE and JJ, supervised by HE. Findings were discussed with the research team.	10, 27
25. Description of the coding tree	Did authors provide a description of the coding tree?	A sample of transcripts were read thoroughly to identify broad codes. Transcripts were compared in turn with codes already identified to refine the coding framework. Codes were then grouped into themes.	N/A
26. Derivation of themes	Were themes identified in advance or derived from the data?	We generated general descriptive categories from a subsample of transcripts. These were either derived directly from the data, or were pre-defined from the literature review, interview schedule questions and/or aims of the intervention development. This meant that some categories and concepts had increased sensitivity.	9-10
27. Software	What software, if applicable, was used to manage the data?	Data was managed using NVivo 12.	10

For peer review only

28. Participant checking	Did participants provide feedback on the findings?	Participants did not provide feedback on the findings.	N/A
Reporting			
29. Quotations presented	Were participant quotations presented to illustrate the themes/findings? Was each quotation identified? e.g. participant number	Participant quotations are presented in the main text to illustrate the findings. Each quotation is identified using a participant ID. A table of extra quotations from participants is presented in the supplementary materials.	See Results section, and Supplementary file 2.
30. Data and findings consistent	Was there consistency between the data presented and the findings?	There was consistency between the data presented and the findings.	See Results section.
31. Clarity of major themes	Were major themes clearly presented in the findings?	Major themes are identified at the beginning of the Results section, and referred to throughout.	See Results section.
32. Clarity of minor themes	Is there a description of diverse cases or discussion of minor themes?	Minor themes are explored alongside the major themes.	See Results section.

* **Note:** HCP = healthcare practitioner

Developed from: Tong A, Sainsbury P, Craig J. Consolidated criteria for reporting qualitative research (COREQ): a 32-item checklist for interviews and focus groups. *International Journal for Quality in Health Care*. 2007. Volume 19, Number 6: pp. 349 – 357

References

1. Charmaz K. *Constructing Grounded Theory: A Practical Guide through Qualitative Analysis*. SAGE; 2006.
2. Horne R, Cooper V, Wileman V, Chan A. Supporting Adherence to Medicines for Long-Term Conditions. *Eur Psychol*. 2019;24(1):82-96. doi:10.1027/1016-9040/a000353

For peer review only - http://bmjopen.bmj.com/site/about/guidelines.xhtml

Supplementary file 2a

Example digital intervention messages

Medication reminder	Hello Jo, it's time to take your Lisinopril 20mg this morning. Thank you.
Feedback	Glad to see you're taking your blood pressure medication. Keep up the good work!
Information	Did you know that high blood pressure increases your risk of heart disease? Please take your tablets as prescribed.
Support	Have you taken all your prescribed tablets today? If you need support, reply 'HELP' to this number.

Note: Example messages were created as part of a wider NIHR-funded programme grant. For more information about the intervention content and functions, see Kassavou, A'Court, Chauhan et al (2020) https://pilotfeasibilitystudies.biomedcentral.com/articles/10.1186/s40814-020-00666-2

Topic guide: patient interviews

In the each interview, the interviewer will remind the participant of the project aims and check that they fully understand the participant information sheet, and will then take them through the informed consent process. Audio-recording of the interview will then begin.

Part 1: Questions to assess participant's experience with taking their medication

First I want to ask you about the medications that you're taking at the moment.

- How many medications have you been prescribed and what medications do you take?
  - How many doses do you need to take and how often?
- What would you say are the benefits of taking your medication?
- What about the downside of /problems with taking your medications?
  - How does this affect whether you take your medication or not?
- You said you're meant to take [XX] medications a day. How easy it is to remember to take them?
  E.g. prompts
  - Do you have a particular routine? If so, what is it?
- What about altering the dose, is this something that you ever do? [can you tell me more about that? Which medication? How do you alter it? Can you tell me why you alter it in that way? Can you think of any other reason why you don't take your tablets regularly? E.g. side effects]
- Is there anything that helps you to take you medication? Anything that makes it easier?

Part 2: Views on an intervention to support medication adherence

- Interviewer will outline the basic structure of the proposed intervention, using visual aid/schematic to help explain.

For peer review only - http://bmjopen.bmj.com/site/about/guidelines.xhtml

- A very brief face-to-face session with a practice nurse or healthcare assistant followed by ongoing support via mobile/internet.

- How would you feel about something like this that could support you to take your medication as prescribed?
  - Via text message?
  - Via an app?
- Which of these would be best for you? [e.g. prefer app or text?]
- What sort of messages would you find most helpful for helping you to take your tablets?

Example materials

- *Show participants examples of digital interventions*
  - Prompt questions
    - Which types of messages would you find most useful for helping you to take your medications? Why/why not?
    - What types of messages would be unhelpful? And why?
  - How could these messages be improved? Or reworded?
  - What messages would be useful for someone who doesn't want to take their pills? (i.e. INA)
  - How many messages per day or per week?

  - What about information / any other features that could be included? *E.g. probes:*
    - Feedback on your adherence level (e.g. graph, table % score, which prefer?)
    - Involve a carer/significant other?
    - Make use of location information

Part 3 – User engagement

- What would encourage you to start using something like this? *(Prompt initiation ideas such as demonstrating SMS/app during consultation, downloading app during consultation)*
  - Is there anything that would prevent or make it difficult for you using it?
  - Would a tutorial help you to understand how to use the SMS/app? How would you prefer to access this tutorial?
  - What would encourage you to keep using the app/text service long term?

For peer review only - http://bmjopen.bmj.com/site/about/guidelines.xhtml

Supplementary file 2b

Topic guide: healthcare practitioner interviews

In the each interview, the interviewer will remind the participant of the project aims and check that they fully understand the participant information sheet, and will then take them through the informed consent process. Audio-recording of the interview will then begin.

Part 1: Questions to ascertain participant's experience with addressing non-adherence issues with their patients

- Can you describe your role in the GP Practice?
- How involved are you with prescribing and monitoring patients' medication?
- How do you usually ascertain whether a patient is taking their medication as prescribed?
- What do you tend to do if/when you think a patient is not taking their medication as prescribed?

  E.g. probes:

  - Medication-taking can be perceived as a sensitive topic, how do you tend to address this when starting your conversation?
  - Do you explore patients' reasons for not taking it; how easy/difficult is this?
  - What challenges do you come across?
  - What do you think can help support patients in their medication taking?

Part 2: Views on an intervention to support medication adherence

Interviewer will outline the basic structure of the proposed intervention, using visual aid/schematic to help explain.

  - *A very brief face-to-face session with a practice nurse or healthcare assistant or pharmacist followed by (ii) ongoing support via mobile/internet.*
  - *Include a draft outline of the 5 minute face-to-face consultation between HCP and patient.*

- How comfortable/confident would you feel in delivering this?

  E.g. probes:

  - *Go through draft outline of VBI line by line.*
    - E.g. tailoring questionnaire
    - What information would you include in the pamphlet?
    - Is there anything you would add/remove?
  - What about doing this all in 5 minutes?
  - Would you find this task demanding? If yes, how could we make this easier for you? (E.g. tools/prompts/scripts- and how best to present these- bullet points?)
  - What kind of training would you find helpful? (E.g. multiple sessions/feedback/group work)

For peer review only - http://bmjopen.bmj.com/site/about/guidelines.xhtml

Interviewer will outline the idea of a digital intervention [possibly using visual aid/schematic to help explain how/when this would happen]

- What are your thoughts on how patients would get on with an intervention like this?
    - How useful do you think it would be in supporting patients to take their medications between their primary care consultations?
        - *E.g. probes:* helpful/unhelpful messages
    - What do you think patients need from an intervention like this?
    - If you were designing text messages/phone app, what content or materials would you include?

Part 3: Incorporating into a consultation

- What would encourage you to recommend a text message service/app to your patients?
    - What thoughts do you have on encouraging patients to use it / and keeping them engaged?
    - Would you find it helpful to trial the digital intervention yourself before recommending to patients?
- How might an intervention be incorporated into a consultation at the GP practice?
    - Which routine consultation would be appropriate?
    - What thoughts do you have on how to sign the patients up?
- Are there any specific patients who might benefit from an intervention more, or less, than others?

For peer review only

For peer review only - http://bmjopen.bmj.com/site/about/guidelines.xhtml

Supplementary file 2c

Topic guide: patient focus groups

Introduction

- Consent etc. (audio recording - ensure participants are comfortable with this before starting, and 'ground rules' of FGs.)
- Begin audio recording.
- Describe the PAM intervention. Will keep brief and in lay terms.
- We want to find out what you think about some of the app features

- Before we start, can I ask how many medications you've each been prescribed?
    - How many per day?
    - Morning/evening doses?
- How much you use your smartphones?
- Do you use any Apps that have reminder message pop up (e.g. calendar, med reminder app etc)?
    - Prompt: do these have alerts? How often do you use these apps?

1. Reminder notification messages

I'd like you to look at these examples [medication reminder messages]

- Go through each feature for each scenario (the text, layout, image, size, etc.) what do you like/dislike? What would you change?

Message content

- Is there anything missing from this message?
- What would you remove from this message?
- Which is your favourite and why?

Snooze options

Do you know what a snooze option is (e.g. like your alarm clock) [if not explain it's a feature we can add which allows you to delay the notification for extra time, similar to an alarm clock.]

- Let's say you're able to snooze the medication reminder message. How useful would you find this function? In which situations might you use it?
    - [prompts could include how long snooze should be etc.]

Images of medication

- Would you find it useful to have any of these images of your medications in the App?
- Would you like to be able to take your own photo and upload it?

Types of medication

- If you have more than one medication….
    - How would you feel about receiving reminder messages ONLY for medication related to HBP?

For peer review only - http://bmjopen.bmj.com/site/about/guidelines.xhtml

  - Would you want the option to choose which medications you receive reminders for?
- Do your medications ever change? What about if your medications change- how would you like to handle that?
  - Update the app yourself?

Message timings

- What time would you like to receive these messages?
  - Would you want to be able to change the timings yourself?
  - How comfortable would you feel doing this?
  - How about weekday vs weekends?

Message frequency

- When talking about reminder messages, how many of these would you like to receive per day?
- What about for non-reminder messages? (E.g. messages with advice and support to motivate people to take their medication)

Engagement and 'honest' reporting!

- We know that some people can get fed up with these types of apps, and messages on your phone every day.
- What would encourage you to use the app every day, say for a month? Or 3 months?
  - What would discourage you?
- What about stopping/reducing reminders?
  - When/why might you want the messages reducing and/or stopping?

- Some people might get a notification telling them a dose is due, think, "I want to skip this dose".
- How honest do you think people would really be when responding in the app?
  - How can we encourage people to use it 'honestly'
    - Are there any occasions where people might 'lie'?
    - Prompts (e.g. assure people that their GP won't see what they say)

2. Feedback of adherence

Apps can give you feedback on your results using different visual formats (e.g. table, graph, list, monthly calendar – show examples)

Prompts:

- Which would you prefer? And why?
- Total % score – helpful?
- Would you use this feature?
- What information do you want to see in the 'feedback'?
- What other methods could the app use to feedback reports?
- Would you find weekly or monthly feedback more helpful? Why?

For peer review only - http://bmjopen.bmj.com/site/about/guidelines.xhtml

3. Sensing data

- Now we would like to hear your thoughts on ‘sensing technology’.
- Do you know much about the sensors in our smartphones?

- *[Facilitator explains sensors]*
- *All smartphones have sensors built into the device, which can gather data on how the phone is being used, such as movement and light (e.g. detect if it’s face up or down).*
- *A simple example is when you make a call, and hold the phone to your ear, do you notice that the touchscreen automatically switches off, to stop you pressing buttons with your cheek. This happens because a sensor within the phone can detect when an object is close to its screen.*
- *Another example is how some phones have an inbuilt pedometer which can count the number of steps walked each day, by detecting the ‘walking’ movement of your phone.*

- *[Explain sensors re tailoring the App]*
- *Sensing technology is a novel way to personalise apps to your everyday behaviour.*
- *E.g. with this app, we can use sensors to personalise the messages sent to you, based on information collected from sensors in your phone.*
- *Provide examples of data that could be collected by the app*
- *Location data.*
  - *Detected either by WiFi (e.g. your home WiFi or a café WiFi) or GPS (detecting the location of the phone, like when we use Google Maps and satnavs).*
  - *The app could use this to send you messages at appropriate times, e.g. only when you’re at home.*
- *Movement of phone or pedometer*
  - *e.g. App will detect movement of the phone (running/walking) and will delay a reminder until you’ve finished*

- How would you feel about an app collecting this data?
  - Would it put you off using the app?
- Is there anything that we could do to encourage you to like/change your mind about this feature?
- Is there anything you don’t like about this feature?
- What questions would you have about this? (this will help us to inform the leaflet for ppts)
- What information should we provide for users? How detailed? How? (e.g. website/leaflet)

Close

For peer review only

For peer review only - http://bmjopen.bmj.com/site/about/guidelines.xhtml

Supplementary file 3

Draft outline of a 5-minute face-to-face consultation (VBI)

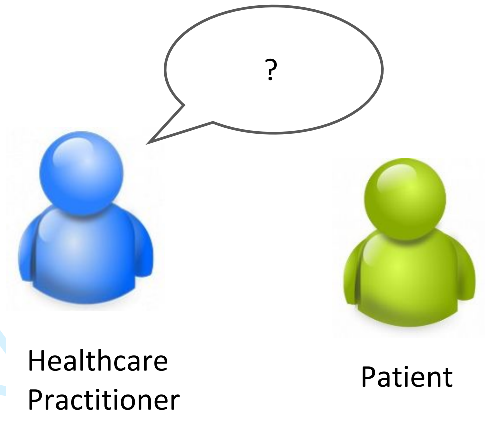

For peer review only

- Start the consultation with de-stigmatising medication non-adherence – forgetting to take tablets and/or having concerns is common.
- Support is available. Introduce the digital support (SMS texts or mobile phone app) as a (fun?) way to help patients take them – perhaps share personal experience with using it, demonstrate a key feature of the app/ text-messaging support.
- Complete short online questionnaire on behalf of patient:
    - Input basic information (patient name, age, mobile phone number)
    - Choose from drop down list of medications that patient is prescribed (tablet name, dose, frequency)
    - Ask patients to give their main reasons for not taking their medication (e.g. choose top 3 reasons from a list, or rank from most important to least important).
- Generate a code for patients to download the app/start the support. E.g. QR code, link to website for patients to download app.
- Hand out pamphlet with further information.
- Endorse the patients' use of digital support.
- Record in the system for follow-up purposes.

For peer review only - http://bmjopen.bmj.com/site/about/guidelines.xhtml

Supplementary file 4: Major themes, sub themes and illustrative quotes

**Theme**	**Sub theme**	**Example participant quote**
**Reasons for non-adherence**	*Non intentional non-adherence (NINA)*<br>Forget to take medication and/or reorder prescription	If I don't put the blister pack back and take the tablets, sometimes I can't remember if I've taken it or not [P03, male, 60s]<br><br>I just totally forgot to take my medication this morning. I just had a cup of tea and a piece of toast. I have to admit I was in a hurry today, I'm sorry, I didn't take the BP [medication]. [P06, female, 60s]<br><br>Usually it's that they haven't had a chance to put their prescription in, so then they tend to run low. [HCP 07, Practice Nurse, female]
	Intentional non-adherence (INA)<br>Unpleasant side effects; general reluctance to take medication	Sometimes I'm tempted to, and if I'm doing some sporting activity, I will be less inclined to take them. They seem to reduce my performance […] perhaps take it after I've indulged in the sport. [P04, male, 40s]<br><br>Often patients will say that they felt fine before they took their blood pressure medicine and now they feel rotten. [HCP 06, Practice Nurse, female]<br><br>Especially with metformin, because it can often have some unpleasant side-effects, and very often people will stop taking it because of this, but they don't actually report that fact to us. [HCP 10, Practice Nurse, female]<br><br>You know, I'm sure there's a lot of natural stuff out there that could help me you know. I don't know what effect it's having inside. That's the trouble, you know…what, in fact, it's doing. [P02, male, 60s]<br><br>I just hate myself for having to take them… I just feel that there must be a natural way… I just feel that, you know, lifestyle choices would be a better way than taking tablets, and that's why I hate myself for having to do it. [P03, male, 60s]

For peer review only - http://bmjopen.bmj.com/site/about/guidelines.xhtml

**Recommendations for intervention content**	Simple reminders to take medication and to reorder prescription (NINA)	It is good about the reminder because I sometimes say to my husband, er, would you remind me and he doesn't because he's worse than I am. [P05, female, 70s]<br><br>I think that would be quite good if it could tell you when you last got your prescription and when your next prescription's due and send a reminder maybe a few days before to say. [FG3, male]<br><br>Do you know what would be handy, is the reminder to tell you when you're gonna run out. That would be good for me. [FG1, male]
	Messages containing information about medication: consequences of non-adherence (targeting INA) acceptable to HCPs but not to patients; preference for advice on how to follow up a missed dose	I guess they need a more educational approach...that's the kind of need to explore why they are choosing not to take it. Do they understand the consequences of not taking it? So the nature of the messaging might be quite sort of shock, 'if you do not take your aspirin you are at risk of having a heart attack', or something like that. (HCP 02, GP, male)<br><br>It's like preaching, and I don't need my phone to be beeping at me in order to preach a message. [FG4, female]

For peer review only - http://bmjopen.bmj.com/site/about/guidelines.xhtml

		I don't know whether I could...whether it's okay to do a catch-up, like if I missed my Ramipril in the morning, can I take that and the Indapamide at night? Or would that be too high a dose in one go? So, you know, maybe that needs to be addressed in the texts [P03, male, 60s]<br><br>Other days I've forgotten completely and then I get home and I think, "Shall I take 'em now or shall I wait 'til tomorrow now?" I've done that once or twice. (M)<br>That is a problem, whether you can take it if you've forgotten. (F)<br>I want to know precisely, "Well, will that affect it big or a lot or not a lot or...? And is taking them all together more important than the hour?" […] It's the occasional expert knowledge that motivates people to stick with an app isn't it? […] It's the fact that if you could press a button and say, "What should I do now 'cos I've forgotten 'em?" might be just a great help. (M) [FG3]
	Feedback on adherence levels – simple statistics preferred	That could be a graph, couldn't it? Or a percentage. You know, 'well done', you know, 'you're ninety-five per cent this month'. [P03, male, 60s]<br><br>Even a weekly graph might be quite nice. Because, then you can see that, actually, when I started a week ago, I was taking fifty per cent…and now I'm going up, and you know, just a visual kind of thing for them. And, actually, now I'm on a hundred per cent, and next week I'm hundred per cent. [HCP 01, Practice Pharmacist, female]<br><br>For me, I think the answer probably is yes actually, because actually that really would be a... you know, a wake-up call, thinking, "God, [participant name], do you realise that's two days this week?" I mean, okay, I should be able to tell what I had but, you know –. [FG4, male]
	Signpost patients to contact their HCP	If you are having side-effects, please contact somebody, rather than just ignore it…. It could be it's the pharmacist, or the Practice Pharmacist rather than the community one. It's just about improving interactions with us, the pharmacist, reporting side-effects. [HCP 06, Practice Nurse, female]

For peer review only - http://bmjopen.bmj.com/site/about/guidelines.xhtml

**Tailoring the digital intervention**	Highly tailored intervention facilitates engagement with SMS messages and smartphone app	It's about tailoring the process to the patients' needs….What suits one is not going to suit the next twenty people. [HCP 06, Practice Nurse, female]<br><br>It would be nice to be a bit personalised, it makes you feel a bit special [FG1, male]<br><br>You can default [medication reminders] to all the days of the week being the same but it might be nice to be able to specify different times of the day if you really wanted to. [FG3, male]<br><br>I mean, could users perhaps make their own decision which, which [reminder message] version they want to have appear? [FG4, female]<br><br>How much margin is there to vary from person to person? So if I said I wanted the full works I could have them but perhaps somebody that didn't? [FG1, male]
	Barrier to engagement – if intervention is overcomplicated or difficult to use	A hundred ideas that you get but if it's not simple then, the app in the end—From personal experience it won't work, it needs to be simple for people to buy into it. [FG2, male]<br><br>But I think it has to be really simple 'cos, you know, the more you're delving into this, it's... it can get complicated, can't it? So people are just gonna want something easy. [FG3, female]
**Acceptability of sensing technology**	Facilitator – Potential for sensing technology to enhance user experience; flexible opt-in/out options	Another time that I might forget to take medication is if I've gone somewhere just overnight ... I might just forget to put it in and I think, oh, well, nothing I can do, I'll have to wait 24 hours or whatever, but if you put in your diary on your phone that you were going to then [the app] might say, "don't forget to pack your pills". [FG2, male]<br><br>I would be happy with the sensing and respond accordingly because I think we could fine tune some of it ourselves couldn't we? If it was personalised. [FG2, female]

For peer review only - http://bmjopen.bmj.com/site/about/guidelines.xhtml

		But could people have an off button for that if they wanted, for that bit of the menu? [FG2, female]
	Barrier to acceptability – concerns about confidentiality of data; requests for information about data use	It's all about how that information is dissembled, isn't it, and so long as that information is within reason locked away somewhere [FG4, male]<br><br>I would definitely want to know you weren't passing my data onto anyone else, either for medical or marketing purposes [FG2, female]<br><br>I'd feel much happier using one that wasn't purely commercial as well. [FG2, female]
**Attitudes towards a VBI (HCP only)**	Barriers and facilitators to implementation: must be straightforward to incorporate and deliver within primary care; five minute time limit potentially unfeasible; sufficient training in delivery of VBI	It would have to be something that's very accessible on your desktop or as part of integrated into the clinical system. [HCP 03, Practice Nurse, female]<br><br>I think this is quite a straightforward process, as I say, because a lot of this we should be doing already and then it's the add on bit at the end. [HCP 06, Practice Nurse, female]<br><br>I don't think it's going to end at five minutes. I think it's probably going to go a bit more than that in some cases [HCP 05, Healthcare Assistant, female]<br><br>I'm quite chatty so they will probably just chat with me, 'cause I make them chat, but it could be easily done in five minutes if you were concise and just got on with it. HCP 08, Practice Nurse, female]<br><br>It would be really good that we've had the training on how to use the app, how to use the questionnaire… It might be useful even just to show us how you might do a consultation. You know, sometimes visually seeing something can be… Even just a video. [HCP 01, Practice Pharmacist, female]<br><br>Quite often what works well is just an almost practical session of: "This is what we're proposing. This is how you do the face to face consultation. This is what

For peer review only - http://bmjopen.bmj.com/site/about/guidelines.xhtml

		the apps look like" and then, you know, it then develops discussion. [HCP 06, Practice Nurse, female]<br><br>The more you do and the easier would become and you get more slick at it, wouldn't you… What we would need is a little bit more information on the type of app and text that you are going to try and set up. And maybe have a dummy run and see how comfortable we felt with it and if there was anything we could do to make it slightly more slicker and more professional… A video clip or something of somebody doing it so we would know how to approach it and address it and stuff like that, will probably be the best thing. [HCP 09, Practice Nurse, female]
	Recommendations for VBI: importance of HCP/patient relationship; understand the root cause of non-adherence and address patient concerns	If we have a very judgmental approach to care then we actually lose the patient's confidence and their respect and actually they won't come back at all. So that is just so important.[HCP 06, Practice Nurse, female]<br><br>You can get through if you put it in the right wording. I do find, I've been doing it for quite a few years now and I find I have got a good, you know, I'm not blowing my own trumpet, but I have got quite a good rapport with my patients [HCP 04, Healthcare Assistant, female]<br><br>I just try to keep it open and honest, and sort of gain their trust so they actually tell me why [they aren't taking their medications]. Most patients, if they know you or if they've dealt with you before, are quite happy to share… If they, um, don't think that they're going to get into trouble [laughs] for not taking it. [HCP 11, Practice Pharmacist, female]<br><br>It's about sort of exploring why, what their understanding is of the medications that they are taking and trying to get an understanding of whether they recognise the importance of compliance. So it's kind of just exploring in a deeper way as to why they've not been taking. [HCP 02, GP, male]<br><br>I tend to just try and get to the bottom of [non-adherence]… It's unpicking what the problems are and what their perception is and sometimes it's quite

For peer review only - http://bmjopen.bmj.com/site/about/guidelines.xhtml

		misguided and it's different from one person to another. [HCP 03, Practice Nurse, female]<br><br>The reasons why patients don't take their medication we should really be exploring in any case… our role in the face to face is actually we can explore some of these things a bit easier. [HCP 06, Practice Nurse, female]

Note: HCP = healthcare practitioner

For peer review only

For peer review only - http://bmjopen.bmj.com/site/about/guidelines.xhtml

BMJ Open

Patient and practitioner views on a combined face-to-face and digital intervention to support medication adherence in hypertension: a qualitative study within primary care

Journal:	*BMJ Open*
Manuscript ID	bmjopen-2021-053183.R1
Article Type:	Original research
Date Submitted by the Author:	30-Sep-2021
Complete List of Authors:	Van Emmenis, Miranda; University of Cambridge, Public Health and Primary Care<br>JAMISON, JAMES; University of Cambridge, Public Health and Primary Care<br>Kassavou, Aikaterini; University of Cambridge, Public Health and Primary Care<br>Hardeman, Wendy; University of East Anglia, School of Health Sciences<br>Naughton, Felix; University of East Anglia Faculty of Medicine and Health Sciences, School of Health Sciences<br>A'Court, Charlotte; University of Cambridge, Public Health and Primary Care<br>Sutton, Stephen; University of Cambridge, Public Health and Primary Care<br>Eborall, Helen; The University of Edinburgh, Usher Institute
**Primary Subject Heading**:	Public health
Secondary Subject Heading:	Qualitative research
Keywords:	QUALITATIVE RESEARCH, Hypertension < CARDIOLOGY, PRIMARY CARE, PUBLIC HEALTH

SCHOLARONE™
Manuscripts

I, the Submitting Author has the right to grant and does grant on behalf of all authors of the Work (as defined in the below author licence), an exclusive licence and/or a non-exclusive licence for contributions from authors who are: i) UK Crown employees; ii) where BMJ has agreed a CC-BY licence shall apply, and/or iii) in accordance with the terms applicable for US Federal Government officers or employees acting as part of their official duties; on a worldwide, perpetual, irrevocable, royalty-free basis to BMJ Publishing Group Ltd ("BMJ") its licensees and where the relevant Journal is co-owned by BMJ to the co-owners of the Journal, to publish the Work in this journal and any other BMJ products and to exploit all rights, as set out in our licence.

The Submitting Author accepts and understands that any supply made under these terms is made by BMJ to the Submitting Author unless you are acting as an employee on behalf of your employer or a postgraduate student of an affiliated institution which is paying any applicable article publishing charge ("APC") for Open Access articles. Where the Submitting Author wishes to make the Work available on an Open Access basis (and intends to pay the relevant APC), the terms of reuse of such Open Access shall be governed by a Creative Commons licence – details of these licences and which Creative Commons licence will apply to this Work are set out in our licence referred to above.

Other than as permitted in any relevant BMJ Author's Self Archiving Policies, I confirm this Work has not been accepted for publication elsewhere, is not being considered for publication elsewhere and does not duplicate material already published. I confirm all authors consent to publication of this Work and authorise the granting of this licence.

For peer review only

ORIGINAL RESEARCH

Patient and practitioner views on a combined face-to-face and digital intervention to support medication adherence in hypertension: a qualitative study within primary care

Miranda Van Emmenis[1], James Jamison[1], Aikaterini Kassavou[1], Wendy Hardeman[2], Felix Naughton[2], Charlotte A' Court[1], Stephen Sutton[1], Helen Eborall[3]

[1] Behavioural Science Group, University of Cambridge, United Kingdom

[2] School of Health Sciences, University of East Anglia, United Kingdom

[3] Usher Institute, University of Edinburgh, United Kingdom

Correspondence:

Miranda Van Emmenis

Behavioural Science Group, Primary Care Unit, East Forvie Building, Forvie Site, Robinson Way, Cambridge, CB2 0SR

Tel 01223 330524

Email mv404@medschl.cam.ac.uk

Word count: 5621

For peer review only

Abstract

Objectives: To explore patients' and healthcare practitioners' views about non-adherence to hypertension medication and potential content of a combined very brief face-to-face discussion (VBI) and digital intervention (DI).

Methods: A qualitative study (N=31): interviews with patients with hypertension (n=6) and healthcare practitioners (HCPs; n=11) and four focus groups with patients with hypertension (n=14). Participants were recruited through general practices in Eastern England and London. Topic guides explored reasons for medication non-adherence and attitudes towards a potential intervention to support adherence. Stimuli to facilitate discussion included example SMS messages and smartphone app features, including mobile sensing. Analysis was informed methodologically by the constant comparative approach and theoretically by Perceptions and Practicalities Approach (PAPA).

Results: Participants' overarching explanations for non-adherence were non-intentional (forgetting) and intentional (concerns about side-effects, reluctance to medicate). These underpinned their views on intervention components: messages that targeted forgetting medication or obtaining prescriptions were considered more useful than messages providing information on consequences of non-adherence. Tailoring the DI to the individuals' needs, regarding timing and number of messages, was considered important for user engagement. Patients wanted control over the DI and information about data use associated with any location sensing. While the DI was considered limited in its potential to address intentional non-adherence, HCPs saw the potential for a VBI in addressing this gap, if conducted in a non-judgemental manner. Incorporating a VBI into routine primary care was considered feasible,

For peer review only

provided it complemented existing GP practice software and HCPs received sufficient training.

Conclusions:

A combined VBI-DI can potentially address intentional and non-intentional reasons for non-adherence to hypertension medication. For optimal engagement, recommendations from this work include a VBI conducted in a non-judgmental manner and focusing on non-intentional factors, followed by a DI that is easy-to-use, highly tailored, and with provision of data privacy details about any sensing technology used.

Keywords: medication adherence, qualitative research, high blood pressure, digital intervention, healthcare professional, very brief face-to-face intervention

For peer review only

Strengths and limitations of the study

- To our knowledge, this is among the first qualitative studies to gather patient views on the use of sensing technology such as Wi-Fi or GPS within a smartphone app to support medication adherence.
- The study sought the views of a range of healthcare practitioners on incorporating a very brief intervention for medication adherence into a primary care consultation, a topic not previously explored in-depth.
- The use of stimulus materials provided detailed and focused responses for specific intervention components such as feedback on adherence and content of messages.
- While the sample size was small, the depth and focus of insights gained are sufficiently useful in informing the development and refinement of intervention components.

Introduction

Medication adherence, defined as the level to which an individual takes medication as intended by their healthcare prescriber, is a worldwide public health concern.[1] Non-adherence to blood pressure lowering medication is estimated at 41%, which is relatively high compared with many other medications.[2, 3] This is associated with increased risk of cardiovascular disease related morbidity and mortality.[4, 5] Given that high blood pressure is responsible for nearly 20% of deaths worldwide, non-adherence to antihypertensive treatment is a global health concern.[6]

For peer review only - http://bmjopen.bmj.com/site/about/guidelines.xhtml

Previous research into medication non-adherence has documented its complexity and multifaceted nature.[7, 8] Two broad categories within this are (i) non-intentional non-adherence, a passive process due to factors not directly within an individual's control, such as memory or access difficulties,[8, 9] and (ii) intentional non-adherence, a more deliberate action whereby an individual makes a conscious decision not to take their medication due to their perceptions about or experiences with their medication or condition.[9]

The multifaceted nature of non-adherence presents a challenge to those developing interventions to support adherence; for example, determining which factors to target, while balancing feasibility of delivery with likely effectiveness. Digital interventions (DIs) such as SMS text messaging or smartphone applications (apps) offer interactive, low cost and scalable methods of providing support to individuals for whom medication adherence is a challenge. DIs are particularly suitable given the increasing use of these by people across the age groups for day-to-day tasks, such as apps for alarm clocks, calendars and shopping lists.[10] In addition, the benefit of DIs over traditional face-to-face approaches is that they do not require lengthy consultations with healthcare practitioners, whose time is scarce, especially during the current Covid pandemic. [11, 12]

Evidence for the effectiveness of DIs in improving medication adherence is promising (e.g. see Thakkar et al [13]). In a recent systematic review of app-based interventions, patients using a smartphone app to support medication adherence for various health conditions were twice as likely to report taking their medications than those receiving usual care.[14] Specific to hypertension, DIs such as SMS messages, smartphone apps, email and Bluetooth blood pressure monitors have

For peer review only

been shown to improve medication adherence and lower both diastolic and systolic blood pressure.[15, 16]

Incorporating sensing technology into smartphone apps potentially expands the scope of DIs further. Passive smartphone sensors can collect user location data via GPS or Wi-Fi to enable the delivery of real-time support [17], which is of particular relevance given that non-intentional non-adherence is strongly influenced by a person’s physical environment.[18] Smartphone sensing technology has shown success in DIs across the domain of health and well-being,[19] but user acceptability of such technology in a smartphone app to support medication adherence is largely unknown.

While user acceptability is key to use of a DI, potential users first need to install and engage with the DI for it to provide benefit. Primary care professionals, such as practice nurses or community pharmacists are ideally placed for encouraging uptake of DIs for medication adherence, for example during a medication review or at the point of prescription collection. However, evidence is limited on how healthcare professionals can best promote the uptake of DIs for medication adherence. The acceptability of combining a DI with a very brief face-to-face intervention delivered by a healthcare professional to support medication adherence has also not been widely explored.

This study aimed to explore patients’ and healthcare practitioners’ views on i) non-adherence to hypertension medication and ii) a complex intervention designed to support medication adherence. Initial ideas for the intervention consisted of a very brief face-to-face discussion with a primary care provider, followed by ongoing support via a DI (SMS messages or smartphone app). Feedback from participants

For peer review only

included preferred content of the intervention and factors likely to influence engagement.

Methods

This study is reported in line with the consolidated criteria for reporting qualitative research studies checklist (COREQ)[20], see supplementary file 1.

Design

We undertook a qualitative study using semi-structured interviews followed by focus groups.

Recruitment and sampling

Patients were recruited for interviews from primary care practices based in the East of England (n=3) and East London (n=1). Practices were identified with the help of the Clinical Research Network, an organisation which supports the delivery of research within NHS practices in England. Patients were eligible to participate if they were: 1) prescribed at least one antihypertensive medication for at least the previous three months 2) deemed non-adherent according to GP practice records, with a blood pressure reading of over 140/90 mmHg and/or gaps in filling repeat prescriptions in the previous three months and 3) used either SMS or smartphone apps. The practice administrator at each site generated a list of prospective participants that met criteria 1 and 2, which was screened by a GP or Practice Nurse. Eligible patients received a study pack from their GP practice in the post consisting of an invitation letter and participant information sheet. Posters highlighting the study were also displayed in the GP practices. Patients interested in

For peer review only - http://bmjopen.bmj.com/site/about/guidelines.xhtml

taking part were invited to contact the researcher (MVE) via telephone or email; at which point the researcher checked that all three eligibility criteria were met before scheduling an interview.

A convenience sample of healthcare practitioners were recruited from the four GP practices taking part in this study. Healthcare practitioners were eligible to be interviewed if they were involved in the care of patients with hypertension e.g. through medication reviews (conducted by a GP, Practice Nurse or Practice Pharmacist) or blood pressure checks and/or health assessments (conducted by a Healthcare Assistant). The researcher invited healthcare practitioners to participate during the face-to-face study set-up meeting where they were given a study information pack. The researcher contacted the healthcare practitioners one week later to check willingness to participate and to schedule interviews for those who were interested.

Recruitment for focus groups followed that of the patient interviews. To address the low response from eligible patients, the eligibility criteria was widened to include patients prescribed medication for type 2 diabetes, as research indicates similar rates of medication non-adherence and barriers to adherence as for hypertension.[21, 22] The eligibility criteria was also narrowed to ensure that participants were familiar with using smartphone apps (i.e. SMS alone was not sufficient). The decision to cease individual interviews and switch to focus groups with patients was due to preliminary analysis from the interviews adding little new information to findings from previous research, and our experience of the usefulness of focus groups to gain feedback on the format, content and structure of DIs. [23–25]

Data collection

For peer review only - http://bmjopen.bmj.com/site/about/guidelines.xhtml

Patient interviews were conducted by one researcher (MVE) at patients' home, workplace or local library. Healthcare practitioner interviews were conducted at their place of work by the same researcher (MVE). Focus groups were conducted at community centres local to the patients' general practice and moderated by two researchers (MVE and JJ).

Interviews and focus groups were guided by flexible topic guides [26] developed by the research team, drawing on the Perceptions and Practicalities Approach (PAPA) framework [18] and previous research experience in both the topic area and intervention development. Topic guides were reviewed by Patient and Public Involvement (PPI) representatives to ensure the questions were easy to understand and appropriate for the study objectives. Broadly, interview topics included: reasons for medication non-adherence, current practice of HCPs during medication-related consultations, and views on a potential SMS text message or smartphone app intervention that could support adherence. Example intervention content included medication reminders, advice and support messages, and feedback on adherence. See supplementary file 2 for the topic guides and example DI content. HCPs were shown an example protocol for a very brief face-to-face discussion or 'VBI' to generate discussion (see supplementary file 3). Components of the VBI included: introducing the digital support to the patient and inputting basic patient information via an online questionnaire to generate the tailored digital support.

Focus groups followed similar topic guides to the interviews, focusing on attitudes towards smartphone apps in particular, including the acceptability of sensing technology such as location sensing. To prompt discussion and gain feedback, both interview and focus group participants viewed stimulus materials of

For peer review only

example intervention messages, including medication reminders, and smartphone app features, including graphs and images (see Figure 1 for examples).

Written informed consent was taken in person by the researcher immediately prior to the interviews and focus groups commencing. All patients received a £20 voucher for taking part. Interviews and focus groups were audio-recorded and professionally transcribed verbatim. Interviews lasted on average 47 minutes and focus groups 1 hour and 28 minutes.

Data Analysis

Analysis was informed methodologically by the constant comparative approach[27] and theoretically by the Perceptions and Practicalities Approach (PAPA), which incorporates and acknowledges the blurring of and distinction between intentional and non-intentional non-adherence.[18] Interview transcripts were read and re-read to aid familiarisation and identify preliminary themes; these broad descriptive themes were formed into an initial coding framework related to barriers and facilitators to medication adherence and a potential intervention. Each transcript was then coded systematically (MVE) using NVivo qualitative data-indexing software (version 12; QSR International) and the coding framework was refined throughout the process. The process was repeated for focus group transcripts; the coding framework was further expanded and refined, given the additional topics explored in the focus groups. A sample of interview and focus group transcripts were independently coded by a second researcher (JJ) to confirm and strengthen the validity of findings. Meetings between the research team (MVE, JJ, HE) facilitated data analysis including discussion of themes, sub-themes and the interrelationships.

For peer review only - http://bmjopen.bmj.com/site/about/guidelines.xhtml

Patient and public involvement

All study materials (participant information sheet, invitation letter, study poster, consent form, topic guides and stimulus materials) were reviewed by representatives from the Cambridge University Hospitals Patient and Public Involvement (PPI) panel. We made a number of changes to the study materials as a result of PPI input: adjusted the language to make the documents more accessible and ensured interview questions were sensitively worded and easy to understand from a patient perspective. PPI representative Jennifer Bostock provided input throughout the study and reviewed and commented on this manuscript.

Ethical approval

Ethical approval for this study was obtained from the West Midlands – Solihull Research Ethics Committee, as part of NHS Health Research Authority approvals (Reference: 18/WM/0050).

Results

Of the 126 eligible patients prescribed medication for hypertension who were sent an invitation, six were interviewed. All 11 healthcare practitioners approached by the researcher were deemed eligible and agreed to take part. Of the 218 patients prescribed medication for hypertension and/or type 2 diabetes who were then sent an invitation to a focus group, 14 participated (four focus groups with 3-5 patients per group). Recruitment of participants to focus groups continued until no new themes were emerging in relation to the specific topics covered.

For peer review only

Patient participant characteristics are reported in Table 1. Their mean age was 62.7 years (range 47 – 79 years), 60% identified as male and 85% as White British. Eighty percent of patients reported using both SMS and smartphone apps, with the remaining 20% using SMS text messages only. All patients self-reported having occasionally missed or skipped their medication in the previous 3 months. HCP participant characteristics are reported in Table 2; six Practice Nurses, two Healthcare Assistants, two Practice Pharmacists, one GP. Participants were recruited from four GP practices based in urban (n=3) and rural (n=1) locations. GP practice Index of Multiple Deprivation (IMD) scores, a measure of relative socio-economic status in England based on postcode, ranged from ‘least deprived’ (n=2), to ‘less deprived’ (n=1) and ‘more deprived’ (n=1), see Table 2.

Table 1 Participant characteristics (patients)

Characteristics	(n)	%
**Gender**		
Female	8	40
Male	12	60
**Age (years)**		
41-50	2	10
51-60	4	20
61-70	11	55
71-80	3	15
**Ethnicity**		
Asian or Asian British – Indian	1	5
Asian or Asian British – Pakistani	1	5
Black or Black British – Caribbean	1	5
White British	17	85
**Phone use**		
SMS only	4	20
SMS and smartphone app	16	80
**Data collection method**		
Semi-structured interview	6	30

For peer review only

Focus group	14	70

Note: n=20

Table 2 Participant characteristics (healthcare practitioners) and GP practice demographics

Characteristics	(n)	%
**Healthcare practitioners**		
**Job role**		
General Practitioner	1	9
Healthcare Assistant	2	18
Practice Nurse	6	55
Practice Pharmacist	2	18
**Gender**		
Female	10	91
Male	1	9
**Years practising**		
≤10	5	46
11-20	2	18
21-30	2	18
≥31	2	18
**GP practices**		
**GP practice location**		
Urban	3	75
Rural	1	25
**GP practice IMD quintile**		
First quintile (least deprived)	2	50
Second quintile (less deprived)	1	25
Fourth quintile (more deprived)	1	25

Note: Healthcare practitioner n=11, GP practice n=4, IMD = Index of Multiple Deprivation, which ranks every small area in England from 1 ('most deprived area') to 32,844 ('least deprived'). IMD scores of the 4 GP practices in this study: 9924, 25270, 26871, 30807.

For peer review only - http://bmjopen.bmj.com/site/about/guidelines.xhtml

For peer review only

To present the findings, we broadly categorise the key themes identified into the following categories: reasons for non-adherence, recommendations for message content, tailoring the digital intervention, acceptability of sensing technology, and attitudes towards a VBI. We provide illustrative quotes below. See supplementary file 4 for additional quotes from participants. For reference, 'digital intervention' (DI) refers to both SMS text messages and smartphone app, as the same intervention messages can be delivered using both formats.

Reasons for non-adherence

Participants provided two key explanations of non-adherence to antihypertensive medication. First, for *non-intentional* non-adherence, forgetting was the most common reported reason and was mentioned in three ways: forgetting to take medication, forgetting whether or not medication had been taken and forgetting to re-order the prescription in time.

> *Sometimes you can't remember whether you have taken them already. And that can be problematic, so if someone asks you, you think, 'well, I don't know, maybe I have, maybe that was yesterday.' [P04, male, 40s]*

Second, in terms of *intentional* non-adherence, the experience or anticipation of side effects was a reason given for skipping, altering or delaying medication, as was the general reluctance to be reliant on medication.

> *I wish I could live without medication, I hate pumping my body with drugs. Sometimes I wonder, 'what side effects am I gonna have with this? Is it really benefitting me?' [P06, female, 60s]*

> *A lot of patients […have said], 'yes, the doctor has changed my medication, but they make me go funny, so I'm just going to take half or I'll just crush that and just take half instead of the two.' [HCP 05, Healthcare Assistant, female]*

These overarching explanations were apparent when participants discussed the merits of a digital intervention to improve adherence, as presented in the following sections. We begin with participants' views about a DI's messaging content, followed

For peer review only

by tailoring and then sensing technology; the final section considers the role of the VBI component.

Recommendations for message content

Simple reminder messages were perceived as useful for both taking medication and re-ordering prescriptions.

> *Even if I'm in a hurry, [when] I receive this reminder I [would] realise the importance. I think if I keep getting messages that would be very effective and definitely help me. Even if I'm tired and it would make me […] I'd force myself to get up and go and take the medication. [P06, female, 60s]*

> *It would be useful, if you're running out of tablets, to have some way of automatically reordering or a reminder to do that. So it's reminding you to take your tablets, and also when you're running low. [P04, male, 40s]*

Information-giving messages were only perceived as helpful by participants if providing advice when medication had been missed, for example the safest way to 'catch up' on a missed dose.

> *There ought to be a button of 'I've forgotten them 'til now, which bits should I take?' That could be useful. [FG3, male]*

While HCP-participants recommended messages about the benefits of medication or the consequences of non-adherence, patient-participants considered these unhelpful and unnecessary, particularly if lack of knowledge was not a barrier to adherence.

> *I know what the risk is [from not taking my medication]. I don't feel that I want it repeated, no. [P03, male, 60s]*

There was, however, some recognition that newly-diagnosed patients may find such information motivating:

> *If you're new to taking blood pressure tablets [information on consequences of non-adherence] would be good. I mean, us experienced people who've taken them for years most probably don't need reminding that if you don't take it, something serious is gonna happen to you. [FG3, male]*

For peer review only - http://bmjopen.bmj.com/site/about/guidelines.xhtml

The idea of receiving feedback on one's adherence, generated from self-report via SMS message or App, in a message of encouragement (e.g. *'Well done!')* was viewed as unnecessary. Participants were more receptive to schematic feedback in the form of a graph, score or percentage.

> *Some people might need that encouragement, but then again, it sounds a bit patronising to some people, doesn't it [laughs]? […] I think the percentage thing would give people pride, you know, 'oh, I've reached 100% [of taking my medication] this month, I feel really good about that'. Once a month I'd like to know what my score was for the month. I think that would probably be enough incentive for me personally. [P03, male, 60s]*

Regular smartphone users suggested that feedback in the form of a monthly calendar highlighting 'missed medication' days, could be useful for spotting a pattern and identifying the circumstances of those days that contributed to a missed dose. Moreover, participants suggested the potential for this to facilitate discussion with a healthcare professional too:

> *A: I think [the app] would also be good to take, when you have a medication review, to take to your GP so he or she can see what's going on as well.*
> *B: Yeah, help fix it by doing something, yeah*
> *[FG3, male (A, B)]*

Tailoring the digital intervention

Participants commented how they would be more likely to use, and continue to use, the DI if the messages were tailored to their preferences and their individual medication regime, in terms of frequency and timing of doses:

> *Some people are on medication once a day, twice a day, three, four. Could the app be tailor-made for the individual? And remind us accordingly? [FG2, female]*

> *A: That's why [the intervention] should be tailor-made for the individual patient. I think it's going to be critical really. Rather than a generic –*
> *B: And have options, yeah.*
> *A: Because if it's a generic app and it doesn't suit some people they won't use it or they won't respond to it. [FG1, male (A,B)]*

For peer review only - http://bmjopen.bmj.com/site/about/guidelines.xhtml

Participants noted the importance of the DI including *all* their prescribed medications, i.e. not just the hypertension ones.

> *I think it would need to be somewhat of a select or deselect, you know, 'take all' but you can un-tick the ones that you're not taking now. [FG4, female]*

> *A: I would do it as all one. Even if you're doing it principally motivated by blood pressure, in the sense it's, you're trying to remind us to take pills in general, aren't you, so you have to somehow-*
> *B: Yes, I think you want all of them there. [FG3, male (A), female (B)]*

To ensure that tailoring meets patients' preferences and medication regime, and the changes over time, participants highlighted the importance of patients having control over the DI's settings. For example, being able to change timings of reminders and adding in short term medication.

> *A: I think I'd like to put my own [medications] in. And then when you have a "short course" [of medication] as we say, I'll add that in as well. I'd rather be in charge of putting it in.*
> *B: Especially as some you have to have on an empty stomach, don't you?*
> *A: Yeah, so you could fiddle with your timings for that one. [FG2, female (A), male (B)]*

> *It's gotta be a dynamic thing. Medications change, dosages change, things get stopped, times may change, so I probably would see as an app which patients would be free to add and subtract. [HCP 02, GP, male]*

A 'snooze' function (similar to an alarm snooze) was well-received by participants, provided users could set their own parameters, e.g., length of snooze duration and maximum number of snoozes.

> *It would be good for me 'cos I'm often not home when I'm supposed to take them, so if you hit the 'snooze' for an hour or whatever you choose it to be, […]and it'll keep reminding me again and I'll take the tablets [when I'm home]. [FG1, male]*

For peer review only

A suggestion for tailoring by adding images of medications into the app raised more problems than benefits; participants pointed out that *"every time you get the medication, the box changes" [FG1]* and it was felt this would create confusion, rather than help.

Acceptability of sensing technology

Participants were initially wary about the incorporation of sensing technology, such as GPS or Wi-Fi to determine location, into an app. They raised concerns about surveillance, typically referred to as "*Big Brother*" *[FG1, FG3]* watching them. Participants were more likely to accept sensing technology if the perceived benefits (such as tailoring medication reminders to their specific schedule and locations) outweighed concerns about data privacy.

> *It would make it impossible to forget 'cos I'd just walk through the door and take 'em. That would be brilliant. [FG1, male]*

Participants requested information to address these concerns, including: who has access to their data beyond the university (in particular, less trusted organisations such as insurance or marketing companies), where data are stored, and what happens in the event of hacking.

> *A: Who are you gonna share this with? That's all I'm worried about […]*
> *B: It could be pretty valuable information for insurance companies to put their premiums up. [FG4, male (A), female (B)]*

> *I think it'd be more reassuring to know it was a medical body behind it or a university body behind it; it gives it some substance and credibility. [FG1, male]*

Participants wanted to retain personal control over the sensing function, with the ability to choose when the app tracks and records their location data as well as the

For peer review only - http://bmjopen.bmj.com/site/about/guidelines.xhtml

ability to opt in/out at any point.

> *I think it would be a case of opt-in because I think some people would think it an invasion of privacy. I mean, personally I think it's a good idea but, you see, some people wouldn't like it. [FG4, female]*

Discussions about sensing technology prompted participants to suggest further ideas for functions of an app. Participants in two focus groups suggested linking the sensing technology with the smartphone calendar, to proactively detect periods when away from home, triggering reminder messages to pack medication or re-order prescriptions.

> *The app ought to be able to detect [that] my calendar says, 'Away for the weekend.' So the app could say, "Ooh look, you're going away for the weekend" it could send me a message or something on the Friday to make sure I pack them. That's almost what I want to be reminded of. [FG3, male]*

Participants emphasised the need for additional features to be optional, recognising that over-complicating the DI risked disengagement from potential users.

> *I suppose it's a case though of getting sufficient ability to customise it against making it just too longwinded and complicated for people to be bothered. [FG4, female]*

> *I'm just trying to think of just the least steps possible for the patient, because just adding more things is going to make them less likely to use these sorts of things… It needs to just be easy for them. [HCP 01, Practice Pharmacist, female]*

Above all, participants emphasised the importance of the DI being user-friendly for the target group, many of whom may be less familiar with smartphones.

> *The caveat I suppose might be that those that tend to have the chronic diseases tend to be the older age group so they may not be so tech savvy. We've got some patients who don't use mobile phones even now. [HCP 02, GP, male]*

Attitudes towards a very brief face-to-face intervention (VBI)

For peer review only - http://bmjopen.bmj.com/site/about/guidelines.xhtml

Patient-participants' discussions about the DI functions largely focused on addressing non-intentional non-adherence - mainly forgetting. On the whole, they were sceptical about a DI's success in addressing intentional non-adherence:

If they're not taking the tablets and they don't wanna take the tablets, why would they sign up for the app? [FG1, male].

HCP-participants suggested including messages encouraging users to contact their healthcare provider if experiencing problems with their medication.

That would be really useful in that if they're stopping it for any reason it needs to come up with a message to say, "Please make an appointment with your GP. There may be alternative medications available which would suit you and you need to make an appointment to discuss that". [HCP 03, Practice Nurse, female]

However, a more promising way of addressing intentional non-adherence was highlighted in relation to the 5-minute VBI component prior to use of the DI. The VBI was presented as a way for HCPs to signpost patients to the DI and discuss medication taking behaviour. HCPs talked positively about how, if done in a non-judgemental way and by an HCP with an established rapport with the patient, this could foster open communication and a more constructive consultation.

That's the important thing, when patients can relate to you and they can see that you're actually not judging them, they do tend to then engage better. [HCP 07, Practice Nurse, female]

A key aspect of encouraging honest communication in the VBI would be acknowledging that it is acceptable to have concerns about being prescribed medication. HCPs recommended asking the patient to talk through these concerns and, if needed, book a follow-up consultation with a prescribing practitioner about changing medication.

Have a discussion with them as to what's been happening, what the issues are, how we can make it easier for them […] 'Is there a problem with it? Are you getting side-effects? Do you find it difficult to take?' And then we can explore some of the issues. What is really important is to sift through what the issues

For peer review only

For peer review only - http://bmjopen.bmj.com/site/about/guidelines.xhtml

are. Our role in the face-to-face is actually we can explore some of these things a bit easier. [HCP 06, Practice Nurse, female]

All HCPs perceived the VBI element as feasible to deliver within primary care, recommending incorporating it alongside a medication review or blood pressure check. HCPs had two key provisos: training to help them deliver the VBI within the tight timing of five minutes, and a "*user-friendly*" template that could be incorporated in existing computer systems for inputting patient data to inform the subsequent DI. HCPs also noted the need for sufficient training in using the DI itself, given their role in encouraging its use in their patients following the VBI.

I think that will be important, that whoever is talking about the app needs to know how it works and how you use it… Because if somebody who is recommending it doesn't know how to use it then you're not gonna buy into it. [HCP 09, Practice Nurse, female]

For peer review only

For peer review only - http://bmjopen.bmj.com/site/about/guidelines.xhtml

Discussion

Summary of main findings

Patients prescribed antihypertensive medication and the healthcare practitioners that care for them, highlighted nonintentional (forgetting) and intentional (side-effects, reluctance to medicate) reasons for their non-adherence. Participants felt a mobile digital intervention (DI) that provided simple medication reminders and feedback messages would help improve adherence. To facilitate engagement with the DI, participants recommended it was tailored to the needs of the individual and their medication regime as well as providing user control over the tailoring and other optional functions. The use of sensing technology within a smartphone app was acceptable to participants provided they received comprehensive information about the associated use and confidentiality of their data.

While the DI was considered limited in its potential to address intentional non-adherence, HCPs saw the potential for a brief face-to-face discussion (or 'VBI') with patients in addressing this gap. Incorporating a VBI into a routine primary care was considered feasible, if it could be integrated into existing practice software systems and if training were provided.

Strengths and limitations of the study

This study was conducted as development work with a target patient group to inform aspects of an intervention as part of a larger research programme.[28] This study draws on relevant theory [8, 9, 18] and is among the first qualitative studies to gather patient views on the use of sensing technology (e.g. Wi-Fi or GPS) in a smartphone app to support medication adherence (see also Kassavou and

For peer review only - http://bmjopen.bmj.com/site/about/guidelines.xhtml

colleagues[29]). Whilst advances in technology can provide additional features to smartphone apps, it is important to assess the intended user group's views of such technology before its implementation.[30]

We gained novel insights from a range of healthcare practitioners on the acceptability and feasibility of incorporating a VBI for medication adherence into a primary care consultation, a topic that has not been previously explored in-depth. Our findings can inform the development and implementation of a medication adherence VBI in primary care. The use of stimulus materials generated discussion in the interviews and focus groups, and provided focused responses for specific hypothetical intervention components.

We acknowledge that this is a small-scale qualitative study, where 85% of the patient sample were White British and 91% of the healthcare practitioner sample were female. As such, the findings may be limited in their application to a patient and healthcare professional population. However, the depth and focus of insights gained provided rich data that were sufficiently useful in informing the development and refinement of intervention components for the wider programme, and to similar interventions.

We experienced challenges with recruiting patients through GP practices, particularly those who were non-adherent to their medication, a group who may be less likely to participate in a study of this nature. For future studies we would recommend widening recruitment methods to include patients not tied to a specific sample of GP practices, e.g. via social media channels or community groups.

Comparisons with existing literature

For peer review only - http://bmjopen.bmj.com/site/about/guidelines.xhtml

The findings echo previous research that has identified the main reasons for non-adherence to cardiovascular-related medication as forgetting and side-effects, [22] [31] as well as the broad categorisation of reasons into intentional and non-intentional.[32, 33] In our study, this distinction was particularly helpful when considering which elements of an intervention were appropriate for targeting these two broad categories.

Participants with lived experience of hypertension saw little value in information-style messages (e.g. about the consequences of non-adherence) in addressing intentional non-adherence. Rather, they suggested that such messages may be most helpful for newly diagnosed patients, a finding which replicates previous research on digital interventions for cardiovascular medication. [34, 35] Similar to previous studies [24], participants expressed concerns about receiving too many messages, suggesting this would influence (dis)engagement with the DI. Participants also emphasised the need for a DI to be as simple and easy-to-use as possible, another common theme in usability studies for medication adherence DIs, whereby difficulties with navigating a website, SMS or smartphone app have presented barriers to usage.[36, 37] A related concern is the potential burden that self-monitoring DIs place on the user, for example, asking patients to self-report their medication taking behaviour within a set timeframe.[38, 39] Our findings support the need for usability testing with the DI target users, which could include assessing any associated burdens or extra responsibilities placed on the user.

Participants in this study raised data privacy concerns about the use of sensing technology within a medication reminder smartphone app. This reflects the discourse around the ethics of mHealth, whereby third parties and insurance companies pose potential threats to the safety of patients' health data collected by

For peer review only

sensors or smartphone apps.[38] These ethical considerations are of particular importance given the rise of mHealth in the healthcare sphere.

Recommendations for an intervention to support medication adherence

The findings from this study have several implications for the development of a DI to support medication adherence. To encourage engagement with an intervention, it needs to be highly tailored to each individual. This includes: the timing and content of reminder messages (to address non-intentional non-adherence) and the content of support messages (for intentional non-adherence), where knowledge and duration of health condition varies between individuals. Furthermore, a key tailoring variable as recommended by HCP-participants was the individual patient's specific barrier(s) to adherence. Tailoring data can be collected using various methods, ideally before the start of the intervention for optimal impact. This could include a short questionnaire, in person or by phone with a practitioner, within a smartphone app, or via a set of SMS messages requiring responses.[24]

It was common for participants in this study to be taking multiple medications per day, and most wanted this to be reflected in the medication reminders. This requires a balance between providing appropriate adherence support without over-complicating the DI or overburdening the user, resulting in reduced intervention engagement.[40]

This study obtained novel insight from patients on the use of passive sensing technology within a medication adherence smartphone app. To increase the acceptability of sensing technology, future apps should explain the benefits that it can provide to the user, such as tailored medication reminders based on real-time

For peer review only - http://bmjopen.bmj.com/site/about/guidelines.xhtml

location, or prompts to pack medication for upcoming holidays detected via calendar syncing. The app must provide a flexible opt in/out option for the collection of sensing data as well as information on how personal data will be used and stored within the app. Lastly, users may be more accepting of a location-sensing app created by a university or charity rather than a commercial company, which follows previous qualitative user testing into GPS-based smartphone apps for behaviour change. [29, 41, 42]

Primary care was viewed as an appropriate setting for HCPs to introduce patients to a DI and pair it with a brief behavioural face-to-face discussion, or ‘VBI’. More specifically, this could address intentional non-adherence by exploring the specific barriers to medication adherence with patients. Using a non-judgemental approach for this, would encourage patients’ openness, which in turn would provide more useful information for tailoring the DI and possibly making adjustments to the patient’s regime as part of the usual care. This supports a body of literature on shared decision-making [43] which has demonstrated an association between an improved patient-professional partnership and medication adherence, for a variety of conditions [44] and for hypertension specifically.[43, 45] Delivering a VBI requires skill, in order to incorporate all elements and within the short timeframe.[46, 47] Our study indicates the importance of incorporating the principles of shared decision-making into VBI training for healthcare professionals.

Conclusion

Overall, patients and healthcare practitioners saw the benefit of receiving medication reminders via SMS message or smartphone app. Intervention developers

For peer review only - http://bmjopen.bmj.com/site/about/guidelines.xhtml

should consider an intervention that is highly tailored to the user, straightforward to use, and addresses data privacy concerns. The use of sensing technology in an adherence support smartphone app was acceptable to patients, therefore future research could investigate the feasibility of incorporating such technology into a smartphone app for adherence. A routine primary care consultation was viewed as an appropriate setting to introduce the DI to patients and discuss medication-taking behaviour with patients, but the feasibility of delivering it as 'very brief' i.e. under five minutes, should be explored further.

Supplementary material

S1 – COREQ checklist

S2 – Topic guides and sample of proposed intervention content

S3 – Example VBI protocol

S4 – Extra participant quotations

Figure 1 (legend)

Example digital intervention content to generate discussion in interviews and focus groups; medication reminder notification, feedback on medication adherence levels (weekly and monthly), SMS support message

Acknowledgements

For peer review only

This study was conducted on behalf of the Programme on Adherence to Medication team (see https://www.phpc.cam.ac.uk/pcu/research/research-projects-list/other-projects/pam/ for team members). We acknowledge and thank the following individuals for contributing to the development of the topic guides and visual prompts for this study: Jagmohan Chauhan and Sandra Servia, Department of Computer Science and Technology, University of Cambridge; Debi Bhattacharya, School of Pharmacy, University of East Anglia.

We thank the patients, healthcare practitioners, practice managers and administrative staff at the 4 GP practices who took part in this study. We are grateful to Patient and Public Involvement (PPI) representatives for reviewing the design and ethics of this study. We thank PPI representative Jennifer Bostock for their input across the whole study and for reviewing the manuscript. We acknowledge the support of the National Institute for Health Research Clinical Research Network (NIHR CRN).

Author contributions

All authors made substantial contributions to the conception and design of the study. MVE conducted the interviews, co-facilitated the focus groups, conducted data analysis and drafted the manuscript. JJ co-facilitated the focus groups, contributed to data analysis and drafted the manuscript. HE provided supervision and guidance for data collection, contributed to data analysis and drafted the manuscript. SS, FN, WH, AK and CA critically revised the manuscript and provided intellectual input and expert advice. All authors have read and approved this manuscript.

Funding

For peer review only

702 This article presents independent research funded by the National Institute for Health
703 Research (NIHR) under the Programme Grants for Applied Research programme
704 [RP-PG-0615-20013]. The views expressed in this publication are those of the
705 author(s) and not necessarily those of the NIHR or the Department of Health and
706 Social Care.

707 **Competing interests**

708 None declared.

709 **Ethics approval**

710 This study was approved by the West Midlands – Solihull Research Ethics
711 Committee, as part of NHS Health Research Authority approvals (Reference:
712 18/WM/0050).

713 **Data availability statement**

714 Data are available upon reasonable request from the corresponding author.

715 **Abbreviations:** COREQ, consolidated criteria for reporting qualitative research; DI,
716 digital intervention; GP, general practitioner; HCP, healthcare practitioner; IMD,
717 Index of Multiple Deprivation; NHS, National Health Service; NIHR CRN, National
718 Institute of Health Research Clinical Research Network; VBI, very brief intervention.

719

720

721

722

For peer review only - http://bmjopen.bmj.com/site/about/guidelines.xhtml

References

[1] Sabaté E, World Health Organization (eds). *Adherence to long-term therapies: evidence for action*. Geneva: World Health Organization, 2003.

[2] Chowdhury R, Khan H, Heydon E, et al. Adherence to cardiovascular therapy: a meta-analysis of prevalence and clinical consequences. *Eur Heart J* 2013; 34: 2940–2948.

[3] Gupta Pankaj, Patel Prashanth, Štrauch Branislav, et al. Risk Factors for Nonadherence to Antihypertensive Treatment. *Hypertension* 2017; 69: 1113–1120.

[4] Forouzanfar MH, Liu P, Roth GA, et al. Global Burden of Hypertension and Systolic Blood Pressure of at Least 110 to 115 mm Hg, 1990-2015. *JAMA* 2017; 317: 165–182.

[5] Mills KT, Stefanescu A, He J. The global epidemiology of hypertension. *Nat Rev Nephrol* 2020; 16: 223–237.

[6] Gakidou E, Afshin A, Abajobir AA, et al. Global, regional, and national comparative risk assessment of 84 behavioural, environmental and occupational, and metabolic risks or clusters of risks, 1990–2016: a systematic analysis for the Global Burden of Disease Study 2016. *The Lancet* 2017; 390: 1345–1422.

[7] Brown MT, Bussell JK. Medication Adherence: WHO Cares? *Mayo Clin Proc* 2011; 86: 304–314.

[8] Horne R, Weinman J, Barber N, et al. Concordance, adherence and compliance in medicine taking.

[9] Clifford S, Barber N, Horne R. Understanding different beliefs held by adherers, unintentional nonadherers, and intentional nonadherers: application of the Necessity-Concerns Framework. *J Psychosom Res* 2008; 64: 41–46.

[10] Choudrie J, Pheeraphuttranghkoon S, Davari S. The Digital Divide and Older Adult Population Adoption, Use and Diffusion of Mobile Phones: a Quantitative Study. *Inf Syst Front* 2020; 22: 673–695.

[11] Baird B, Charles A, Honeyman M, et al. Understanding pressures in general practice. 100.

[12] King R, Ryan T, Senek M, et al. The impact of COVID-19 on work, training and well-being experiences of nursing associates in England: A cross-sectional survey. *Nurs Open*; n/a. DOI: 10.1002/nop2.928.

[13] Thakkar J, Kurup R, Laba T-L, et al. Mobile Telephone Text Messaging for Medication Adherence in Chronic Disease: A Meta-analysis. *JAMA Intern Med* 2016; 176: 340–349.

For peer review only

[14] Armitage LC, Kassavou A, Sutton S. Do mobile device apps designed to support medication adherence demonstrate efficacy? A systematic review of randomised controlled trials, with meta-analysis. *BMJ Open* 2020; 10: e032045.

[15] McLean G, Band R, Saunderson K, et al. Digital interventions to promote self-management in adults with hypertension systematic review and meta-analysis. *J Hypertens* 2016; 34: 600–612.

[16] Xiong S, Berkhouse H, Schooler M, et al. Effectiveness of mHealth Interventions in Improving Medication Adherence Among People with Hypertension: a Systematic Review. *Curr Hypertens Rep* 2018; 20: 86.

[17] Hardeman W, Houghton J, Lane K, et al. A systematic review of just-in-time adaptive interventions (JITAIs) to promote physical activity. *Int J Behav Nutr Phys Act* 2019; 16: 31.

[18] Horne R, Cooper V, Wileman V, et al. Supporting Adherence to Medicines for Long-Term Conditions. *Eur Psychol* 2019; 24: 82–96.

[19] Cornet VP, Holden RJ. Systematic review of smartphone-based passive sensing for health and wellbeing. *J Biomed Inform* 2018; 77: 120–132.

[20] Tong A, Sainsbury P, Craig J. Consolidated criteria for reporting qualitative research (COREQ): a 32-item checklist for interviews and focus groups. *Int J Qual Health Care* 2007; 19: 349–357.

[21] Cramer JA, Benedict Á, Muszbek N, et al. The significance of compliance and persistence in the treatment of diabetes, hypertension and dyslipidaemia: a review. *Int J Clin Pract* 2008; 62: 76–87.

[22] Kassavou A, Sutton S. Reasons for non-adherence to cardiometabolic medications, and acceptability of an interactive voice response intervention in patients with hypertension and type 2 diabetes in primary care: a qualitative study. *BMJ Open* 2017; 7: e015597.

[23] Jamison J, Naughton F, Gilbert H, et al. Delivering Smoking Cessation Support by Mobile Phone Text Message: What Information do Smokers Want? A Focus Group Study. *J Appl Biobehav Res* 2013; 18: 1–23.

[24] Morton K, Sutton S, Hardeman W, et al. A Text-Messaging and Pedometer Program to Promote Physical Activity in People at High Risk of Type 2 Diabetes: The Development of the PROPELS Follow-On Support Program. *JMIR MHealth UHealth* 2015; 3: e105.

[25] Eborall H, Morton K. Use of Focus Groups in Developing Behavioural mHealth Interventions: A Critical Review. In: Barbour RS, Morgan DL (eds) *A New Era in Focus Group Research: Challenges, Innovation and Practice*. London: Palgrave Macmillan UK, pp. 109–127.

For peer review only

[26] Britten N. Qualitative Research: Qualitative interviews in medical research. *BMJ* 1995; 311: 251–253.

[27] Charmaz K. *Constructing Grounded Theory: A Practical Guide through Qualitative Analysis*. SAGE, 2006.

[28] PAM - Programme on Adherence to Medication. *Primary Care Unit*, https://www.phpc.cam.ac.uk/pcu/research/research-groups/bsg/research-themes/medication-adherence/pam-programme-on-adherence-to-medication/ (accessed 1 October 2020).

[29] Kassavou A, A'Court CE, Chauhan J, et al. Assessing the acceptability of a text messaging service and smartphone app to support patient adherence to medications prescribed for high blood pressure: a pilot study. *Pilot Feasibility Stud* 2020; 6: 134.

[30] National Institute for Health and Care Excellence. Evidence Standards Framework for Digital Health Technologies., https://www.nice.org.uk/Media/Default/About/what-we-do/our-programmes/evidence-standards-framework/digital-evidence-standards-framework.pdf (2019, accessed 16 July 2020).

[31] Leon N, Surender R, Bobrow K, et al. Improving treatment adherence for blood pressure lowering via mobile phone SMS-messages in South Africa: a qualitative evaluation of the SMS-text Adherence SuppoRt (StAR) trial. *BMC Fam Pract* 2015; 16: 80.

[32] Barber N. Should we consider non-compliance a medical error? *Qual Saf Health Care* 2002; 11: 81–84.

[33] Horne R, Chapman SCE, Parham R, et al. Understanding Patients' Adherence-Related Beliefs about Medicines Prescribed for Long-Term Conditions: A Meta-Analytic Review of the Necessity-Concerns Framework. *PLOS ONE* 2013; 8: e80633.

[34] Nelson LA, Mulvaney SA, Johnson KB, et al. mHealth Intervention Elements and User Characteristics Determine Utility: A Mixed-Methods Analysis. *Diabetes Technol Ther* 2017; 19: 9–17.

[35] Hirschey J, Bane S, Mansour M, et al. Evaluating the Usability and Usefulness of a Mobile App for Atrial Fibrillation Using Qualitative Methods: Exploratory Pilot Study. *JMIR Hum Factors* 2018; 5: e13.

[36] Grindrod KA, Li M, Gates A. Evaluating User Perceptions of Mobile Medication Management Applications With Older Adults: A Usability Study. *JMIR MHealth UHealth*; 2. Epub ahead of print 14 March 2014. DOI: 10.2196/mhealth.3048.

[37] Nelson LA, Bethune MC, Lagotte AE, et al. The Usability of Diabetes MAP: A Web-delivered Intervention for Improving Medication Adherence. *JMIR Hum Factors* 2016; 3: e13.

For peer review only

[38] Lucivero F, Jongsma KR. A mobile revolution for healthcare? Setting the agenda for bioethics. *J Med Ethics* 2018; 44: 685–689.

[39] Lupton D. The digitally engaged patient: Self-monitoring and self-care in the digital health era. *Soc Theory Health* 2013; 11: 256–270.

[40] Szinay D, Jones A, Chadborn T, et al. Influences on the Uptake of and Engagement With Health and Well-Being Smartphone Apps: Systematic Review. *J Med Internet Res* 2020; 22: e17572.

[41] Naughton F, Hopewell S, Lathia N, et al. A Context-Sensing Mobile Phone App (Q Sense) for Smoking Cessation: A Mixed-Methods Study. *JMIR MHealth UHealth* 2016; 4: e106.

[42] Boonstra TW, Nicholas J, Wong QJ, et al. Using Mobile Phone Sensor Technology for Mental Health Research: Integrated Analysis to Identify Hidden Challenges and Potential Solutions. *J Med Internet Res* 2018; 20: e10131.

[43] Fortuna RJ, Nagel AK, Rocco TA, et al. Patient Experience With Care and Its Association With Adherence to Hypertension Medications. *Am J Hypertens* 2018; 31: 340–345.

[44] Haskard Zolnierek KB, DiMatteo MR. Physician Communication and Patient Adherence to Treatment: A Meta-Analysis. *Med Care* 2009; 47: 826–834.

[45] Harmon G, Lefante J, Krousel-Wood M. Overcoming barriers: the role of providers in improving patient adherence to antihypertensive medications. *Curr Opin Cardiol* 2006; 21: 310–315.

[46] 1 Recommendations | Behaviour change: individual approaches | Guidance | NICE, https://www.nice.org.uk/guidance/ph49/chapter/1-Recommendations (accessed 4 May 2021).

[47] Carson KV, Verbiest ME, Crone MR, et al. Training health professionals in smoking cessation. *Cochrane Database Syst Rev*. Epub ahead of print 2012. DOI: 10.1002/14651858.CD000214.pub2.

For peer review only

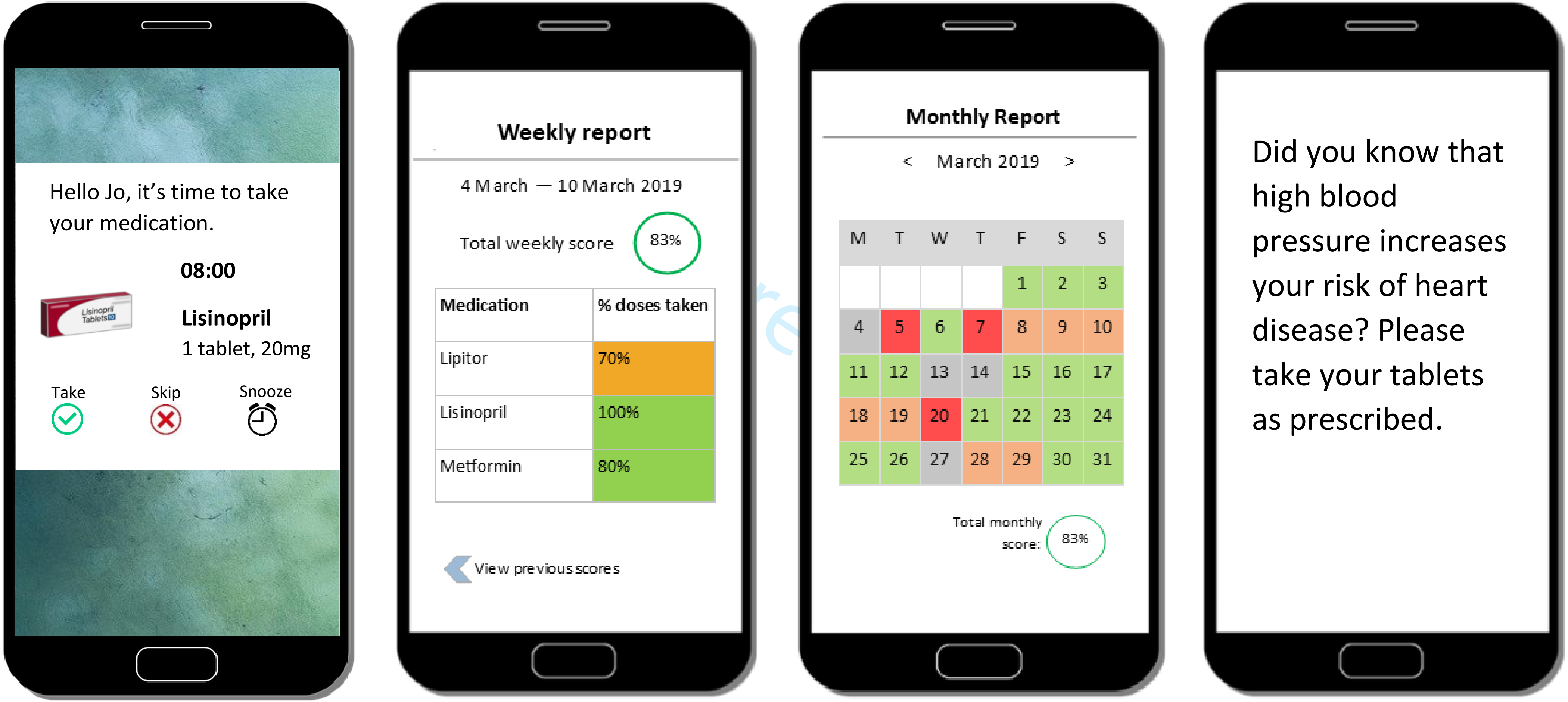
Hello Jo, it's time to take your medication.
08:00
Lisinopril
1 tablet, 20mg
Take
Skip
Snooze
Weekly report
4 March — 10 March 2019
Total weekly score
83%
Medication
% doses taken
Lipitor
70%
Lisinopril
100%
Metformin
80%
View previous scores
Monthly Report
< March 2019 >
M T W T F S S
1 2 3
4 5 6 7 8 9 10
11 12 13 14 15 16 17
18 19 20 21 22 23 24
25 26 27 28 29 30 31
Total monthly score:
83%
Did you know that high blood pressure increases your risk of heart disease? Please take your tablets as prescribed.

Supplementary file 1: Consolidated criteria for reporting qualitative studies (COREQ): 32-item checklist

**Item**	**Guide questions**	**Description**	**Reported on page no.**
**Domain 1: Research team and reflexivity**			
Personal Characteristics			
1. Interviewer/facilitator	Which author/s conducted the interview or focus group?	One author (MVE) conducted the semi-structured interviews. Two authors co-facilitated the focus groups (MVE and JJ).	8, 28
2. Credentials	What were the researcher's credentials? E.g. PhD, MD	BSc (MVE), BSc, PhD (JJ)	N/A
3. Occupation	What was their occupation at the time of the study?	Research Assistant (MVE) and Research Associate (JJ)	N/A
4. Gender	Was the researcher male or female?	Female (MVE) and male (JJ)	N/A
5. Experience and training	What experience or training did the researcher have?	MVE's training in qualitative research has included: BSc undergraduate modules; Oxford University Introduction to Qualitative Research; University of Cambridge Social Sciences Research Methods Centre training course. JJ is experienced in conducting focus groups (JJ)	N/A
Relationship with participants			
6. Relationship established	Was a relationship established prior to study commencement?	Participants had no relationship with the researcher. Patients contacted MVE via telephone or email to arrange participation in the study. HCPs* met with MVE face-to-face during a set up meeting at the GP practice, prior to interview.	7-8

For peer review only - http://bmjopen.bmj.com/site/about/guidelines.xhtml

7. Participant knowledge of the interviewer	What did the participants know about the researcher? e.g. personal goals, reasons for doing the research	Participants knew the reasons for conducting the research, as detailed in the patient information sheet and discussed prior to commencing the interview/focus group.	N/A
8. Interviewer characteristics	What characteristics were reported about the interviewer/facilitator? e.g. Bias, assumptions, reasons and interests in the research topic	Participants were aware that the research aimed to inform the development of an intervention to support medication taking. Researchers were unable to avoid bias regarding prior knowledge of the research literature around medication adherence.	N/A
**Domain 2: study design**			
Theoretical framework			
9. Methodological orientation and Theory	What methodological orientation was stated to underpin the study? e.g. grounded theory, discourse analysis, ethnography, phenomenology, content analysis	Analysis was informed methodologically by the constant comparative approach[1] and theoretically by Perceptions and Practicalities Approach (PAPA)[2].	9
Participant selection			
10. Sampling	How were participants selected? e.g. purposive, convenience, consecutive, snowball	Patients were eligible if they were prescribed medication to treat hypertension and used SMS messages or smartphone apps. A random sample of patients were selected by GP practice administrators to receive the study invitation pack. HCPs were eligible if they were involved with the management or care of patients with hypertension, including medication reviews or hypertension checks. HCPs were a convenience sample from the four GP practices taking part in the study.	7-8

For peer review only - http://bmjopen.bmj.com/site/about/guidelines.xhtml

11. Method of approach	How were participants approached? e.g. face-to-face, telephone, mail, email	Patients received an invitation letter from their GP practice, or saw study advertisement posters in the waiting room. HCPs were invited to take part during a face-to-face meeting with the researcher (MVE) at their GP practice.	7-8
12. Sample size	How many participants were in the study?	20 patients and 11 HCPs	11
13. Non-participation	How many people refused to participate or dropped out? Reasons?	All participants who responded to the study invitation and set a date/time for participation, took part in either an interview or focus group.	N/A
Setting			
14. Setting of data collection	Where was the data collected? e.g. home, clinic, workplace	Patient interviews: participant's homes (n=4), workplace (N =1) and a local library (n=1).<br>HCP interviews: their place of work (n=11 HCP)<br>Focus groups: local community centres (n=14, across 4 groups)	9
15. Presence of non-participants	Was anyone else present besides the participants and researchers?	Only the participants and researchers were present during the study.	N/A
16. Description of sample	What are the important characteristics of the sample? e.g. demographic data, date	Participant characteristics are reported in Tables 1 and 2.	Tables 1 and 2
Data collection			
17. Interview guide	Were questions, prompts, guides provided by the authors? Was it pilot tested?	The interview and focus group schedules were guided by the principles of PAPA and informed by the aims of the intervention development team. Interview and focus group schedules were reviewed by the research team and PPI representatives.	9-10, supplementary files 2a, 2b, 2c
18. Repeat interviews	Were repeat interviews carried out? If yes, how	No repeat interviews were carried out.	N/A

For peer review only - http://bmjopen.bmj.com/site/about/guidelines.xhtml

	many?		
19. Audio/visual recording	Did the research use audio or visual recording to collect the data?	Interviews and focus groups were audio-recorded.	10
20. Field notes	Were field notes made during and/or after the interview or focus group?	Field notes were made during and/or after each interview and focus group.	N/A
21. Duration	What was the duration of the interviews or focus group?	Mean interview duration was 47 minutes (range 21 to 75). Mean focus group duration was 88 minutes (range 83 to 91).	10
22. Data saturation	Was data saturation discussed?	Recruitment of participants continued until no new themes were emerging in relation to the specific topics covered.	12
23. Transcripts returned	Were transcripts returned to participants for comment and/or correction?	Transcripts were not returned to participants for comment and/or correction	N/A
**Domain 3: analysis and findings**			
Data analysis			
24. Number of data coders	How many data coders coded the data?	Data was coded by MVE and JJ, supervised by HE. Findings were discussed with the research team.	10-11, 28
25. Description of the coding tree	Did authors provide a description of the coding tree?	A sample of transcripts were read thoroughly to identify broad codes. Transcripts were compared in turn with codes already identified to refine the coding framework. Codes were then grouped into themes.	N/A
26. Derivation of themes	Were themes identified in advance or derived from the data?	We generated general descriptive categories from a subsample of transcripts. These were either derived directly from the data, or were pre-defined from the literature review, interview schedule questions and/or aims of the intervention development. This meant that some	10

For peer review only

		categories and concepts had increased sensitivity.	
27. Software	What software, if applicable, was used to manage the data?	Data was managed using NVivo 12.	10
28. Participant checking	Did participants provide feedback on the findings?	Participants did not provide feedback on the findings.	N/A
Reporting			
29. Quotations presented	Were participant quotations presented to illustrate the themes/findings? Was each quotation identified? e.g. participant number	Participant quotations are presented in the main text to illustrate the findings. Each quotation is identified using a participant ID. A table of extra quotations from participants is presented in the supplementary materials.	See Results section, and Supplementary file 2.
30. Data and findings consistent	Was there consistency between the data presented and the findings?	There was consistency between the data presented and the findings.	See Results section.
31. Clarity of major themes	Were major themes clearly presented in the findings?	Major themes are identified at the beginning of the Results section, and referred to throughout.	See Results section.
32. Clarity of minor themes	Is there a description of diverse cases or discussion of minor themes?	Minor themes are explored alongside the major themes.	See Results section.

* **Note:** HCP = healthcare practitioner

Developed from: Tong A, Sainsbury P, Craig J. Consolidated criteria for reporting qualitative research (COREQ): a 32-item checklist for interviews and focus groups. *International Journal for Quality in Health Care*. 2007. Volume 19, Number 6: pp. 349 – 357

References

1. Charmaz K. *Constructing Grounded Theory: A Practical Guide through Qualitative Analysis*. SAGE; 2006.
2. Horne R, Cooper V, Wileman V, Chan A. Supporting Adherence to Medicines for Long-Term Conditions. *Eur Psychol*. 2019;24(1):82-96. doi:10.1027/1016-9040/a000353

For peer review only - http://bmjopen.bmj.com/site/about/guidelines.xhtml

Supplementary file 2a

Example digital intervention messages

Medication reminder	Hello Jo, it's time to take your Lisinopril 20mg this morning. Thank you.
Feedback	Glad to see you're taking your blood pressure medication. Keep up the good work!
Information	Did you know that high blood pressure increases your risk of heart disease? Please take your tablets as prescribed.
Support	Have you taken all your prescribed tablets today? If you need support, reply 'HELP' to this number.

Note: Example messages were created as part of a wider NIHR-funded programme grant. For more information about the intervention content and functions, see Kassavou, A'Court, Chauhan et al (2020) https://pilotfeasibilitystudies.biomedcentral.com/articles/10.1186/s40814-020-00666-2

For peer review only

Topic guide: patient interviews

In the each interview, the interviewer will remind the participant of the project aims and check that they fully understand the participant information sheet, and will then take them through the informed consent process. Audio-recording of the interview will then begin.

Part 1: Questions to assess participant's experience with taking their medication

First I want to ask you about the medications that you're taking at the moment.

- How many medications have you been prescribed and what medications do you take?
  - How many doses do you need to take and how often?
- What would you say are the benefits of taking your medication?
- What about the downside of /problems with taking your medications?
  - How does this affect whether you take your medication or not?
- You said you're meant to take [XX] medications a day. How easy it is to remember to take them?
  E.g. prompts
  - Do you have a particular routine? If so, what is it?
- What about altering the dose, is this something that you ever do? [can you tell me more about that? Which medication? How do you alter it? Can you tell me why you alter it in that way? Can you think of any other reason why you don't take your tablets regularly? E.g. side effects]
- Is there anything that helps you to take you medication? Anything that makes it easier?

Part 2: Views on an intervention to support medication adherence

- Interviewer will outline the basic structure of the proposed intervention, using visual aid/schematic to help explain.

For peer review only - http://bmjopen.bmj.com/site/about/guidelines.xhtml

  - A very brief face-to-face session with a practice nurse or healthcare assistant followed by ongoing support via mobile/internet.

- How would you feel about something like this that could support you to take your medication as prescribed?
  - Via text message?
  - Via an app?
- Which of these would be best for you? [e.g. prefer app or text?]
- What sort of messages would you find most helpful for helping you to take your tablets?

Example materials

- *Show participants examples of digital interventions*
  - Prompt questions
    - Which types of messages would you find most useful for helping you to take your medications? Why/why not?
    - What types of messages would be unhelpful? And why?
  - How could these messages be improved? Or reworded?
  - What messages would be useful for someone who doesn't want to take their pills? (i.e. INA)
  - How many messages per day or per week?

  - What about information / any other features that could be included? *E.g. probes:*
    - Feedback on your adherence level (e.g. graph, table % score, which prefer?)
    - Involve a carer/significant other?
    - Make use of location information

Part 3 – User engagement

- What would encourage you to start using something like this? *(Prompt initiation ideas such as demonstrating SMS/app during consultation, downloading app during consultation)*
  - Is there anything that would prevent or make it difficult for you using it?
  - Would a tutorial help you to understand how to use the SMS/app? How would you prefer to access this tutorial?
  - What would encourage you to keep using the app/text service long term?

For peer review only - http://bmjopen.bmj.com/site/about/guidelines.xhtml

Supplementary file 2b

Topic guide: healthcare practitioner interviews

In the each interview, the interviewer will remind the participant of the project aims and check that they fully understand the participant information sheet, and will then take them through the informed consent process. Audio-recording of the interview will then begin.

Part 1: Questions to ascertain participant's experience with addressing non-adherence issues with their patients

- Can you describe your role in the GP Practice?
- How involved are you with prescribing and monitoring patients' medication?
- How do you usually ascertain whether a patient is taking their medication as prescribed?
- What do you tend to do if/when you think a patient is not taking their medication as prescribed?

  E.g. probes:

  - Medication-taking can be perceived as a sensitive topic, how do you tend to address this when starting your conversation?
  - Do you explore patients' reasons for not taking it; how easy/difficult is this?
  - What challenges do you come across?
  - What do you think can help support patients in their medication taking?

Part 2: Views on an intervention to support medication adherence

Interviewer will outline the basic structure of the proposed intervention, using visual aid/schematic to help explain.

  - *A very brief face-to-face session with a practice nurse or healthcare assistant or pharmacist followed by (ii) ongoing support via mobile/internet.*
  - *Include a draft outline of the 5 minute face-to-face consultation between HCP and patient.*

- How comfortable/confident would you feel in delivering this?

  E.g. probes:

  - *Go through draft outline of VBI line by line.*
    - E.g. tailoring questionnaire
    - What information would you include in the pamphlet?
    - Is there anything you would add/remove?
  - What about doing this all in 5 minutes?
  - Would you find this task demanding? If yes, how could we make this easier for you? (E.g. tools/prompts/scripts- and how best to present these- bullet points?)
  - What kind of training would you find helpful? (E.g. multiple sessions/feedback/group work)

For peer review only - http://bmjopen.bmj.com/site/about/guidelines.xhtml

Interviewer will outline the idea of a digital intervention [possibly using visual aid/schematic to help explain how/when this would happen]

- What are your thoughts on how patients would get on with an intervention like this?
    - How useful do you think it would be in supporting patients to take their medications between their primary care consultations?
        - *E.g. probes:* helpful/unhelpful messages
    - What do you think patients need from an intervention like this?
    - If you were designing text messages/phone app, what content or materials would you include?

Part 3: Incorporating into a consultation

- What would encourage you to recommend a text message service/app to your patients?
    - What thoughts do you have on encouraging patients to use it / and keeping them engaged?
    - Would you find it helpful to trial the digital intervention yourself before recommending to patients?
- How might an intervention be incorporated into a consultation at the GP practice?
    - Which routine consultation would be appropriate?
    - What thoughts do you have on how to sign the patients up?
- Are there any specific patients who might benefit from an intervention more, or less, than others?

For peer review only

For peer review only - http://bmjopen.bmj.com/site/about/guidelines.xhtml

Supplementary file 2c

Topic guide: patient focus groups

Introduction

- Consent etc. (audio recording - ensure participants are comfortable with this before starting, and 'ground rules' of FGs.)
- Begin audio recording.
- Describe the PAM intervention. Will keep brief and in lay terms.
- We want to find out what you think about some of the app features

- Before we start, can I ask how many medications you've each been prescribed?
  - How many per day?
  - Morning/evening doses?
- How much you use your smartphones?
- Do you use any Apps that have reminder message pop up (e.g. calendar, med reminder app etc)?
  - Prompt: do these have alerts? How often do you use these apps?

1. Reminder notification messages

I'd like you to look at these examples [medication reminder messages]

- Go through each feature for each scenario (the text, layout, image, size, etc.) what do you like/dislike? What would you change?

Message content

- Is there anything missing from this message?
- What would you remove from this message?
- Which is your favourite and why?

Snooze options

Do you know what a snooze option is (e.g. like your alarm clock) [if not explain it's a feature we can add which allows you to delay the notification for extra time, similar to an alarm clock.]

- Let's say you're able to snooze the medication reminder message. How useful would you find this function? In which situations might you use it?
  - [prompts could include how long snooze should be etc.]

Images of medication

- Would you find it useful to have any of these images of your medications in the App?
- Would you like to be able to take your own photo and upload it?

Types of medication

- If you have more than one medication….
  - How would you feel about receiving reminder messages ONLY for medication related to HBP?

For peer review only - http://bmjopen.bmj.com/site/about/guidelines.xhtml

    - Would you want the option to choose which medications you receive reminders for?
- Do your medications ever change? What about if your medications change- how would you like to handle that?
    - Update the app yourself?

Message timings

- What time would you like to receive these messages?
    - Would you want to be able to change the timings yourself?
    - How comfortable would you feel doing this?
    - How about weekday vs weekends?

Message frequency

- When talking about reminder messages, how many of these would you like to receive per day?
- What about for non-reminder messages? (E.g. messages with advice and support to motivate people to take their medication)

Engagement and 'honest' reporting!

- We know that some people can get fed up with these types of apps, and messages on your phone every day.
- What would encourage you to use the app every day, say for a month? Or 3 months?
    - What would discourage you?
- What about stopping/reducing reminders?
    - When/why might you want the messages reducing and/or stopping?

- Some people might get a notification telling them a dose is due, think, "I want to skip this dose".
- How honest do you think people would really be when responding in the app?
    - How can we encourage people to use it 'honestly'
        - Are there any occasions where people might 'lie'?
        - Prompts (e.g. assure people that their GP won't see what they say)

2. Feedback of adherence

Apps can give you feedback on your results using different visual formats (e.g. table, graph, list, monthly calendar – show examples)

Prompts:

- Which would you prefer? And why?
- Total % score – helpful?
- Would you use this feature?
- What information do you want to see in the 'feedback'?
- What other methods could the app use to feedback reports?
- Would you find weekly or monthly feedback more helpful? Why?

For peer review only - http://bmjopen.bmj.com/site/about/guidelines.xhtml
For peer review only

3. Sensing data

- Now we would like to hear your thoughts on 'sensing technology'.
- Do you know much about the sensors in our smartphones?

- *[Facilitator explains sensors]*
- *All smartphones have sensors built into the device, which can gather data on how the phone is being used, such as movement and light (e.g. detect if it's face up or down).*
- *A simple example is when you make a call, and hold the phone to your ear, do you notice that the touchscreen automatically switches off, to stop you pressing buttons with your cheek. This happens because a sensor within the phone can detect when an object is close to its screen.*
- *Another example is how some phones have an inbuilt pedometer which can count the number of steps walked each day, by detecting the 'walking' movement of your phone.*

- *[Explain sensors re tailoring the App]*
- *Sensing technology is a novel way to personalise apps to your everyday behaviour.*
- *E.g. with this app, we can use sensors to personalise the messages sent to you, based on information collected from sensors in your phone.*
- *Provide examples of data that could be collected by the app*
- *Location data.*
    - *Detected either by WiFi (e.g. your home WiFi or a café WiFi) or GPS (detecting the location of the phone, like when we use Google Maps and satnavs).*
    - *The app could use this to send you messages at appropriate times, e.g. only when you're at home.*
- *Movement of phone or pedometer*
    - *e.g. App will detect movement of the phone (running/walking) and will delay a reminder until you've finished*

- How would you feel about an app collecting this data?
    - Would it put you off using the app?
- Is there anything that we could do to encourage you to like/change your mind about this feature?
- Is there anything you don't like about this feature?
- What questions would you have about this? (this will help us to inform the leaflet for ppts)
- What information should we provide for users? How detailed? How? (e.g. website/leaflet)

Close

For peer review only - http://bmjopen.bmj.com/site/about/guidelines.xhtml

Supplementary file 3

Draft outline of a 5-minute face-to-face consultation (VBI)

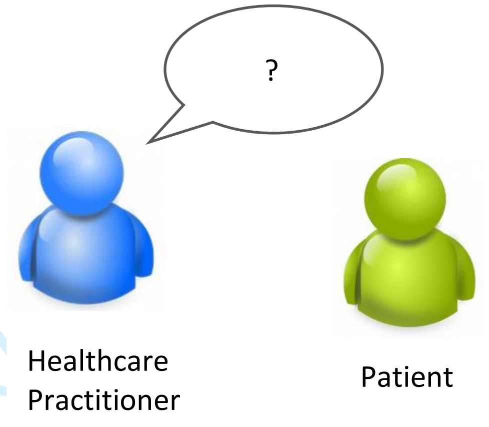

- Start the consultation with de-stigmatising medication non-adherence – forgetting to take tablets and/or having concerns is common.
- Support is available. Introduce the digital support (SMS texts or mobile phone app) as a (fun?) way to help patients take them – perhaps share personal experience with using it, demonstrate a key feature of the app/ text-messaging support.
- Complete short online questionnaire on behalf of patient:
    - Input basic information (patient name, age, mobile phone number)
    - Choose from drop down list of medications that patient is prescribed (tablet name, dose, frequency)
    - Ask patients to give their main reasons for not taking their medication (e.g. choose top 3 reasons from a list, or rank from most important to least important).
- Generate a code for patients to download the app/start the support. E.g. QR code, link to website for patients to download app.
- Hand out pamphlet with further information.
- Endorse the patients' use of digital support.
- Record in the system for follow-up purposes.

For peer review only - http://bmjopen.bmj.com/site/about/guidelines.xhtml

Supplementary file 4: Major themes, sub themes and illustrative quotes

Theme	Sub theme	Example participant quote
**Reasons for non-adherence**	*Non intentional non-adherence (NINA)*<br>Forget to take medication and/or reorder prescription	If I don't put the blister pack back and take the tablets, sometimes I can't remember if I've taken it or not [P03, male, 60s]<br><br>I just totally forgot to take my medication this morning. I just had a cup of tea and a piece of toast. I have to admit I was in a hurry today, I'm sorry, I didn't take the BP [medication]. [P06, female, 60s]<br><br>Usually it's that they haven't had a chance to put their prescription in, so then they tend to run low. [HCP 07, Practice Nurse, female]
	Intentional non-adherence (INA)<br>Unpleasant side effects; general reluctance to take medication	Sometimes I'm tempted to, and if I'm doing some sporting activity, I will be less inclined to take them. They seem to reduce my performance […] perhaps take it after I've indulged in the sport. [P04, male, 40s]<br><br>Often patients will say that they felt fine before they took their blood pressure medicine and now they feel rotten. [HCP 06, Practice Nurse, female]<br><br>Especially with metformin, because it can often have some unpleasant side-effects, and very often people will stop taking it because of this, but they don't actually report that fact to us. [HCP 10, Practice Nurse, female]<br><br>You know, I'm sure there's a lot of natural stuff out there that could help me you know. I don't know what effect it's having inside. That's the trouble, you know…what, in fact, it's doing. [P02, male, 60s]<br><br>I just hate myself for having to take them… I just feel that there must be a natural way… I just feel that, you know, lifestyle choices would be a better way than taking tablets, and that's why I hate myself for having to do it. [P03, male, 60s]

For peer review only

**Recommendations for intervention content**	Simple reminders to take medication and to reorder prescription (NINA)	It is good about the reminder because I sometimes say to my husband, er, would you remind me and he doesn't because he's worse than I am. [P05, female, 70s]<br><br>I think that would be quite good if it could tell you when you last got your prescription and when your next prescription's due and send a reminder maybe a few days before to say. [FG3, male]<br><br>Do you know what would be handy, is the reminder to tell you when you're gonna run out. That would be good for me. [FG1, male]
	Messages containing information about medication: consequences of non-adherence (targeting INA) acceptable to HCPs but not to patients; preference for advice on how to follow up a missed dose	I guess they need a more educational approach...that's the kind of need to explore why they are choosing not to take it. Do they understand the consequences of not taking it? So the nature of the messaging might be quite sort of shock, 'if you do not take your aspirin you are at risk of having a heart attack', or something like that. (HCP 02, GP, male)<br><br>It's like preaching, and I don't need my phone to be beeping at me in order to preach a message. [FG4, female]<br><br>I don't know whether I could...whether it's okay to do a catch-up, like if I missed my Ramipril in the morning, can I take that and the Indapamide at night? Or would that be too high a dose in one go? So, you know, maybe that needs to be addressed in the texts [P03, male, 60s]<br><br>Other days I've forgotten completely and then I get home and I think, "Shall I take 'em now or shall I wait 'til tomorrow now?" I've done that once or twice. (M)<br>That is a problem, whether you can take it if you've forgotten. (F)<br>I want to know precisely, "Well, will that affect it big or a lot or not a lot or...? And is taking them all together more important than the hour?" […] It's the occasional expert knowledge that motivates people to stick with an app isn't it? […] It's the fact that if you could press a button and say, "What should I do now 'cos I've forgotten 'em?" might be just a great help. (M) [FG3]

For peer review only - http://bmjopen.bmj.com/site/about/guidelines.xhtml

	Feedback on adherence levels – simple statistics preferred	That could be a graph, couldn't it? Or a percentage. You know, 'well done', you know, 'you're ninety-five per cent this month'. [P03, male, 60s]<br><br>Even a weekly graph might be quite nice. Because, then you can see that, actually, when I started a week ago, I was taking fifty per cent…and now I'm going up, and you know, just a visual kind of thing for them. And, actually, now I'm on a hundred per cent, and next week I'm hundred per cent. [HCP 01, Practice Pharmacist, female]<br><br>For me, I think the answer probably is yes actually, because actually that really would be a... you know, a wake-up call, thinking, "God, [participant name], do you realise that's two days this week?" I mean, okay, I should be able to tell what I had but, you know –. [FG4, male]
	Signpost patients to contact their HCP	If you are having side-effects, please contact somebody, rather than just ignore it…. It could be it's the pharmacist, or the Practice Pharmacist rather than the community one. It's just about improving interactions with us, the pharmacist, reporting side-effects. [HCP 06, Practice Nurse, female]
**Tailoring the digital intervention**	Highly tailored intervention facilitates engagement with SMS messages and smartphone app	It's about tailoring the process to the patients' needs….What suits one is not going to suit the next twenty people. [HCP 06, Practice Nurse, female]<br><br>It would be nice to be a bit personalised, it makes you feel a bit special [FG1, male]<br><br>You can default [medication reminders] to all the days of the week being the same but it might be nice to be able to specify different times of the day if you really wanted to. [FG3, male]<br><br>I mean, could users perhaps make their own decision which, which [reminder message] version they want to have appear? [FG4, female]<br><br>How much margin is there to vary from person to person? So if I said I wanted the full works I could have them but perhaps somebody that didn't? [FG1, male]

For peer review only - http://bmjopen.bmj.com/site/about/guidelines.xhtml

	Barrier to engagement – if intervention is overcomplicated or difficult to use	A hundred ideas that you get but if it's not simple then, the app in the end—From personal experience it won't work, it needs to be simple for people to buy into it. [FG2, male]<br><br>But I think it has to be really simple 'cos, you know, the more you're delving into this, it's... it can get complicated, can't it? So people are just gonna want something easy. [FG3, female]
**Acceptability of sensing technology**	Facilitator – Potential for sensing technology to enhance user experience; flexible opt-in/out options	Another time that I might forget to take medication is if I've gone somewhere just overnight ... I might just forget to put it in and I think, oh, well, nothing I can do, I'll have to wait 24 hours or whatever, but if you put in your diary on your phone that you were going to then [the app] might say, "don't forget to pack your pills". [FG2, male]<br><br>I would be happy with the sensing and respond accordingly because I think we could fine tune some of it ourselves couldn't we? If it was personalised. [FG2, female]<br><br>But could people have an off button for that if they wanted, for that bit of the menu? [FG2, female]
	Barrier to acceptability – concerns about confidentiality of data; requests for information about data use	It's all about how that information is dissembled, isn't it, and so long as that information is within reason locked away somewhere [FG4, male]<br><br>I would definitely want to know you weren't passing my data onto anyone else, either for medical or marketing purposes [FG2, female]<br><br>I'd feel much happier using one that wasn't purely commercial as well. [FG2, female]

For peer review only

**Attitudes towards a VBI (HCP only)**	Barriers and facilitators to implementation: must be straightforward to incorporate and deliver within primary care; five minute time limit potentially unfeasible; sufficient training in delivery of VBI	It would have to be something that's very accessible on your desktop or as part of integrated into the clinical system. [HCP 03, Practice Nurse, female]<br><br>I think this is quite a straightforward process, as I say, because a lot of this we should be doing already and then it's the add on bit at the end. [HCP 06, Practice Nurse, female]<br><br>I don't think it's going to end at five minutes. I think it's probably going to go a bit more than that in some cases [HCP 05, Healthcare Assistant, female]<br><br>I'm quite chatty so they will probably just chat with me, 'cause I make them chat, but it could be easily done in five minutes if you were concise and just got on with it. HCP 08, Practice Nurse, female]<br><br>It would be really good that we've had the training on how to use the app, how to use the questionnaire… It might be useful even just to show us how you might do a consultation. You know, sometimes visually seeing something can be… Even just a video. [HCP 01, Practice Pharmacist, female]<br><br>Quite often what works well is just an almost practical session of: "This is what we're proposing. This is how you do the face to face consultation. This is what the apps look like" and then, you know, it then develops discussion. [HCP 06, Practice Nurse, female]<br><br>The more you do and the easier would become and you get more slick at it, wouldn't you… What we would need is a little bit more information on the type of app and text that you are going to try and set up. And maybe have a dummy run and see how comfortable we felt with it and if there was anything we could do to make it slightly more slicker and more professional… A video clip or something of somebody doing it so we would know how to approach it and address it and stuff like that, will probably be the best thing. [HCP 09, Practice Nurse, female]

For peer review only - http://bmjopen.bmj.com/site/about/guidelines.xhtml

	Recommendations for VBI: importance of HCP/patient relationship; understand the root cause of non-adherence and address patient concerns	If we have a very judgmental approach to care then we actually lose the patient's confidence and their respect and actually they won't come back at all. So that is just so important.[HCP 06, Practice Nurse, female]<br><br>You can get through if you put it in the right wording. I do find, I've been doing it for quite a few years now and I find I have got a good, you know, I'm not blowing my own trumpet, but I have got quite a good rapport with my patients [HCP 04, Healthcare Assistant, female]<br><br>I just try to keep it open and honest, and sort of gain their trust so they actually tell me why [they aren't taking their medications]. Most patients, if they know you or if they've dealt with you before, are quite happy to share… If they, um, don't think that they're going to get into trouble [laughs] for not taking it. [HCP 11, Practice Pharmacist, female]<br><br>It's about sort of exploring why, what their understanding is of the medications that they are taking and trying to get an understanding of whether they recognise the importance of compliance. So it's kind of just exploring in a deeper way as to why they've not been taking. [HCP 02, GP, male]<br><br>I tend to just try and get to the bottom of [non-adherence]… It's unpicking what the problems are and what their perception is and sometimes it's quite misguided and it's different from one person to another. [HCP 03, Practice Nurse, female]<br><br>The reasons why patients don't take their medication we should really be exploring in any case… our role in the face to face is actually we can explore some of these things a bit easier. [HCP 06, Practice Nurse, female]

Note: HCP = healthcare practitioner

For peer review only - http://bmjopen.bmj.com/site/about/guidelines.xhtml

BMJ Open

Patient and practitioner views on a combined face-to-face and digital intervention to support medication adherence in hypertension: a qualitative study within primary care

Journal:	*BMJ Open*
Manuscript ID	bmjopen-2021-053183.R2
Article Type:	Original research
Date Submitted by the Author:	06-Dec-2021
Complete List of Authors:	Van Emmenis, Miranda; University of Cambridge, Public Health and Primary Care<br>JAMISON, JAMES; University of Cambridge, Public Health and Primary Care<br>Kassavou, Aikaterini; University of Cambridge, Public Health and Primary Care<br>Hardeman, Wendy; University of East Anglia, School of Health Sciences<br>Naughton, Felix; University of East Anglia Faculty of Medicine and Health Sciences, School of Health Sciences<br>A'Court, Charlotte; University of Cambridge, Public Health and Primary Care<br>Sutton, Stephen; University of Cambridge, Public Health and Primary Care<br>Eborall, Helen; The University of Edinburgh, Usher Institute
**Primary Subject Heading**:	Public health
Secondary Subject Heading:	Qualitative research
Keywords:	QUALITATIVE RESEARCH, Hypertension < CARDIOLOGY, PRIMARY CARE, PUBLIC HEALTH

SCHOLARONE™
Manuscripts

I, the Submitting Author has the right to grant and does grant on behalf of all authors of the Work (as defined in the below author licence), an exclusive licence and/or a non-exclusive licence for contributions from authors who are: i) UK Crown employees; ii) where BMJ has agreed a CC-BY licence shall apply, and/or iii) in accordance with the terms applicable for US Federal Government officers or employees acting as part of their official duties; on a worldwide, perpetual, irrevocable, royalty-free basis to BMJ Publishing Group Ltd ("BMJ") its licensees and where the relevant Journal is co-owned by BMJ to the co-owners of the Journal, to publish the Work in this journal and any other BMJ products and to exploit all rights, as set out in our licence.

The Submitting Author accepts and understands that any supply made under these terms is made by BMJ to the Submitting Author unless you are acting as an employee on behalf of your employer or a postgraduate student of an affiliated institution which is paying any applicable article publishing charge ("APC") for Open Access articles. Where the Submitting Author wishes to make the Work available on an Open Access basis (and intends to pay the relevant APC), the terms of reuse of such Open Access shall be governed by a Creative Commons licence – details of these licences and which Creative Commons licence will apply to this Work are set out in our licence referred to above.

Other than as permitted in any relevant BMJ Author's Self Archiving Policies, I confirm this Work has not been accepted for publication elsewhere, is not being considered for publication elsewhere and does not duplicate material already published. I confirm all authors consent to publication of this Work and authorise the granting of this licence.

For peer review only

ORIGINAL RESEARCH

Patient and practitioner views on a combined face-to-face and digital intervention to support medication adherence in hypertension: a qualitative study within primary care

Miranda Van Emmenis[1], James Jamison[1], Aikaterini Kassavou[1], Wendy Hardeman[2], Felix Naughton[2], Charlotte A' Court[1], Stephen Sutton[1], Helen Eborall[3]

[1] Behavioural Science Group, University of Cambridge, United Kingdom

[2] School of Health Sciences, University of East Anglia, United Kingdom

[3] Usher Institute, University of Edinburgh, United Kingdom

Correspondence:

Miranda Van Emmenis

Behavioural Science Group, Primary Care Unit, East Forvie Building, Forvie Site, Robinson Way, Cambridge, CB2 0SR

Tel 01223 330524

Email mv404@medschl.cam.ac.uk

Word count: 5992

For peer review only - http://bmjopen.bmj.com/site/about/guidelines.xhtml

Abstract

Objectives: To explore patients' and healthcare practitioners' views about non-adherence to hypertension medication and potential content of a combined very brief face-to-face discussion (VBI) and digital intervention (DI).

Methods: A qualitative study (N=31): interviews with patients with hypertension (n=6) and healthcare practitioners (HCPs; n=11) and four focus groups with patients with hypertension (n=14). Participants were recruited through general practices in Eastern England and London. Topic guides explored reasons for medication non-adherence and attitudes towards a potential intervention to support adherence. Stimuli to facilitate discussion included example SMS messages and smartphone app features, including mobile sensing. Analysis was informed methodologically by the constant comparative approach and theoretically by Perceptions and Practicalities Approach (PAPA).

Results: Participants' overarching explanations for non-adherence were non-intentional (forgetting) and intentional (concerns about side-effects, reluctance to medicate). These underpinned their views on intervention components: messages that targeted forgetting medication or obtaining prescriptions were considered more useful than messages providing information on consequences of non-adherence. Tailoring the DI to the individuals' needs, regarding timing and number of messages, was considered important for user engagement. Patients wanted control over the DI and information about data use associated with any location sensing. While the DI was considered limited in its potential to address intentional non-adherence, HCPs saw the potential for a VBI in addressing this gap, if conducted in a non-judgemental manner. Incorporating a VBI into routine primary care was considered feasible,

For peer review only

provided it complemented existing GP practice software and HCPs received sufficient training.

Conclusions:

A combined VBI-DI can potentially address intentional and non-intentional reasons for non-adherence to hypertension medication. For optimal engagement, recommendations from this work include a VBI conducted in a non-judgmental manner and focusing on non-intentional factors, followed by a DI that is easy-to-use, highly tailored, and with provision of data privacy details about any sensing technology used.

Keywords: medication adherence, qualitative research, high blood pressure, digital intervention, healthcare professional, very brief face-to-face intervention

For peer review only - http://bmjopen.bmj.com/site/about/guidelines.xhtml

Strengths and limitations of the study

- To our knowledge, this is among the first qualitative studies to gather patient views on the use of sensing technology such as Wi-Fi or GPS within a smartphone app to support medication adherence.
- The study sought the views of a range of healthcare practitioners on incorporating a very brief intervention for medication adherence into a primary care consultation, a topic not previously explored in-depth.
- The use of stimulus materials provided detailed and focused responses for specific intervention components such as feedback on adherence and content of messages.
- While the sample size was small, the depth and focus of insights gained are sufficiently useful in informing the development and refinement of intervention components.

Introduction

Medication adherence, defined as the level to which an individual takes medication as intended by their healthcare prescriber, is a worldwide public health concern.[1] Non-adherence to blood pressure lowering medication is estimated at 41%, which is relatively high compared with many other medications.[2, 3] This is associated with increased risk of cardiovascular disease related morbidity and mortality.[4, 5] Given that high blood pressure is responsible for nearly 20% of deaths worldwide, non-adherence to antihypertensive treatment is a global health concern.[6]

For peer review only

89 Previous research into medication non-adherence has documented its complexity and multifaceted nature.[7, 8] Two broad categories within this are (i) non-intentional non-adherence, a passive process due to factors not directly within an individual's control, such as memory or access difficulties,[8, 9] and (ii) intentional non-adherence, a more deliberate action whereby an individual makes a conscious decision not to take their medication due to their perceptions about or experiences with their medication or condition.[9]

96 The multifaceted nature of non-adherence presents a challenge to those developing interventions to support adherence; for example, determining which factors to target, while balancing feasibility of delivery with likely effectiveness. Digital interventions (DIs) such as SMS text messaging or smartphone applications (apps) offer interactive, low cost and scalable methods of providing support to individuals for whom medication adherence is a challenge. DIs are particularly suitable given the increasing use of these by people across the age groups for day-to-day tasks, such as apps for alarm clocks, calendars and shopping lists.[10] In addition, DIs can potentially lower costs compared to traditional face-to-face approaches through reducing or eliminating consultation time required with healthcare practitioners, which may be particularly valuable at times when there is a high demand for consultations e.g. during the current Covid pandemic. [11, 12]

108 Evidence for the effectiveness of DIs in improving medication adherence is promising (e.g. see Thakkar et al [13]). In a recent systematic review of app-based interventions, patients using a smartphone app to support medication adherence for various health conditions were twice as likely to report taking their medications than those receiving usual care.[14] Specific to hypertension, DIs such as SMS messages, smartphone apps, email and Bluetooth blood pressure monitors have

For peer review only - http://bmjopen.bmj.com/site/about/guidelines.xhtml

been shown to improve medication adherence and lower both diastolic and systolic blood pressure.[15, 16]

Incorporating sensing technology into smartphone apps potentially expands the scope of DIs further. Passive smartphone sensors can collect user location data via GPS or Wi-Fi to enable the delivery of real-time support [17], which is of particular relevance given that non-intentional non-adherence is strongly influenced by a person's physical environment.[18] Smartphone sensing technology has shown success in DIs across the domain of health and well-being (e.g. see Cornet and Holden for a review [19]) but user acceptability of such technology in a smartphone app to support medication adherence is largely unknown.

While user acceptability is key to use of a DI, potential users first need to install and engage with the DI for it to provide benefit. Primary care professionals, such as practice nurses or community pharmacists are ideally placed for encouraging uptake of DIs for medication adherence, for example during a medication review or at the point of prescription collection. A DI used as an adjunct to a face-to-face consultation might therefore be a promising approach to support medication adherence. There is some evidence that DIs combined with tailored tele- or web-based feedback from healthcare practitioners, improves adherence to long-term medication [20] and antihypertensive medication.[21] However, evidence is limited on how healthcare professionals can best promote the uptake of DIs for medication adherence. The acceptability of combining a DI with a very brief face-to-face intervention delivered by a healthcare professional to support medication adherence has also not been widely explored.

For peer review only

For peer review only - http://bmjopen.bmj.com/site/about/guidelines.xhtml

This study aimed to explore patients' and healthcare practitioners' views on i) non-adherence to hypertension medication and ii) a complex intervention designed to support medication adherence. Initial ideas for the intervention consisted of a very brief face-to-face discussion with a primary care provider, followed by ongoing support via a DI (SMS messages or smartphone app). Feedback from participants included preferred content of the intervention and factors likely to influence engagement.

Methods

This study is reported in line with the consolidated criteria for reporting qualitative research studies checklist (COREQ)[22], see supplementary file 1.

Design

We undertook a qualitative study using semi-structured interviews followed by focus groups.

Recruitment and sampling

Patients were recruited for interviews from primary care practices based in the East of England (n=3) and East London (n=1). Practices were identified with the help of the Clinical Research Network, an organisation which supports the delivery of research within NHS practices in England. Patients were eligible to participate if they were: 1) prescribed at least one antihypertensive medication for at least the previous three months 2) deemed non-adherent according to GP practice records, with a blood pressure reading of over 140/90 mmHg and/or gaps in filling repeat prescriptions in the previous three months and 3) used either SMS or smartphone

For peer review only - http://bmjopen.bmj.com/site/about/guidelines.xhtml

apps. The practice administrator at each site generated a list of prospective participants that met criteria 1 and 2, which was screened by a GP or Practice Nurse. Eligible patients received a study pack from their GP practice in the post consisting of an invitation letter and participant information sheet. Posters highlighting the study were also displayed in the GP practices. Patients interested in taking part were invited to contact the researcher (MVE) via telephone or email; at which point the researcher checked that all three eligibility criteria were met before scheduling an interview.

A convenience sample of healthcare practitioners were recruited from the four GP practices taking part in this study. Healthcare practitioners were eligible to be interviewed if they were involved in the care of patients with hypertension e.g. through medication reviews (conducted by a GP, Practice Nurse or Practice Pharmacist) or blood pressure checks and/or health assessments (conducted by a Healthcare Assistant). The researcher invited healthcare practitioners to participate during the face-to-face study set-up meeting where they were given a study information pack. The researcher contacted the healthcare practitioners one week later to check willingness to participate and to schedule interviews for those who were interested.

Recruitment for focus groups followed that of the patient interviews. To address the low response from eligible patients, the eligibility criteria was widened to include patients prescribed medication for type 2 diabetes, as research indicates similar rates of medication non-adherence and barriers to adherence as for hypertension.[23, 24] The eligibility criteria was also narrowed to ensure that participants were familiar with using smartphone apps (i.e. SMS alone was not sufficient). The decision to cease individual interviews and switch to focus groups

For peer review only

with patients was due to preliminary analysis from the interviews adding little new information to findings from previous research, and our experience of the usefulness of focus groups to gain feedback on the format, content and structure of DIs. [25–27]

Data collection

Patient interviews were conducted by one researcher (MVE) at patients' home, workplace or local library. Healthcare practitioner interviews were conducted at their place of work by the same researcher (MVE). Focus groups were conducted at community centres local to the patients' general practice and moderated by two researchers (MVE and JJ).

Interviews and focus groups were guided by flexible topic guides [28] developed by the research team, drawing on the Perceptions and Practicalities Approach (PAPA) framework [18] and previous research experience in both the topic area and intervention development. Topic guides were reviewed by Patient and Public Involvement (PPI) representatives to ensure the questions were easy to understand and appropriate for the study objectives. Broadly, interview topics included: reasons for medication non-adherence, current practice of HCPs during medication-related consultations, and views on a potential SMS text message or smartphone app intervention that could support adherence. Example intervention content included medication reminders, advice and support messages, and feedback on adherence. See supplementary file 2 for the topic guides and example DI content. HCPs were shown an example protocol for a very brief face-to-face discussion or 'VBI' to generate discussion (see supplementary file 3). Components of the VBI included: introducing the digital support to the patient and inputting basic patient information via an online questionnaire to generate the tailored digital support.

For peer review only - http://bmjopen.bmj.com/site/about/guidelines.xhtml

Focus groups followed similar topic guides to the interviews, focusing on attitudes towards smartphone apps in particular, including the acceptability of sensing technology such as location sensing. To prompt discussion and gain feedback, both interview and focus group participants viewed stimulus materials of example intervention messages, including medication reminders, and smartphone app features, including graphs and images (see Figure 1 for examples).

Written informed consent was taken in person by the researcher immediately prior to the interviews and focus groups commencing. All patients received a £20 voucher for taking part. Interviews and focus groups were audio-recorded and professionally transcribed verbatim. Interviews lasted on average 47 minutes and focus groups 1 hour and 28 minutes.

Data Analysis

Analysis was informed methodologically by the constant comparative approach[29] and theoretically by the Perceptions and Practicalities Approach (PAPA), which incorporates and acknowledges the blurring of and distinction between intentional and non-intentional non-adherence.[18] Interview transcripts were read and re-read to aid familiarisation and identify preliminary themes; these broad descriptive themes were formed into an initial coding framework related to barriers and facilitators to medication adherence and a potential intervention. Each transcript was then coded systematically (MVE) using NVivo qualitative data-indexing software (version 12; QSR International) and the coding framework was refined throughout the process. The process was repeated for focus group transcripts; the coding framework was further expanded and refined, given the additional topics explored in the focus groups. A sample of interview and focus group

For peer review only

transcripts were independently coded by a second researcher (JJ) to confirm and strengthen the validity of findings. Meetings between the research team (MVE, JJ, HE) facilitated data analysis including discussion of themes, sub-themes and the interrelationships.

Patient and public involvement

All study materials (participant information sheet, invitation letter, study poster, consent form, topic guides and stimulus materials) were reviewed by representatives from the Cambridge University Hospitals Patient and Public Involvement (PPI) panel. We made a number of changes to the study materials as a result of PPI input: adjusted the language to make the documents more accessible and ensured interview questions were sensitively worded and easy to understand from a patient perspective. PPI representative Jennifer Bostock provided input throughout the study and reviewed and commented on this manuscript.

Ethical approval

Ethical approval for this study was obtained from the West Midlands – Solihull Research Ethics Committee, as part of NHS Health Research Authority approvals (Reference: 18/WM/0050).

Results

Of the 126 eligible patients prescribed medication for hypertension who were sent an invitation, six were interviewed. All 11 healthcare practitioners approached by the researcher were deemed eligible and agreed to take part. Of the 218 patients prescribed medication for hypertension and/or type 2 diabetes who were then sent

For peer review only

an invitation to a focus group, 14 participated (four focus groups with 3-5 patients per group). Recruitment of participants to focus groups continued until no new themes were emerging in relation to the specific topics covered.

Patient participant characteristics are reported in Table 1. Their mean age was 62.7 years (range 47 – 79 years), 60% identified as male and 85% as White British. Eighty percent of patients reported using both SMS and smartphone apps, with the remaining 20% using SMS text messages only. All patients self-reported having occasionally missed or skipped their medication in the previous 3 months. HCP participant characteristics are reported in Table 2; six Practice Nurses, two Healthcare Assistants, two Practice Pharmacists, one GP. Participants were recruited from four GP practices based in urban (n=3) and rural (n=1) locations. GP practice Index of Multiple Deprivation (IMD) scores, a measure of relative socio-economic status in England based on postcode, ranged from 'least deprived' (n=2), to 'less deprived' (n=1) and 'more deprived' (n=1), see Table 2.

Table 1 Participant characteristics (patients)

Characteristics	(n)	%
**Gender**		
Female	8	40
Male	12	60
**Age (years)**		
41-50	2	10
51-60	4	20
61-70	11	55
71-80	3	15
**Ethnicity**		
Asian or Asian British – Indian	1	5
Asian or Asian British – Pakistani	1	5
Black or Black British – Caribbean	1	5
White British	17	85

For peer review only - http://bmjopen.bmj.com/site/about/guidelines.xhtml

For peer review only

**Phone use**		
SMS only	4	20
SMS and smartphone app	16	80
**Data collection method**		
Semi-structured interview	6	30
Focus group	14	70

Note: n=20

Table 2 Participant characteristics (healthcare practitioners) and GP practice demographics

**Characteristics**	**(n)**	**%**
**Healthcare practitioners**		
**Job role**		
General Practitioner	1	9
Healthcare Assistant	2	18
Practice Nurse	6	55
Practice Pharmacist	2	18
**Gender**		
Female	10	91
Male	1	9
**Years practising**		
≤10	5	46
11-20	2	18
21-30	2	18
≥31	2	18
**GP practices**		
**GP practice location**		
Urban	3	75
Rural	1	25
**GP practice IMD quintile**		
First quintile (least deprived)	2	50
Second quintile (less deprived)	1	25
Fourth quintile (more deprived)	1	25

Note: Healthcare practitioner n=11, GP practice n=4, IMD = Index of Multiple Deprivation, which ranks every small area in England from 1 ('most deprived area') to 32,844 ('least deprived'). IMD scores of the 4 GP practices in this study: 9924, 25270, 26871, 30807.

For peer review only

To present the findings, we broadly categorise the key themes identified into the following categories: reasons for non-adherence, recommendations for message content, tailoring the digital intervention, acceptability of sensing technology, and attitudes towards a VBI. We provide illustrative quotes below. See supplementary file 4 for additional quotes from participants. For reference, 'digital intervention' (DI) refers to both SMS text messages and smartphone app, as the same intervention messages can be delivered using both formats.

Reasons for non-adherence

Participants provided two key explanations of non-adherence to antihypertensive medication. First, for *non-intentional* non-adherence, forgetting was the most common reported reason and was mentioned in three ways: forgetting to take medication, forgetting whether or not medication had been taken and forgetting to re-order the prescription in time.

> *Sometimes you can't remember whether you have taken them already. And that can be problematic, so if someone asks you, you think, 'well, I don't know, maybe I have, maybe that was yesterday.' [P04, male, 40s]*

Second, in terms of *intentional* non-adherence, the experience or anticipation of side effects was a reason given for skipping, altering or delaying medication, as was the general reluctance to be reliant on medication.

> *I wish I could live without medication, I hate pumping my body with drugs. Sometimes I wonder, 'what side effects am I gonna have with this? Is it really benefitting me?' [P06, female, 60s]*

> *A lot of patients […have said], 'yes, the doctor has changed my medication, but they make me go funny, so I'm just going to take half or I'll just crush that and just take half instead of the two.' [HCP 05, Healthcare Assistant, female]*

These overarching explanations were apparent when participants discussed the merits of a digital intervention to improve adherence, as presented in the following sections. We begin with participants' views about a DI's messaging content, followed

For peer review only - http://bmjopen.bmj.com/site/about/guidelines.xhtml

by tailoring and then sensing technology; the final section considers the role of the VBI component.

Recommendations for message content

Simple reminder messages were perceived as useful for both taking medication and re-ordering prescriptions.

> *Even if I'm in a hurry, [when] I receive this reminder I [would] realise the importance. I think if I keep getting messages that would be very effective and definitely help me. Even if I'm tired and it would make me […] I'd force myself to get up and go and take the medication. [P06, female, 60s]*

> *It would be useful, if you're running out of tablets, to have some way of automatically reordering or a reminder to do that. So it's reminding you to take your tablets, and also when you're running low. [P04, male, 40s]*

Information-giving messages were only perceived as helpful by participants if providing advice when medication had been missed, for example the safest way to 'catch up' on a missed dose.

> *There ought to be a button of 'I've forgotten them 'til now, which bits should I take?' That could be useful. [FG3, male]*

While HCP-participants recommended messages about the benefits of medication or the consequences of non-adherence, patient-participants considered these unhelpful and unnecessary, particularly if lack of knowledge was not a barrier to adherence.

> *I know what the risk is [from not taking my medication]. I don't feel that I want it repeated, no. [P03, male, 60s]*

There was, however, some recognition that newly-diagnosed patients may find such information motivating:

> *If you're new to taking blood pressure tablets [information on consequences of non-adherence] would be good. I mean, us experienced people who've taken them for years most probably don't need reminding that if you don't take it, something serious is gonna happen to you. [FG3, male]*

For peer review only - http://bmjopen.bmj.com/site/about/guidelines.xhtml

The idea of receiving feedback on one's adherence, generated from self-report via SMS message or App, in a message of encouragement (e.g. *'Well done!')* was viewed as unnecessary. Participants were more receptive to schematic feedback in the form of a graph, score or percentage.

> *Some people might need that encouragement, but then again, it sounds a bit patronising to some people, doesn't it [laughs]? […] I think the percentage thing would give people pride, you know, 'oh, I've reached 100% [of taking my medication] this month, I feel really good about that'. Once a month I'd like to know what my score was for the month. I think that would probably be enough incentive for me personally. [P03, male, 60s]*

Regular smartphone users suggested that feedback in the form of a monthly calendar highlighting 'missed medication' days, could be useful for spotting a pattern and identifying the circumstances of those days that contributed to a missed dose. Moreover, participants suggested the potential for this to facilitate discussion with a healthcare professional too:

> *A: I think [the app] would also be good to take, when you have a medication review, to take to your GP so he or she can see what's going on as well.*
> *B: Yeah, help fix it by doing something, yeah*
> *[FG3, male (A, B)]*

Tailoring the digital intervention

Participants commented how they would be more likely to use, and continue to use, the DI if the messages were tailored to their preferences and their individual medication regime, in terms of frequency and timing of doses:

> *Some people are on medication once a day, twice a day, three, four. Could the app be tailor-made for the individual? And remind us accordingly? [FG2, female]*

> *A: That's why [the intervention] should be tailor-made for the individual patient. I think it's going to be critical really. Rather than a generic –*
> *B: And have options, yeah.*
> *A: Because if it's a generic app and it doesn't suit some people they won't use it or they won't respond to it. [FG1, male (A,B)]*

For peer review only - http://bmjopen.bmj.com/site/about/guidelines.xhtml

Participants noted the importance of the DI including *all* their prescribed medications, i.e. not just the hypertension ones.

> *I think it would need to be somewhat of a select or deselect, you know, 'take all' but you can un-tick the ones that you're not taking now. [FG4, female]*

> *A: I would do it as all one. Even if you're doing it principally motivated by blood pressure, in the sense it's, you're trying to remind us to take pills in general, aren't you, so you have to somehow-*
> *B: Yes, I think you want all of them there. [FG3, male (A), female (B)]*

To ensure that tailoring meets patients' preferences and medication regime, and the changes over time, participants highlighted the importance of patients having control over the DI's settings. For example, being able to change timings of reminders and adding in short term medication.

> *A: I think I'd like to put my own [medications] in. And then when you have a "short course" [of medication] as we say, I'll add that in as well. I'd rather be in charge of putting it in.*
> *B: Especially as some you have to have on an empty stomach, don't you?*
> *A: Yeah, so you could fiddle with your timings for that one. [FG2, female (A), male (B)]*

> *It's gotta be a dynamic thing. Medications change, dosages change, things get stopped, times may change, so I probably would see as an app which patients would be free to add and subtract. [HCP 02, GP, male]*

A 'snooze' function (similar to an alarm snooze) was well-received by participants, provided users could set their own parameters, e.g., length of snooze duration and maximum number of snoozes.

> *It would be good for me 'cos I'm often not home when I'm supposed to take them, so if you hit the 'snooze' for an hour or whatever you choose it to be, […]and it'll keep reminding me again and I'll take the tablets [when I'm home]. [FG1, male]*

For peer review only - http://bmjopen.bmj.com/site/about/guidelines.xhtml

A suggestion for tailoring by adding images of medications into the app raised more problems than benefits; participants pointed out that *"every time you get the medication, the box changes" [FG1]* and it was felt this would create confusion, rather than help.

Acceptability of sensing technology

Participants were initially wary about the incorporation of sensing technology, such as GPS or Wi-Fi to determine location, into an app. They raised concerns about surveillance, typically referred to as "*Big Brother*" *[FG1, FG3]* watching them. Participants were more likely to accept sensing technology if the perceived benefits (such as tailoring medication reminders to their specific schedule and locations) outweighed concerns about data privacy.

> *It would make it impossible to forget 'cos I'd just walk through the door and take 'em. That would be brilliant. [FG1, male]*

Participants requested information to address these concerns, including: who has access to their data beyond the university (in particular, less trusted organisations such as insurance or marketing companies), where data are stored, and what happens in the event of hacking.

> *A: Who are you gonna share this with? That's all I'm worried about […]*
> *B: It could be pretty valuable information for insurance companies to put their premiums up. [FG4, male (A), female (B)]*

> *I think it'd be more reassuring to know it was a medical body behind it or a university body behind it; it gives it some substance and credibility. [FG1, male]*

Participants wanted to retain personal control over the sensing function, with the ability to choose when the app tracks and records their location data as well as the

For peer review only - http://bmjopen.bmj.com/site/about/guidelines.xhtml

ability to opt in/out at any point.

I think it would be a case of opt-in because I think some people would think it an invasion of privacy. I mean, personally I think it's a good idea but, you see, some people wouldn't like it. [FG4, female]

Discussions about sensing technology prompted participants to suggest further ideas for functions of an app. Participants in two focus groups suggested linking the sensing technology with the smartphone calendar, to proactively detect periods when away from home, triggering reminder messages to pack medication or re-order prescriptions.

The app ought to be able to detect [that] my calendar says, 'Away for the weekend.' So the app could say, "Ooh look, you're going away for the weekend" it could send me a message or something on the Friday to make sure I pack them. That's almost what I want to be reminded of. [FG3, male]

Participants emphasised the need for additional features to be optional, recognising that over-complicating the DI risked disengagement from potential users.

I suppose it's a case though of getting sufficient ability to customise it against making it just too longwinded and complicated for people to be bothered. [FG4, female]

I'm just trying to think of just the least steps possible for the patient, because just adding more things is going to make them less likely to use these sorts of things… It needs to just be easy for them. [HCP 01, Practice Pharmacist, female]

Above all, participants emphasised the importance of the DI being user-friendly for the target group, many of whom may be less familiar with smartphones.

The caveat I suppose might be that those that tend to have the chronic diseases tend to be the older age group so they may not be so tech savvy. We've got some patients who don't use mobile phones even now. [HCP 02, GP, male]

Attitudes towards a very brief face-to-face intervention (VBI)

For peer review only - http://bmjopen.bmj.com/site/about/guidelines.xhtml

Patient-participants' discussions about the DI functions largely focused on addressing non-intentional non-adherence - mainly forgetting. On the whole, they were sceptical about a DI's success in addressing intentional non-adherence:

> *If they're not taking the tablets and they don't wanna take the tablets, why would they sign up for the app? [FG1, male].*

HCP-participants suggested including messages encouraging users to contact their healthcare provider if experiencing problems with their medication.

> *That would be really useful in that if they're stopping it for any reason it needs to come up with a message to say, "Please make an appointment with your GP. There may be alternative medications available which would suit you and you need to make an appointment to discuss that". [HCP 03, Practice Nurse, female]*

However, a more promising way of addressing intentional non-adherence was highlighted in relation to the 5-minute VBI component prior to use of the DI. The VBI was presented as a way for HCPs to signpost patients to the DI and discuss medication taking behaviour. HCPs talked positively about how, if done in a non-judgemental way and by an HCP with an established rapport with the patient, this could foster open communication and a more constructive consultation.

> *That's the important thing, when patients can relate to you and they can see that you're actually not judging them, they do tend to then engage better. [HCP 07, Practice Nurse, female]*

A key aspect of encouraging honest communication in the VBI would be acknowledging that it is acceptable to have concerns about being prescribed medication. HCPs recommended asking the patient to talk through these concerns and, if needed, book a follow-up consultation with a prescribing practitioner about changing medication.

> *Have a discussion with them as to what's been happening, what the issues are, how we can make it easier for them […] 'Is there a problem with it? Are you getting side-effects? Do you find it difficult to take?' And then we can explore some of the issues. What is really important is to sift through what the issues*

For peer review only - http://bmjopen.bmj.com/site/about/guidelines.xhtml

> *are. Our role in the face-to-face is actually we can explore some of these things a bit easier. [HCP 06, Practice Nurse, female]*

All HCPs perceived the VBI element as feasible to deliver within primary care, recommending incorporating it alongside a medication review or blood pressure check. HCPs had two key provisos: training to help them deliver the VBI within the tight timing of five minutes, and a "*user-friendly*" template that could be incorporated in existing computer systems for inputting patient data to inform the subsequent DI. HCPs also noted the need for sufficient training in using the DI itself, given their role in encouraging its use in their patients following the VBI.

> *I think that will be important, that whoever is talking about the app needs to know how it works and how you use it… Because if somebody who is recommending it doesn't know how to use it then you're not gonna buy into it. [HCP 09, Practice Nurse, female]*

For peer review only

Discussion

Summary of main findings

Patients prescribed antihypertensive medication and the healthcare practitioners that care for them, highlighted nonintentional (forgetting) and intentional (side-effects, reluctance to medicate) reasons for their non-adherence. Participants found a mobile digital intervention (DI) that provided simple medication reminders and feedback messages acceptable. To facilitate engagement with the DI, participants recommended it was tailored to the needs of the individual and their medication regime as well as providing user control over the tailoring and other optional functions. The use of sensing technology within a smartphone app was acceptable to participants provided they received comprehensive information about the associated use and confidentiality of their data.

While the DI was considered limited in its potential to address intentional non-adherence, HCPs saw the potential for a brief face-to-face discussion (or 'VBI') with patients in addressing this gap, when delivered alongside a DI. Incorporating a VBI into a routine primary care was considered feasible, if it could be integrated into existing practice software systems and if training were provided.

Strengths and limitations of the study

Drawing on relevant theory, [8, 9, 18] this study was conducted as development work with a target patient group to inform aspects of an intervention as part of a larger research programme.[30] Whilst previous research has investigated the use of sensing technology and smartphone apps for health, [19] this study is among the first to gather qualitative data on the acceptability of such technology (e.g.

For peer review only - http://bmjopen.bmj.com/site/about/guidelines.xhtml

Wi-Fi or GPS) in a smartphone app designed to support medication adherence (see also Kassavou and colleagues [31]). Whilst advances in technology can provide additional features to smartphone apps, it is important to assess the intended user group's views of such technology before its implementation.[32]

We gained insights from a range of healthcare practitioners on the acceptability and feasibility of incorporating a VBI for medication adherence into a primary care consultation, a topic that has not been previously explored in-depth. The recommendations arising from our findings can inform the development and implementation of a medication adherence VBI in primary care. Developers should consider the following: the importance of the practitioner-patient relationship when discussing medications, exploration of patient-specific barriers to adherence, templates embedded within existing GP practice systems, and sufficient training for HCPs.

The use of stimulus materials generated discussion in the interviews and focus groups, and provided focused responses for specific hypothetical intervention components.

We acknowledge that this is a small-scale qualitative study, where 85% of the patient sample were White British and 91% of the healthcare practitioner sample were female. As such, the findings may be limited in their application to a patient and healthcare professional population. However, the depth and focus of insights gained provided rich data that were sufficiently useful in informing the development and refinement of intervention components for the wider programme, and to similar interventions.

For peer review only - http://bmjopen.bmj.com/site/about/guidelines.xhtml

We experienced challenges with recruiting patients through GP practices, particularly those who were non-adherent to their medication, a group who may be less likely to participate in a study of this nature. For future studies we would recommend widening recruitment methods to include patients not tied to a specific sample of GP practices, e.g. via social media channels or community groups. We acknowledge the possibility that patients who are intentionally nonadherent to their medication may be unwilling to download an adherence app or receive SMS support messages. In these instances, alternative, more intensive intervention methods involving multiple BCT components may be considered appropriate, such as motivational interviewing delivered face-to-face and/or over the telephone.[33–35]

Comparisons with existing literature

The findings echo previous research that has identified the main reasons for non-adherence to cardiovascular-related medication as forgetting and side-effects,[24] [36] as well as the broad categorisation of reasons into intentional and non-intentional.[37, 38] In our study, this distinction was particularly helpful when considering which elements of an intervention were appropriate for targeting these two broad categories.

Participants with lived experience of hypertension saw little value in information-style messages (e.g. about the consequences of non-adherence) in addressing intentional non-adherence. Rather, they suggested that such messages may be most helpful for newly diagnosed patients. This follows previous qualitative research in which mHealth interventions were deemed especially appropriate for “newbies” [39] i.e. patients with less experience in managing a health condition

For peer review only - http://bmjopen.bmj.com/site/about/guidelines.xhtml

compared to those with established medication routines, for atrial fibrillation [40] and type 2 diabetes. [39] Similar to previous studies [26], participants expressed concerns about receiving too many messages, suggesting this would influence (dis)engagement with the DI. Participants also emphasised the need for a DI to be as simple and easy-to-use as possible, another common theme in usability studies for medication adherence DIs, whereby difficulties with navigating a website, SMS or smartphone app have presented barriers to usage.[41, 42] A related concern is the potential burden that self-monitoring DIs place on the user, for example, asking patients to self-report their medication taking behaviour within a set timeframe.[43, 44] Our findings support the need for usability testing with the DI target users, which could include assessing any associated burdens or extra responsibilities placed on the user.

Participants in this study saw the benefit that sensing technology could provide but raised data privacy concerns about its use within a medication reminder smartphone app, requesting comprehensive information and user control. Similar concerns have been identified in previous research into location-sensing apps. For individuals living with HIV, the acceptability of location-based self-monitoring reminders was dependent on the purpose of the app and who would have access to their data [45]. Similarly, young adults in Dennison [46] worried about the storage of personal location data collected by health apps and wanted control over personalising the app settings.

Despite the privacy concerns, participants in this study viewed a location-sensing smartphone app more favourably if it was created by a university or charity rather than a commercial company. This follows user feedback of other location-based apps for smoking cessation, [47] medication adherence [31] and mental

For peer review only - http://bmjopen.bmj.com/site/about/guidelines.xhtml

health, [48] in which apps designed by universities or for research purposes were deemed more trustworthy by participants. This reflects the discourse around the ethics of mHealth, whereby third parties and insurance companies pose potential threats to the safety of patients' health data collected by sensors or smartphone apps.[43] These ethical considerations are of particular importance given the rise of mHealth in the healthcare sphere.

Recommendations for an intervention to support medication adherence

The findings from this study have several implications for the development of a DI to support medication adherence. To encourage engagement with an intervention, it needs to be highly tailored to each individual. This includes: the timing and content of reminder messages (to address non-intentional non-adherence) and the content of support messages (for intentional non-adherence), where knowledge and duration of health condition varies between individuals. Furthermore, a key tailoring variable as recommended by HCP-participants was the individual patient's specific barrier(s) to adherence. Tailoring data can be collected using various methods, ideally before the start of the intervention for optimal impact. This could include a short questionnaire, in person or by phone with a practitioner, within a smartphone app, or via a set of SMS messages requiring responses.[26]

It was common for participants in this study to be taking multiple medications per day, and most wanted this to be reflected in the medication reminders. This requires a balance between providing appropriate adherence support without over-complicating the DI or overburdening the user, resulting in reduced intervention engagement.[49]

For peer review only

This study obtained novel insight from patients on the use of passive sensing technology within a medication adherence smartphone app. To increase the acceptability of sensing technology, future apps should explain the benefits that it can provide to the user, such as tailored medication reminders based on real-time location, or prompts to pack medication for upcoming holidays detected via calendar syncing. The app must provide a flexible opt in/out option for the collection of sensing data as well as information on how personal data will be used and stored within the app. Lastly, users may be more accepting of a location-sensing app created by a university or charity rather than a commercial company.,

Primary care was viewed as an appropriate setting for HCPs to introduce patients to a DI and pair it with a brief behavioural face-to-face discussion, or 'VBI'. More specifically, this could address intentional non-adherence by exploring the specific barriers to medication adherence with patients. Using a non-judgemental approach for this, would encourage patients' openness, which in turn would provide more useful information for tailoring the DI and possibly making adjustments to the all patient's regime as part of the usual care. This supports a body of literature on shared decision-making [50] which has demonstrated an association between an improved patient-professional partnership and medication adherence, for a variety of conditions [51] and for hypertension specifically.[50, 52] Delivering a VBI requires skill, in order to incorporate all elements and within the short timeframe.[53, 54] Our findings indicate the importance of comprehensive training for healthcare professionals which incorporate the principles of shared decision-making and the skills to deliver the intervention in under five minutes, as well as proficiency in using a DI. Lastly, the template for HCPs to complete the VBI and/or enter patients' details

For peer review only - http://bmjopen.bmj.com/site/about/guidelines.xhtml

into the DI should be user-friendly and embedded into existing GP practice software systems.

Conclusion

Overall, patients and healthcare practitioners saw the benefit of receiving medication reminders via SMS message or smartphone app. Intervention developers should consider an intervention that is highly tailored to the user, straightforward to use, and addresses data privacy concerns. The use of sensing technology in an adherence support smartphone app was acceptable to patients, therefore future research could investigate the feasibility of incorporating such technology into a smartphone app for adherence. A routine primary care consultation was viewed as an appropriate setting to introduce the DI to patients and discuss medication-taking behaviour with patients, but the feasibility of delivering it as ‘very brief’ i.e. under five minutes, should be explored further.

Supplementary material

S1 – COREQ checklist

S2 – Topic guides and sample of proposed intervention content

S3 – Example VBI protocol

S4 – Extra participant quotations

Figure 1 (legend)

For peer review only

Example digital intervention content to generate discussion in interviews and focus groups; medication reminder notification, feedback on medication adherence levels (weekly and monthly), SMS support message

Acknowledgements

This study was conducted on behalf of the Programme on Adherence to Medication team (see https://www.phpc.cam.ac.uk/pcu/research/research-projects-list/other-projects/pam/ for team members). We acknowledge and thank the following individuals for contributing to the development of the topic guides and visual prompts for this study: Jagmohan Chauhan and Sandra Servia, Department of Computer Science and Technology, University of Cambridge; Debi Bhattacharya, School of Pharmacy, University of East Anglia.

We thank the patients, healthcare practitioners, practice managers and administrative staff at the 4 GP practices who took part in this study. We are grateful to Patient and Public Involvement (PPI) representatives for reviewing the design and ethics of this study. We thank PPI representative Jennifer Bostock for their input across the whole study and for reviewing the manuscript. We acknowledge the support of the National Institute for Health Research Clinical Research Network (NIHR CRN).

Author contributions

All authors made substantial contributions to the conception and design of the study. MVE conducted the interviews, co-facilitated the focus groups, conducted data analysis and drafted the manuscript. JJ co-facilitated the focus groups, contributed to data analysis and drafted the manuscript. HE provided supervision and guidance for

For peer review only - http://bmjopen.bmj.com/site/about/guidelines.xhtml

data collection, contributed to data analysis and drafted the manuscript. SS, FN, WH, AK and CA critically revised the manuscript and provided intellectual input and expert advice. All authors have read and approved this manuscript.

Funding

This article presents independent research funded by the National Institute for Health Research (NIHR) under the Programme Grants for Applied Research programme [RP-PG-0615-20013]. The views expressed in this publication are those of the author(s) and not necessarily those of the NIHR or the Department of Health and Social Care.

Competing interests

None declared.

Ethics approval

This study was approved by the West Midlands – Solihull Research Ethics Committee, as part of NHS Health Research Authority approvals (Reference: 18/WM/0050).

Data availability statement

Data are available upon reasonable request from the corresponding author.

Abbreviations: COREQ, consolidated criteria for reporting qualitative research; DI, digital intervention; GP, general practitioner; HCP, healthcare practitioner; IMD, Index of Multiple Deprivation; NHS, National Health Service; NIHR CRN, National Institute of Health Research Clinical Research Network; VBI, very brief intervention.

For peer review only

For peer review only - http://bmjopen.bmj.com/site/about/guidelines.xhtml

References

[1] Sabaté E, World Health Organization (eds). *Adherence to long-term therapies: evidence for action*. Geneva: World Health Organization, 2003.

[2] Chowdhury R, Khan H, Heydon E, et al. Adherence to cardiovascular therapy: a meta-analysis of prevalence and clinical consequences. *Eur Heart J* 2013; 34: 2940–2948.

[3] Gupta Pankaj, Patel Prashanth, Štrauch Branislav, et al. Risk Factors for Nonadherence to Antihypertensive Treatment. *Hypertension* 2017; 69: 1113–1120.

[4] Forouzanfar MH, Liu P, Roth GA, et al. Global Burden of Hypertension and Systolic Blood Pressure of at Least 110 to 115 mm Hg, 1990-2015. *JAMA* 2017; 317: 165–182.

[5] Mills KT, Stefanescu A, He J. The global epidemiology of hypertension. *Nat Rev Nephrol* 2020; 16: 223–237.

[6] Gakidou E, Afshin A, Abajobir AA, et al. Global, regional, and national comparative risk assessment of 84 behavioural, environmental and occupational, and metabolic risks or clusters of risks, 1990–2016: a systematic analysis for the Global Burden of Disease Study 2016. *The Lancet* 2017; 390: 1345–1422.

[7] Brown MT, Bussell JK. Medication Adherence: WHO Cares? *Mayo Clin Proc* 2011; 86: 304–314.

[8] Horne R, Weinman J, Barber N, et al. Concordance, adherence and compliance in medicine taking.

[9] Clifford S, Barber N, Horne R. Understanding different beliefs held by adherers, unintentional nonadherers, and intentional nonadherers: application of the Necessity-Concerns Framework. *J Psychosom Res* 2008; 64: 41–46.

[10] Choudrie J, Pheeraphuttranghkoon S, Davari S. The Digital Divide and Older Adult Population Adoption, Use and Diffusion of Mobile Phones: a Quantitative Study. *Inf Syst Front* 2020; 22: 673–695.

[11] Baird B, Charles A, Honeyman M, et al. Understanding pressures in general practice. 100.

For peer review only

For peer review only - http://bmjopen.bmj.com/site/about/guidelines.xhtml

[12] King R, Ryan T, Senek M, et al. The impact of COVID-19 on work, training and well-being experiences of nursing associates in England: A cross-sectional survey. *Nurs Open*; n/a. DOI: 10.1002/nop2.928.

[13] Thakkar J, Kurup R, Laba T-L, et al. Mobile Telephone Text Messaging for Medication Adherence in Chronic Disease: A Meta-analysis. *JAMA Intern Med* 2016; 176: 340–349.

[14] Armitage LC, Kassavou A, Sutton S. Do mobile device apps designed to support medication adherence demonstrate efficacy? A systematic review of randomised controlled trials, with meta-analysis. *BMJ Open* 2020; 10: e032045.

[15] McLean G, Band R, Saunderson K, et al. Digital interventions to promote self-management in adults with hypertension systematic review and meta-analysis. *J Hypertens* 2016; 34: 600–612.

[16] Xiong S, Berkhouse H, Schooler M, et al. Effectiveness of mHealth Interventions in Improving Medication Adherence Among People with Hypertension: a Systematic Review. *Curr Hypertens Rep* 2018; 20: 86.

[17] Hardeman W, Houghton J, Lane K, et al. A systematic review of just-in-time adaptive interventions (JITAIs) to promote physical activity. *Int J Behav Nutr Phys Act* 2019; 16: 31.

[18] Horne R, Cooper V, Wileman V, et al. Supporting Adherence to Medicines for Long-Term Conditions. *Eur Psychol* 2019; 24: 82–96.

[19] Cornet VP, Holden RJ. Systematic review of smartphone-based passive sensing for health and wellbeing. *J Biomed Inform* 2018; 77: 120–132.

[20] Pouls BPH, Vriezekolk JE, Bekker CL, et al. Effect of Interactive eHealth Interventions on Improving Medication Adherence in Adults With Long-Term Medication: Systematic Review. *J Med Internet Res* 2021; 23: e18901.

[21] Lu X, Yang H, Xia X, et al. Interactive Mobile Health Intervention and Blood Pressure Management in Adults. *Hypertension* 2019; 74: 697–704.

[22] Tong A, Sainsbury P, Craig J. Consolidated criteria for reporting qualitative research (COREQ): a 32-item checklist for interviews and focus groups. *Int J Qual Health Care* 2007; 19: 349–357.

[23] Cramer JA, Benedict Á, Muszbek N, et al. The significance of compliance and persistence in the treatment of diabetes, hypertension and dyslipidaemia: a review. *Int J Clin Pract* 2008; 62: 76–87.

[24] Kassavou A, Sutton S. Reasons for non-adherence to cardiometabolic medications, and acceptability of an interactive voice response intervention in patients with hypertension and type 2 diabetes in primary care: a qualitative study. *BMJ Open* 2017; 7: e015597.

[25] Jamison J, Naughton F, Gilbert H, et al. Delivering Smoking Cessation Support by Mobile Phone Text Message: What Information do Smokers Want? A Focus Group Study. *J Appl Biobehav Res* 2013; 18: 1–23.

[26] Morton K, Sutton S, Hardeman W, et al. A Text-Messaging and Pedometer Program to Promote Physical Activity in People at High Risk of Type 2 Diabetes: The Development of the PROPELS Follow-On Support Program. *JMIR MHealth UHealth* 2015; 3: e105.

For peer review only

[27] Eborall H, Morton K. Use of Focus Groups in Developing Behavioural mHealth Interventions: A Critical Review. In: Barbour RS, Morgan DL (eds) *A New Era in Focus Group Research: Challenges, Innovation and Practice*. London: Palgrave Macmillan UK, pp. 109–127.

[28] Britten N. Qualitative Research: Qualitative interviews in medical research. *BMJ* 1995; 311: 251–253.

[29] Charmaz K. *Constructing Grounded Theory: A Practical Guide through Qualitative Analysis*. SAGE, 2006.

[30] PAM - Programme on Adherence to Medication. *Primary Care Unit*, https://www.phpc.cam.ac.uk/pcu/research/research-groups/bsg/research-themes/medication-adherence/pam-programme-on-adherence-to-medication/ (accessed 1 October 2020).

[31] Kassavou A, A'Court CE, Chauhan J, et al. Assessing the acceptability of a text messaging service and smartphone app to support patient adherence to medications prescribed for high blood pressure: a pilot study. *Pilot Feasibility Stud* 2020; 6: 134.

[32] National Institute for Health and Care Excellence. Evidence Standards Framework for Digital Health Technologies., https://www.nice.org.uk/Media/Default/About/what-we-do/our-programmes/evidence-standards-framework/digital-evidence-standards-framework.pdf (2019, accessed 16 July 2020).

[33] Palacio A, Garay D, Langer B, et al. Motivational Interviewing Improves Medication Adherence: a Systematic Review and Meta-analysis. *J Gen Intern Med* 2016; 31: 929–940.

[34] Aubeeluck E, Al-Arkee S, Finlay K, et al. The impact of pharmacy care and motivational interviewing on improving medication adherence in patients with cardiovascular diseases: A systematic review of randomised controlled trials. *Int J Clin Pract* 2021; 75: e14457.

[35] Zomahoun HTV, Guénette L, Grégoire J-P, et al. Effectiveness of motivational interviewing interventions on medication adherence in adults with chronic diseases: a systematic review and meta-analysis. *Int J Epidemiol* 2017; 46: 589–602.

[36] Leon N, Surender R, Bobrow K, et al. Improving treatment adherence for blood pressure lowering via mobile phone SMS-messages in South Africa: a qualitative evaluation of the SMS-text Adherence SuppoRt (StAR) trial. *BMC Fam Pract* 2015; 16: 80.

[37] Barber N. Should we consider non-compliance a medical error? *Qual Saf Health Care* 2002; 11: 81–84.

[38] Horne R, Chapman SCE, Parham R, et al. Understanding Patients' Adherence-Related Beliefs about Medicines Prescribed for Long-Term Conditions: A Meta-Analytic Review of the Necessity-Concerns Framework. *PLOS ONE* 2013; 8: e80633.

[39] Nelson LA, Mulvaney SA, Johnson KB, et al. mHealth Intervention Elements and User Characteristics Determine Utility: A Mixed-Methods Analysis. *Diabetes Technol Ther* 2017; 19: 9–17.

[40] Hirschey J, Bane S, Mansour M, et al. Evaluating the Usability and Usefulness of a Mobile App for Atrial Fibrillation Using Qualitative Methods: Exploratory Pilot Study. *JMIR Hum Factors* 2018; 5: e13.

For peer review only

For peer review only - http://bmjopen.bmj.com/site/about/guidelines.xhtml

862 [41] Grindrod KA, Li M, Gates A. Evaluating User Perceptions of Mobile Medication Management
863 Applications With Older Adults: A Usability Study. *JMIR MHealth UHealth*; 2. Epub ahead of
864 print 14 March 2014. DOI: 10.2196/mhealth.3048.

865 [42] Nelson LA, Bethune MC, Lagotte AE, et al. The Usability of Diabetes MAP: A Web-delivered
866 Intervention for Improving Medication Adherence. *JMIR Hum Factors* 2016; 3: e13.

867 [43] Lucivero F, Jongsma KR. A mobile revolution for healthcare? Setting the agenda for bioethics.
868 *J Med Ethics* 2018; 44: 685–689.

869 [44] Lupton D. The digitally engaged patient: Self-monitoring and self-care in the digital health era.
870 *Soc Theory Health* 2013; 11: 256–270.

871 [45] Ramanathan N, Swendeman D, Comulada WS, et al. Identifying preferences for mobile health
872 applications for self-monitoring and self-management: focus group findings from HIV-positive
873 persons and young mothers. *Int J Med Inf* 2013; 82: e38-46.

874 [46] Dennison L, Morrison L, Conway G, et al. Opportunities and Challenges for Smartphone
875 Applications in Supporting Health Behavior Change: Qualitative Study. *J Med Internet Res*
876 2013; 15: e2583.

877 [47] Naughton F, Hopewell S, Lathia N, et al. A Context-Sensing Mobile Phone App (Q Sense) for
878 Smoking Cessation: A Mixed-Methods Study. *JMIR MHealth UHealth* 2016; 4: e106.

879 [48] Boonstra TW, Nicholas J, Wong QJ, et al. Using Mobile Phone Sensor Technology for Mental
880 Health Research: Integrated Analysis to Identify Hidden Challenges and Potential Solutions. *J*
881 *Med Internet Res* 2018; 20: e10131.

882 [49] Szinay D, Jones A, Chadborn T, et al. Influences on the Uptake of and Engagement With
883 Health and Well-Being Smartphone Apps: Systematic Review. *J Med Internet Res* 2020; 22:
884 e17572.

885 [50] Fortuna RJ, Nagel AK, Rocco TA, et al. Patient Experience With Care and Its Association With
886 Adherence to Hypertension Medications. *Am J Hypertens* 2018; 31: 340–345.

887 [51] Haskard Zolnierek KB, DiMatteo MR. Physician Communication and Patient Adherence to
888 Treatment: A Meta-Analysis. *Med Care* 2009; 47: 826–834.

889 [52] Harmon G, Lefante J, Krousel-Wood M. Overcoming barriers: the role of providers in
890 improving patient adherence to antihypertensive medications. *Curr Opin Cardiol* 2006; 21:
891 310–315.

892 [53] 1 Recommendations | Behaviour change: individual approaches | Guidance | NICE,
893 https://www.nice.org.uk/guidance/ph49/chapter/1-Recommendations (accessed 4 May
894 2021).

895 [54] Carson KV, Verbiest ME, Crone MR, et al. Training health professionals in smoking cessation.
896 *Cochrane Database Syst Rev*. Epub ahead of print 2012. DOI:
897 10.1002/14651858.CD000214.pub2.

898

For peer review only

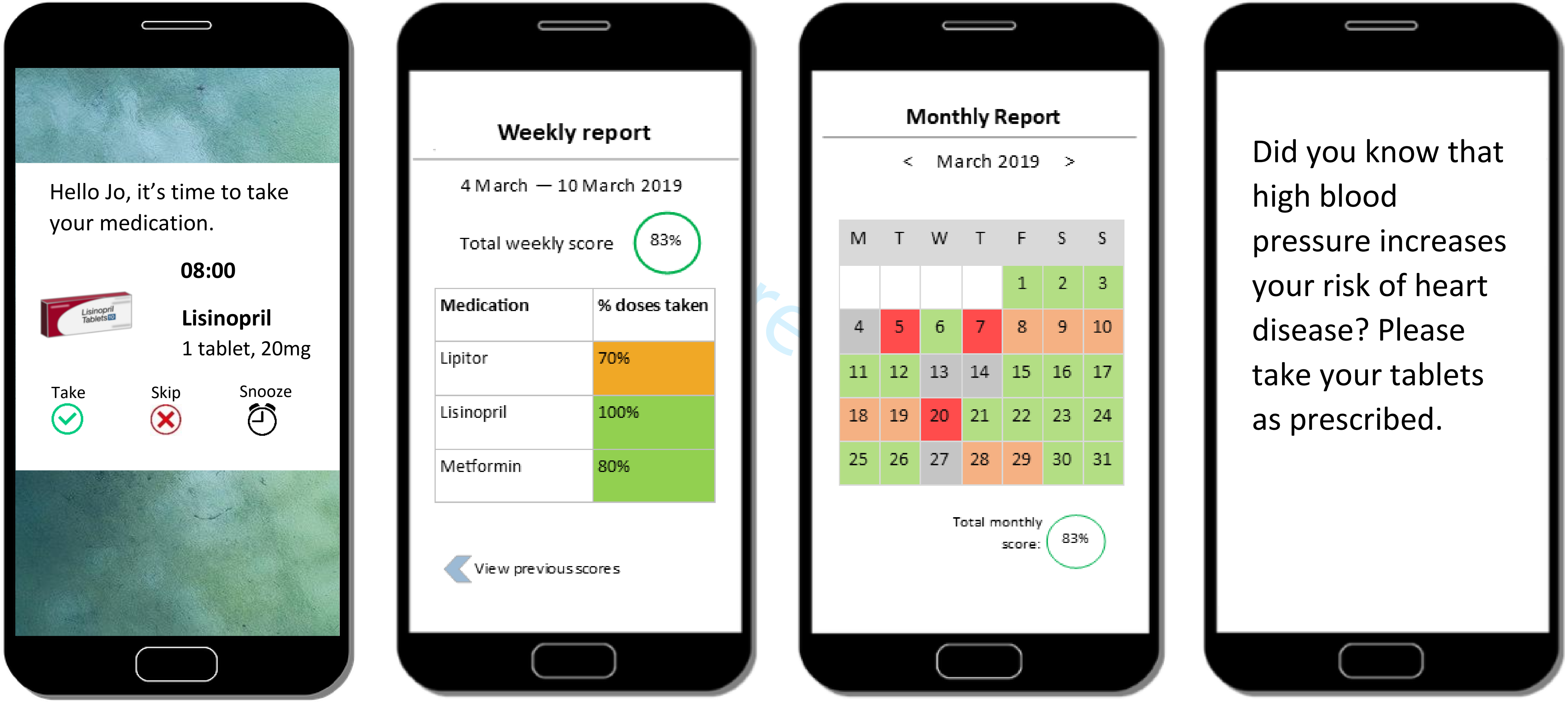
Hello Jo, it's time to take your medication.
08:00
Lisinopril
1 tablet, 20mg
Take
Skip
Snooze
Weekly report
4 March — 10 March 2019
Total weekly score
83%
Medication
% doses taken
Lipitor
70%
Lisinopril
100%
Metformin
80%
View previous scores
Monthly Report
< March 2019 >
M T W T F S S
1 2 3
4 5 6 7 8 9 10
11 12 13 14 15 16 17
18 19 20 21 22 23 24
25 26 27 28 29 30 31
Total monthly score:
83%
Did you know that high blood pressure increases your risk of heart disease? Please take your tablets as prescribed.

Supplementary file 1: Consolidated criteria for reporting qualitative studies (COREQ): 32-item checklist

**Item**	**Guide questions**	**Description**	**Reported on page no.**
**Domain 1: Research team and reflexivity**			
Personal Characteristics			
1. Interviewer/facilitator	Which author/s conducted the interview or focus group?	One author (MVE) conducted the semi-structured interviews. Two authors co-facilitated the focus groups (MVE and JJ).	8, 28
2. Credentials	What were the researcher's credentials? E.g. PhD, MD	BSc (MVE), BSc, PhD (JJ)	N/A
3. Occupation	What was their occupation at the time of the study?	Research Assistant (MVE) and Research Associate (JJ)	N/A
4. Gender	Was the researcher male or female?	Female (MVE) and male (JJ)	N/A
5. Experience and training	What experience or training did the researcher have?	MVE's training in qualitative research has included: BSc undergraduate modules; Oxford University Introduction to Qualitative Research; University of Cambridge Social Sciences Research Methods Centre training course. JJ is experienced in conducting focus groups (JJ)	N/A
Relationship with participants			
6. Relationship established	Was a relationship established prior to study commencement?	Participants had no relationship with the researcher. Patients contacted MVE via telephone or email to arrange participation in the study. HCPs* met with MVE face-to-face during a set up meeting at the GP practice, prior to interview.	7-8

For peer review only - http://bmjopen.bmj.com/site/about/guidelines.xhtml

7. Participant knowledge of the interviewer	What did the participants know about the researcher? e.g. personal goals, reasons for doing the research	Participants knew the reasons for conducting the research, as detailed in the patient information sheet and discussed prior to commencing the interview/focus group.	N/A
8. Interviewer characteristics	What characteristics were reported about the interviewer/facilitator? e.g. Bias, assumptions, reasons and interests in the research topic	Participants were aware that the research aimed to inform the development of an intervention to support medication taking. Researchers were unable to avoid bias regarding prior knowledge of the research literature around medication adherence.	N/A
**Domain 2: study design**			
Theoretical framework			
9. Methodological orientation and Theory	What methodological orientation was stated to underpin the study? e.g. grounded theory, discourse analysis, ethnography, phenomenology, content analysis	Analysis was informed methodologically by the constant comparative approach[1] and theoretically by Perceptions and Practicalities Approach (PAPA)[2].	9
Participant selection			
10. Sampling	How were participants selected? e.g. purposive, convenience, consecutive, snowball	Patients were eligible if they were prescribed medication to treat hypertension and used SMS messages or smartphone apps. A random sample of patients were selected by GP practice administrators to receive the study invitation pack. HCPs were eligible if they were involved with the management or care of patients with hypertension, including medication reviews or hypertension checks. HCPs were a convenience sample from the four GP practices taking part in the study.	7-8

For peer review only - http://bmjopen.bmj.com/site/about/guidelines.xhtml

11. Method of approach	How were participants approached? e.g. face-to-face, telephone, mail, email	Patients received an invitation letter from their GP practice, or saw study advertisement posters in the waiting room. HCPs were invited to take part during a face-to-face meeting with the researcher (MVE) at their GP practice.	7-8
12. Sample size	How many participants were in the study?	20 patients and 11 HCPs	11
13. Non-participation	How many people refused to participate or dropped out? Reasons?	All participants who responded to the study invitation and set a date/time for participation, took part in either an interview or focus group.	N/A
Setting			
14. Setting of data collection	Where was the data collected? e.g. home, clinic, workplace	Patient interviews: participant's homes (n=4), workplace (N =1) and a local library (n=1).<br>HCP interviews: their place of work (n=11 HCP)<br>Focus groups: local community centres (n=14, across 4 groups)	9
15. Presence of non-participants	Was anyone else present besides the participants and researchers?	Only the participants and researchers were present during the study.	N/A
16. Description of sample	What are the important characteristics of the sample? e.g. demographic data, date	Participant characteristics are reported in Tables 1 and 2.	Tables 1 and 2
Data collection			
17. Interview guide	Were questions, prompts, guides provided by the authors? Was it pilot tested?	The interview and focus group schedules were guided by the principles of PAPA and informed by the aims of the intervention development team. Interview and focus group schedules were reviewed by the research team and PPI representatives.	9-10, supplementary files 2a, 2b, 2c
18. Repeat interviews	Were repeat interviews carried out? If yes, how	No repeat interviews were carried out.	N/A

For peer review only - http://bmjopen.bmj.com/site/about/guidelines.xhtml

	many?		
19. Audio/visual recording	Did the research use audio or visual recording to collect the data?	Interviews and focus groups were audio-recorded.	10
20. Field notes	Were field notes made during and/or after the interview or focus group?	Field notes were made during and/or after each interview and focus group.	N/A
21. Duration	What was the duration of the interviews or focus group?	Mean interview duration was 47 minutes (range 21 to 75). Mean focus group duration was 88 minutes (range 83 to 91).	10
22. Data saturation	Was data saturation discussed?	Recruitment of participants continued until no new themes were emerging in relation to the specific topics covered.	12
23. Transcripts returned	Were transcripts returned to participants for comment and/or correction?	Transcripts were not returned to participants for comment and/or correction	N/A
**Domain 3: analysis and findings**			
Data analysis			
24. Number of data coders	How many data coders coded the data?	Data was coded by MVE and JJ, supervised by HE. Findings were discussed with the research team.	10-11, 28
25. Description of the coding tree	Did authors provide a description of the coding tree?	A sample of transcripts were read thoroughly to identify broad codes. Transcripts were compared in turn with codes already identified to refine the coding framework. Codes were then grouped into themes.	N/A
26. Derivation of themes	Were themes identified in advance or derived from the data?	We generated general descriptive categories from a subsample of transcripts. These were either derived directly from the data, or were pre-defined from the literature review, interview schedule questions and/or aims of the intervention development. This meant that some	10

For peer review only - http://bmjopen.bmj.com/site/about/guidelines.xhtml

		categories and concepts had increased sensitivity.	
27. Software	What software, if applicable, was used to manage the data?	Data was managed using NVivo 12.	10
28. Participant checking	Did participants provide feedback on the findings?	Participants did not provide feedback on the findings.	N/A
Reporting			
29. Quotations presented	Were participant quotations presented to illustrate the themes/findings? Was each quotation identified? e.g. participant number	Participant quotations are presented in the main text to illustrate the findings. Each quotation is identified using a participant ID. A table of extra quotations from participants is presented in the supplementary materials.	See Results section, and Supplementary file 2.
30. Data and findings consistent	Was there consistency between the data presented and the findings?	There was consistency between the data presented and the findings.	See Results section.
31. Clarity of major themes	Were major themes clearly presented in the findings?	Major themes are identified at the beginning of the Results section, and referred to throughout.	See Results section.
32. Clarity of minor themes	Is there a description of diverse cases or discussion of minor themes?	Minor themes are explored alongside the major themes.	See Results section.

* **Note:** HCP = healthcare practitioner

Developed from: Tong A, Sainsbury P, Craig J. Consolidated criteria for reporting qualitative research (COREQ): a 32-item checklist for interviews and focus groups. *International Journal for Quality in Health Care*. 2007. Volume 19, Number 6: pp. 349 – 357

References

1. Charmaz K. *Constructing Grounded Theory: A Practical Guide through Qualitative Analysis*. SAGE; 2006.
2. Horne R, Cooper V, Wileman V, Chan A. Supporting Adherence to Medicines for Long-Term Conditions. *Eur Psychol*. 2019;24(1):82-96. doi:10.1027/1016-9040/a000353

For peer review only - http://bmjopen.bmj.com/site/about/guidelines.xhtml

Supplementary file 2a

Example digital intervention messages

Medication reminder	Hello Jo, it's time to take your Lisinopril 20mg this morning. Thank you.
Feedback	Glad to see you're taking your blood pressure medication. Keep up the good work!
Information	Did you know that high blood pressure increases your risk of heart disease? Please take your tablets as prescribed.
Support	Have you taken all your prescribed tablets today? If you need support, reply 'HELP' to this number.

Note: Example messages were created as part of a wider NIHR-funded programme grant. For more information about the intervention content and functions, see Kassavou, A'Court, Chauhan et al (2020) https://pilotfeasibilitystudies.biomedcentral.com/articles/10.1186/s40814-020-00666-2

Topic guide: patient interviews

In the each interview, the interviewer will remind the participant of the project aims and check that they fully understand the participant information sheet, and will then take them through the informed consent process. Audio-recording of the interview will then begin.

Part 1: Questions to assess participant's experience with taking their medication

First I want to ask you about the medications that you're taking at the moment.

- How many medications have you been prescribed and what medications do you take?
    - How many doses do you need to take and how often?
- What would you say are the benefits of taking your medication?
- What about the downside of /problems with taking your medications?
    - How does this affect whether you take your medication or not?
- You said you're meant to take [XX] medications a day. How easy it is to remember to take them?
    E.g. prompts
    - Do you have a particular routine? If so, what is it?
- What about altering the dose, is this something that you ever do? [can you tell me more about that? Which medication? How do you alter it? Can you tell me why you alter it in that way? Can you think of any other reason why you don't take your tablets regularly? E.g. side effects]
- Is there anything that helps you to take you medication? Anything that makes it easier?

Part 2: Views on an intervention to support medication adherence

- Interviewer will outline the basic structure of the proposed intervention, using visual aid/schematic to help explain.

For peer review only - http://bmjopen.bmj.com/site/about/guidelines.xhtml

For peer review only

- A very brief face-to-face session with a practice nurse or healthcare assistant followed by ongoing support via mobile/internet.

- How would you feel about something like this that could support you to take your medication as prescribed?
  - Via text message?
  - Via an app?
- Which of these would be best for you? [e.g. prefer app or text?]
- What sort of messages would you find most helpful for helping you to take your tablets?

Example materials

- *Show participants examples of digital interventions*
  - Prompt questions
    - Which types of messages would you find most useful for helping you to take your medications? Why/why not?
    - What types of messages would be unhelpful? And why?
  - How could these messages be improved? Or reworded?
  - What messages would be useful for someone who doesn't want to take their pills? (i.e. INA)
  - How many messages per day or per week?

  - What about information / any other features that could be included? *E.g. probes:*
    - Feedback on your adherence level (e.g. graph, table % score, which prefer?)
    - Involve a carer/significant other?
    - Make use of location information

Part 3 – User engagement

- What would encourage you to start using something like this? *(Prompt initiation ideas such as demonstrating SMS/app during consultation, downloading app during consultation)*
  - Is there anything that would prevent or make it difficult for you using it?
  - Would a tutorial help you to understand how to use the SMS/app? How would you prefer to access this tutorial?
  - What would encourage you to keep using the app/text service long term?

For peer review only - http://bmjopen.bmj.com/site/about/guidelines.xhtml

Supplementary file 2b

Topic guide: healthcare practitioner interviews

In the each interview, the interviewer will remind the participant of the project aims and check that they fully understand the participant information sheet, and will then take them through the informed consent process. Audio-recording of the interview will then begin.

Part 1: Questions to ascertain participant's experience with addressing non-adherence issues with their patients

- Can you describe your role in the GP Practice?
- How involved are you with prescribing and monitoring patients' medication?
- How do you usually ascertain whether a patient is taking their medication as prescribed?
- What do you tend to do if/when you think a patient is not taking their medication as prescribed?

  E.g. probes:

  - Medication-taking can be perceived as a sensitive topic, how do you tend to address this when starting your conversation?
  - Do you explore patients' reasons for not taking it; how easy/difficult is this?
  - What challenges do you come across?
  - What do you think can help support patients in their medication taking?

Part 2: Views on an intervention to support medication adherence

Interviewer will outline the basic structure of the proposed intervention, using visual aid/schematic to help explain.

  - *A very brief face-to-face session with a practice nurse or healthcare assistant or pharmacist followed by (ii) ongoing support via mobile/internet.*
  - *Include a draft outline of the 5 minute face-to-face consultation between HCP and patient.*

- How comfortable/confident would you feel in delivering this?

  E.g. probes:

  - *Go through draft outline of VBI line by line.*
    - E.g. tailoring questionnaire
    - What information would you include in the pamphlet?
    - Is there anything you would add/remove?
  - What about doing this all in 5 minutes?
  - Would you find this task demanding? If yes, how could we make this easier for you? (E.g. tools/prompts/scripts- and how best to present these- bullet points?)
  - What kind of training would you find helpful? (E.g. multiple sessions/feedback/group work)

For peer review only - http://bmjopen.bmj.com/site/about/guidelines.xhtml

Interviewer will outline the idea of a digital intervention [possibly using visual aid/schematic to help explain how/when this would happen]

- What are your thoughts on how patients would get on with an intervention like this?
  - How useful do you think it would be in supporting patients to take their medications between their primary care consultations?
    - *E.g. probes:* helpful/unhelpful messages
  - What do you think patients need from an intervention like this?
  - If you were designing text messages/phone app, what content or materials would you include?

Part 3: Incorporating into a consultation

- What would encourage you to recommend a text message service/app to your patients?
  - What thoughts do you have on encouraging patients to use it / and keeping them engaged?
  - Would you find it helpful to trial the digital intervention yourself before recommending to patients?
- How might an intervention be incorporated into a consultation at the GP practice?
  - Which routine consultation would be appropriate?
  - What thoughts do you have on how to sign the patients up?
- Are there any specific patients who might benefit from an intervention more, or less, than others?

For peer review only - http://bmjopen.bmj.com/site/about/guidelines.xhtml

Supplementary file 2c

Topic guide: patient focus groups

Introduction

- Consent etc. (audio recording - ensure participants are comfortable with this before starting, and 'ground rules' of FGs.)
- Begin audio recording.
- Describe the PAM intervention. Will keep brief and in lay terms.
- We want to find out what you think about some of the app features

- Before we start, can I ask how many medications you've each been prescribed?
  - How many per day?
  - Morning/evening doses?
- How much you use your smartphones?
- Do you use any Apps that have reminder message pop up (e.g. calendar, med reminder app etc)?
  - Prompt: do these have alerts? How often do you use these apps?

1. Reminder notification messages

I'd like you to look at these examples [medication reminder messages]

- Go through each feature for each scenario (the text, layout, image, size, etc.) what do you like/dislike? What would you change?

Message content

- Is there anything missing from this message?
- What would you remove from this message?
- Which is your favourite and why?

Snooze options

Do you know what a snooze option is (e.g. like your alarm clock) [if not explain it's a feature we can add which allows you to delay the notification for extra time, similar to an alarm clock.]

- Let's say you're able to snooze the medication reminder message. How useful would you find this function? In which situations might you use it?
  - [prompts could include how long snooze should be etc.]

Images of medication

- Would you find it useful to have any of these images of your medications in the App?
- Would you like to be able to take your own photo and upload it?

Types of medication

- If you have more than one medication….

For peer review only - http://bmjopen.bmj.com/site/about/guidelines.xhtml

- How would you feel about receiving reminder messages ONLY for medication related to HBP?
- Would you want the option to choose which medications you receive reminders for?

- Do your medications ever change? What about if your medications change- how would you like to handle that?
  - Update the app yourself?

Message timings

- What time would you like to receive these messages?
  - Would you want to be able to change the timings yourself?
  - How comfortable would you feel doing this?
  - How about weekday vs weekends?

Message frequency

- When talking about reminder messages, how many of these would you like to receive per day?
- What about for non-reminder messages? (E.g. messages with advice and support to motivate people to take their medication)

Engagement and 'honest' reporting!

- We know that some people can get fed up with these types of apps, and messages on your phone every day.
- What would encourage you to use the app every day, say for a month? Or 3 months?
  - What would discourage you?
- What about stopping/reducing reminders?
  - When/why might you want the messages reducing and/or stopping?

- Some people might get a notification telling them a dose is due, think, "I want to skip this dose".
- How honest do you think people would really be when responding in the app?
  - How can we encourage people to use it 'honestly'
    - Are there any occasions where people might 'lie'?
    - Prompts (e.g. assure people that their GP won't see what they say)

2. Feedback of adherence

Apps can give you feedback on your results using different visual formats (e.g. table, graph, list, monthly calendar – show examples)

Prompts:

- Which would you prefer? And why?
- Total % score – helpful?
- Would you use this feature?
- What information do you want to see in the 'feedback'?
- What other methods could the app use to feedback reports?

For peer review only - http://bmjopen.bmj.com/site/about/guidelines.xhtml

- Would you find weekly or monthly feedback more helpful? Why?

3. Sensing data

- Now we would like to hear your thoughts on 'sensing technology'.
- Do you know much about the sensors in our smartphones?

- *[Facilitator explains sensors]*
- *All smartphones have sensors built into the device, which can gather data on how the phone is being used, such as movement and light (e.g. detect if it's face up or down).*
- *A simple example is when you make a call, and hold the phone to your ear, do you notice that the touchscreen automatically switches off, to stop you pressing buttons with your cheek. This happens because a sensor within the phone can detect when an object is close to its screen.*
- *Another example is how some phones have an inbuilt pedometer which can count the number of steps walked each day, by detecting the 'walking' movement of your phone.*

- *[Explain sensors re tailoring the App]*
- *Sensing technology is a novel way to personalise apps to your everyday behaviour.*
- *E.g. with this app, we can use sensors to personalise the messages sent to you, based on information collected from sensors in your phone.*
- *Provide examples of data that could be collected by the app*
- *Location data.*
    - *Detected either by WiFi (e.g. your home WiFi or a café WiFi) or GPS (detecting the location of the phone, like when we use Google Maps and satnavs).*
    - *The app could use this to send you messages at appropriate times, e.g. only when you're at home.*
- *Movement of phone or pedometer*
    - *e.g. App will detect movement of the phone (running/walking) and will delay a reminder until you've finished*

- How would you feel about an app collecting this data?
    - Would it put you off using the app?
- Is there anything that we could do to encourage you to like/change your mind about this feature?
- Is there anything you don't like about this feature?
- What questions would you have about this? (this will help us to inform the leaflet for ppts)
- What information should we provide for users? How detailed? How? (e.g. website/leaflet)

Close

For peer review only - http://bmjopen.bmj.com/site/about/guidelines.xhtml

Supplementary file 3

Draft outline of a 5-minute face-to-face consultation (VBI)

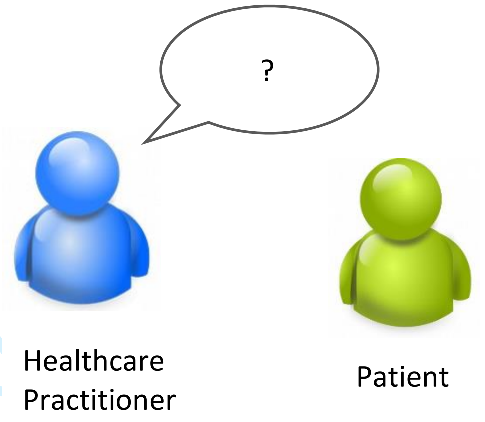

- Start the consultation with de-stigmatising medication non-adherence – forgetting to take tablets and/or having concerns is common.
- Support is available. Introduce the digital support (SMS texts or mobile phone app) as a (fun?) way to help patients take them – perhaps share personal experience with using it, demonstrate a key feature of the app/ text-messaging support.
- Complete short online questionnaire on behalf of patient:
    - Input basic information (patient name, age, mobile phone number)
    - Choose from drop down list of medications that patient is prescribed (tablet name, dose, frequency)
    - Ask patients to give their main reasons for not taking their medication (e.g. choose top 3 reasons from a list, or rank from most important to least important).
- Generate a code for patients to download the app/start the support. E.g. QR code, link to website for patients to download app.
- Hand out pamphlet with further information.
- Endorse the patients' use of digital support.
- Record in the system for follow-up purposes.

PAM HCP interviews VBI example

For peer review only - http://bmjopen.bmj.com/site/about/guidelines.xhtml

Supplementary file 4: Major themes, sub themes and illustrative quotes

Theme	Sub theme	Example participant quote
**Reasons for non-adherence**	*Non intentional non-adherence (NINA)*<br>Forget to take medication and/or reorder prescription	If I don't put the blister pack back and take the tablets, sometimes I can't remember if I've taken it or not [P03, male, 60s]<br><br>I just totally forgot to take my medication this morning. I just had a cup of tea and a piece of toast. I have to admit I was in a hurry today, I'm sorry, I didn't take the BP [medication]. [P06, female, 60s]<br><br>Usually it's that they haven't had a chance to put their prescription in, so then they tend to run low. [HCP 07, Practice Nurse, female]
	Intentional non-adherence (INA)<br>Unpleasant side effects; general reluctance to take medication	Sometimes I'm tempted to, and if I'm doing some sporting activity, I will be less inclined to take them. They seem to reduce my performance […] perhaps take it after I've indulged in the sport. [P04, male, 40s]<br><br>Often patients will say that they felt fine before they took their blood pressure medicine and now they feel rotten. [HCP 06, Practice Nurse, female]<br><br>Especially with metformin, because it can often have some unpleasant side-effects, and very often people will stop taking it because of this, but they don't actually report that fact to us. [HCP 10, Practice Nurse, female]<br><br>You know, I'm sure there's a lot of natural stuff out there that could help me you know. I don't know what effect it's having inside. That's the trouble, you know…what, in fact, it's doing. [P02, male, 60s]<br><br>I just hate myself for having to take them… I just feel that there must be a natural way… I just feel that, you know, lifestyle choices would be a better way than taking tablets, and that's why I hate myself for having to do it. [P03, male, 60s]

For peer review only - http://bmjopen.bmj.com/site/about/guidelines.xhtml

**Recommendations for intervention content**	Simple reminders to take medication and to reorder prescription (NINA)	It is good about the reminder because I sometimes say to my husband, er, would you remind me and he doesn't because he's worse than I am. [P05, female, 70s]<br><br>I think that would be quite good if it could tell you when you last got your prescription and when your next prescription's due and send a reminder maybe a few days before to say. [FG3, male]<br><br>Do you know what would be handy, is the reminder to tell you when you're gonna run out. That would be good for me. [FG1, male]
	Messages containing information about medication: consequences of non-adherence (targeting INA) acceptable to HCPs but not to patients; preference for advice on how to follow up a missed dose	I guess they need a more educational approach...that's the kind of need to explore why they are choosing not to take it. Do they understand the consequences of not taking it? So the nature of the messaging might be quite sort of shock, 'if you do not take your aspirin you are at risk of having a heart attack', or something like that. (HCP 02, GP, male)<br><br>It's like preaching, and I don't need my phone to be beeping at me in order to preach a message. [FG4, female]<br><br>I don't know whether I could...whether it's okay to do a catch-up, like if I missed my Ramipril in the morning, can I take that and the Indapamide at night? Or would that be too high a dose in one go? So, you know, maybe that needs to be addressed in the texts [P03, male, 60s]<br><br>Other days I've forgotten completely and then I get home and I think, "Shall I take 'em now or shall I wait 'til tomorrow now?" I've done that once or twice. (M)<br>That is a problem, whether you can take it if you've forgotten. (F)<br>I want to know precisely, "Well, will that affect it big or a lot or not a lot or...? And is taking them all together more important than the hour?" [...] It's the occasional expert knowledge that motivates people to stick with an app isn't it? [...] It's the fact that if you could press a button and say, "What should I do now 'cos I've forgotten 'em?" might be just a great help. (M) [FG3]

For peer review only - http://bmjopen.bmj.com/site/about/guidelines.xhtml

	Feedback on adherence levels – simple statistics preferred	That could be a graph, couldn't it? Or a percentage. You know, 'well done', you know, 'you're ninety-five per cent this month'. [P03, male, 60s]<br><br>Even a weekly graph might be quite nice. Because, then you can see that, actually, when I started a week ago, I was taking fifty per cent…and now I'm going up, and you know, just a visual kind of thing for them. And, actually, now I'm on a hundred per cent, and next week I'm hundred per cent. [HCP 01, Practice Pharmacist, female]<br><br>For me, I think the answer probably is yes actually, because actually that really would be a... you know, a wake-up call, thinking, "God, [participant name], do you realise that's two days this week?" I mean, okay, I should be able to tell what I had but, you know –. [FG4, male]
	Signpost patients to contact their HCP	If you are having side-effects, please contact somebody, rather than just ignore it…. It could be it's the pharmacist, or the Practice Pharmacist rather than the community one. It's just about improving interactions with us, the pharmacist, reporting side-effects. [HCP 06, Practice Nurse, female]
**Tailoring the digital intervention**	Highly tailored intervention facilitates engagement with SMS messages and smartphone app	It's about tailoring the process to the patients' needs….What suits one is not going to suit the next twenty people. [HCP 06, Practice Nurse, female]<br><br>It would be nice to be a bit personalised, it makes you feel a bit special [FG1, male]<br><br>You can default [medication reminders] to all the days of the week being the same but it might be nice to be able to specify different times of the day if you really wanted to. [FG3, male]<br><br>I mean, could users perhaps make their own decision which, which [reminder message] version they want to have appear? [FG4, female]<br><br>How much margin is there to vary from person to person? So if I said I wanted the full works I could have them but perhaps somebody that didn't? [FG1, male]

For peer review only - http://bmjopen.bmj.com/site/about/guidelines.xhtml

	Barrier to engagement – if intervention is overcomplicated or difficult to use	A hundred ideas that you get but if it's not simple then, the app in the end—From personal experience it won't work, it needs to be simple for people to buy into it. [FG2, male]<br><br>But I think it has to be really simple 'cos, you know, the more you're delving into this, it's... it can get complicated, can't it? So people are just gonna want something easy. [FG3, female]
**Acceptability of sensing technology**	Facilitator – Potential for sensing technology to enhance user experience; flexible opt-in/out options	Another time that I might forget to take medication is if I've gone somewhere just overnight ... I might just forget to put it in and I think, oh, well, nothing I can do, I'll have to wait 24 hours or whatever, but if you put in your diary on your phone that you were going to then [the app] might say, "don't forget to pack your pills". [FG2, male]<br><br>I would be happy with the sensing and respond accordingly because I think we could fine tune some of it ourselves couldn't we? If it was personalised. [FG2, female]<br><br>But could people have an off button for that if they wanted, for that bit of the menu? [FG2, female]
	Barrier to acceptability – concerns about confidentiality of data; requests for information about data use	It's all about how that information is dissembled, isn't it, and so long as that information is within reason locked away somewhere [FG4, male]<br><br>I would definitely want to know you weren't passing my data onto anyone else, either for medical or marketing purposes [FG2, female]<br><br>I'd feel much happier using one that wasn't purely commercial as well. [FG2, female]

For peer review only - http://bmjopen.bmj.com/site/about/guidelines.xhtml

**Attitudes towards a VBI (HCP only)**	Barriers and facilitators to implementation: must be straightforward to incorporate and deliver within primary care; five minute time limit potentially unfeasible; sufficient training in delivery of VBI	It would have to be something that's very accessible on your desktop or as part of integrated into the clinical system. [HCP 03, Practice Nurse, female]<br><br>I think this is quite a straightforward process, as I say, because a lot of this we should be doing already and then it's the add on bit at the end. [HCP 06, Practice Nurse, female]<br><br>I don't think it's going to end at five minutes. I think it's probably going to go a bit more than that in some cases [HCP 05, Healthcare Assistant, female]<br><br>I'm quite chatty so they will probably just chat with me, 'cause I make them chat, but it could be easily done in five minutes if you were concise and just got on with it. HCP 08, Practice Nurse, female]<br><br>It would be really good that we've had the training on how to use the app, how to use the questionnaire… It might be useful even just to show us how you might do a consultation. You know, sometimes visually seeing something can be… Even just a video. [HCP 01, Practice Pharmacist, female]<br><br>Quite often what works well is just an almost practical session of: "This is what we're proposing. This is how you do the face to face consultation. This is what the apps look like" and then, you know, it then develops discussion. [HCP 06, Practice Nurse, female]<br><br>The more you do and the easier would become and you get more slick at it, wouldn't you… What we would need is a little bit more information on the type of app and text that you are going to try and set up. And maybe have a dummy run and see how comfortable we felt with it and if there was anything we could do to make it slightly more slicker and more professional… A video clip or something of somebody doing it so we would know how to approach it and address it and stuff like that, will probably be the best thing. [HCP 09, Practice Nurse, female]

For peer review only

	Recommendations for VBI: importance of HCP/patient relationship; understand the root cause of non-adherence and address patient concerns	If we have a very judgmental approach to care then we actually lose the patient's confidence and their respect and actually they won't come back at all. So that is just so important.[HCP 06, Practice Nurse, female]<br><br>You can get through if you put it in the right wording. I do find, I've been doing it for quite a few years now and I find I have got a good, you know, I'm not blowing my own trumpet, but I have got quite a good rapport with my patients [HCP 04, Healthcare Assistant, female]<br><br>I just try to keep it open and honest, and sort of gain their trust so they actually tell me why [they aren't taking their medications]. Most patients, if they know you or if they've dealt with you before, are quite happy to share… If they, um, don't think that they're going to get into trouble [laughs] for not taking it. [HCP 11, Practice Pharmacist, female]<br><br>It's about sort of exploring why, what their understanding is of the medications that they are taking and trying to get an understanding of whether they recognise the importance of compliance. So it's kind of just exploring in a deeper way as to why they've not been taking. [HCP 02, GP, male]<br><br>I tend to just try and get to the bottom of [non-adherence]… It's unpicking what the problems are and what their perception is and sometimes it's quite misguided and it's different from one person to another. [HCP 03, Practice Nurse, female]<br><br>The reasons why patients don't take their medication we should really be exploring in any case… our role in the face to face is actually we can explore some of these things a bit easier. [HCP 06, Practice Nurse, female]

Note: HCP = healthcare practitioner

For peer review only - http://bmjopen.bmj.com/site/about/guidelines.xhtml
